# JACK trial protocol: a phase III multicentre cluster randomised controlled trial of a school-based relationship and sexuality education intervention focusing on young male perspectives

**DOI:** 10.1136/bmjopen-2018-022128

**Published:** 2018-07-28

**Authors:** Maria Lohan, Áine Aventin, Mike Clarke, Rhonda M Curran, Lisa Maguire, Rachael Hunter, Clíona McDowell, Lisa McDaid, Honor Young, James White, Adam Fletcher, Rebecca French, Christopher Bonell, Julia V Bailey, Liam O’Hare

**Affiliations:** 1 School of Nursing and Midwifery, Queen’s University Belfast, Belfast, UK; 2 Centre for Public Health, Queen’s University Belfast, Belfast, UK; 3 Northern Ireland Clinical Trials Unit, Belfast, UK; 4 Research Department of Primary Care and Population Health, University College London, London, UK; 5 MRC/CSO Social & Public Health Sciences Unit, University of Glasgow, Glasgow, UK; 6 The Centre for the Development and Evaluation of Complex Interventions for Public Health Improvement (DECIPHer), Cardiff University, Cardiff, UK; 7 British Heart Foundation Cymru; 8 Department of Social and Environmental Health Research, London School of Hygiene and Tropical Medicine, London, UK; 9 Department of Primary Care and Population Health, University College London; 10 Centre for Evidence and Social Innovation, Queen’s University Belfast, Belfast, UK

**Keywords:** public health, community child health, sexual medicine, preventive medicine

## Abstract

**Introduction:**

Teenage pregnancy remains a worldwide health concern which is an outcome of, and contributor to, health inequalities. The need for gender-aware interventions with a focus on males in addressing teenage pregnancy has been highlighted as a global health need by WHO and identified in systematic reviews of (relationship and sexuality education (RSE)). This study aims to test the effectiveness of an interactive film-based RSE intervention, which draws explicit attention to the role of males in preventing an unintended pregnancy by reducing unprotected heterosexual teenage sex among males and females under age 16 years.

**Methods and analysis:**

A phase III cluster randomised trial with embedded process and economic evaluations. *If I Were Jack* encompasses a culturally sensitive interactive film, classroom materials, a teacher-trainer session and parent animations and will be delivered to replace some of the usual RSE for the target age group in schools in the intervention group. Schools in the control group will not receive the intervention and will continue with usual RSE. Participants will not be blinded to allocation. Schools are the unit of randomisation stratified per country and socioeconomic status. We aim to recruit 66 UK schools (24 in Northern Ireland; 14 in each of England, Scotland and Wales), including approximately 7900 pupils. A questionnaire will be administered at baseline and at 12–14 months postintervention. The primary outcome is reported unprotected sex, a surrogate measure associated with unintended teenage pregnancy. Secondary outcomes include knowledge, attitudes, skills and intentions relating to avoiding teenage pregnancy in addition to frequency of engagement in sexual intercourse, contraception use and diagnosis of sexually transmitted infections.

**Ethics and dissemination:**

Ethical approval was obtained from Queen’s University Belfast. Results will be published in peer-reviewed journals and disseminated to stakeholders. Funding is from the National Institute for Health Research.

**Trial registration number:**

ISRCTN99459996

**Strengths and limitations of this study**

- The study is evaluating the first relationship and sexuality education (RSE) intervention to be developed and trialled, which explicitly promotes a gender-transformative approach to addressing teenage pregnancy by focussing on male perspectives and critical reflection on gendered norms.
- The intervention is culturally sensitive to different parts of the UK, non-directive in terms of pregnancy resolution options and sufficiently flexible to allow use within schools which vary in their personal development/RSE policy, including in faith-based schools.
- It is the first RSE Intervention to be developed and trialled across all four nations of the UK, allowing for exploration of what works best where.
- Due to the nature of the intervention and setting—within schools—participating teachers, pupils and parents cannot be blinded to allocation.
- A biological measure of adolescent conception rates was not possible and hence we rely on a surrogate measure of incidence of unprotected sex.

## Introduction

Teenage pregnancy remains a worldwide health concern and is both an outcome of, and contributor to, inequalities in health.^1^ The UK has the highest rate of teenage pregnancy in Western Europe.^2^ While conception rates for girls aged under 18 years have halved since 1998 in England and Wales, and now stand at 21.0 per 1000 population,^3^ it remains that just over 20 000 teenage women under 18 years became pregnant in England and Wales in 2015 and approximately half of these ended in legal abortion.^3^ The conception rate for Scotland was 32.4 per 1000 in 2015.^4^ In Northern Ireland (NI), abortion is illegal and is only considered lawful in exceptional circumstances where the life of the pregnant woman is at immediate risk or if there is a risk of serious injury to her physical or mental health. Reflecting this different legal framework, government targets around reducing teenage pregnancies in NI relate to births and not conceptions. In NI, the birth rate for teenage mothers per 1000 young women aged 13–19 years was 11.3 in 2013.^5^ In the same year, the teenage birth rate in the most deprived areas was 23.0 per 1000, nearly six times that of the least deprived areas in NI (3.9 per 1000).^6^

Although the life course for teenage parents is not universally negative,^7^ the social disadvantage and exclusion that are linked to teenage pregnancy are considered problematic.^1^ Unintended teenage pregnancy can lead to considerable adverse health problems for teenagers and their infants as well as generating emotional, social and economic costs for them, their families and society.^8 9^ While unintended teenage pregnancy is a complex phenomenon that cannot be prevented through relationship and sexuality education (RSE) alone,^10–16^ high-quality RSE is an essential component in the process of reducing unintended pregnancy rates, as well as being a vital aspect of improving holistic sexual health and well-being.^17–21^ The UK governments all emphasise the policy importance of decreasing under-18 conception rates and increasing sexual health precaution behaviours in teenagers via the implementation of RSE in schools as a key objective in their current sexual health policies.^22–24^

Several systematic reviews have identified the characteristics of effective RSE programmes, which help increase their impact on sexual risk-taking behaviours.^25–31^ These include the use of theoretically based interventions targeting sexual and psychosocial-mediating variables such as knowledge, attitudes, self-efficacy, intentions, perceptions of risk and perceptions of peer norms which are linked to sexual behaviour change; the use of culturally sensitive and gender-specific interventions; the use of interactive modalities which promote personal identification with the educational issues and engagement of young people; the use of skills-building components; the involvement of parents in the RSE process and facilitating linkages with support services. However, teenage boys have usually been neglected in relation to RSE, particularly with respect to teenage pregnancy.^18 32–36^ The lack of gender-sensitive interventions which acknowledge the potential influence of gender in successfully engaging both males and females in addressing teenage pregnancy has been highlighted as a global health need by WHO^37–39^ and identified in systematic reviews of RSE.^15 35 40 41^

The *If I were Jack* teacher-led classroom-based RSE intervention represents an innovative combination of the effective characteristics identified in the above-mentioned systematic reviews. It is aimed at both teenage boys and girls but with explicit attention drawn to the role of teenage boys in preventing an unintended teenage pregnancy. A specific aim of *If I Were Jack* is to encourage scrutiny of the gender norms, which typically situate the issue of a teenage pregnancy as a woman’s problem, by placing emphasis on teenage male perspectives while not excluding teenage female perspectives. The *If I Were Jack* intervention is predicted to decrease young people’s sexual risk-taking behaviour in relation to avoiding teenage pregnancy and to promote positive sexual health.

We aim to evaluate the effectiveness and cost-effectiveness of the *If I Were Jack*, RSE intervention in reducing rates of unprotected sex among teenagers under 16 years of age and to better understand the contextual conditions through a process evaluation. The intervention will be delivered to replace some of usual RSE in the intervention group. Schools in the control group will not receive the intervention and will continue to deliver RSE according to their current and existing practices, including meeting their statutory curriculum requirements. This is a pragmatic comparator reflecting typical routine practice, which allows for comparison of the intervention with the existing RSE experience.

## Methods and analysis

### Trial design

**Figure 1:**
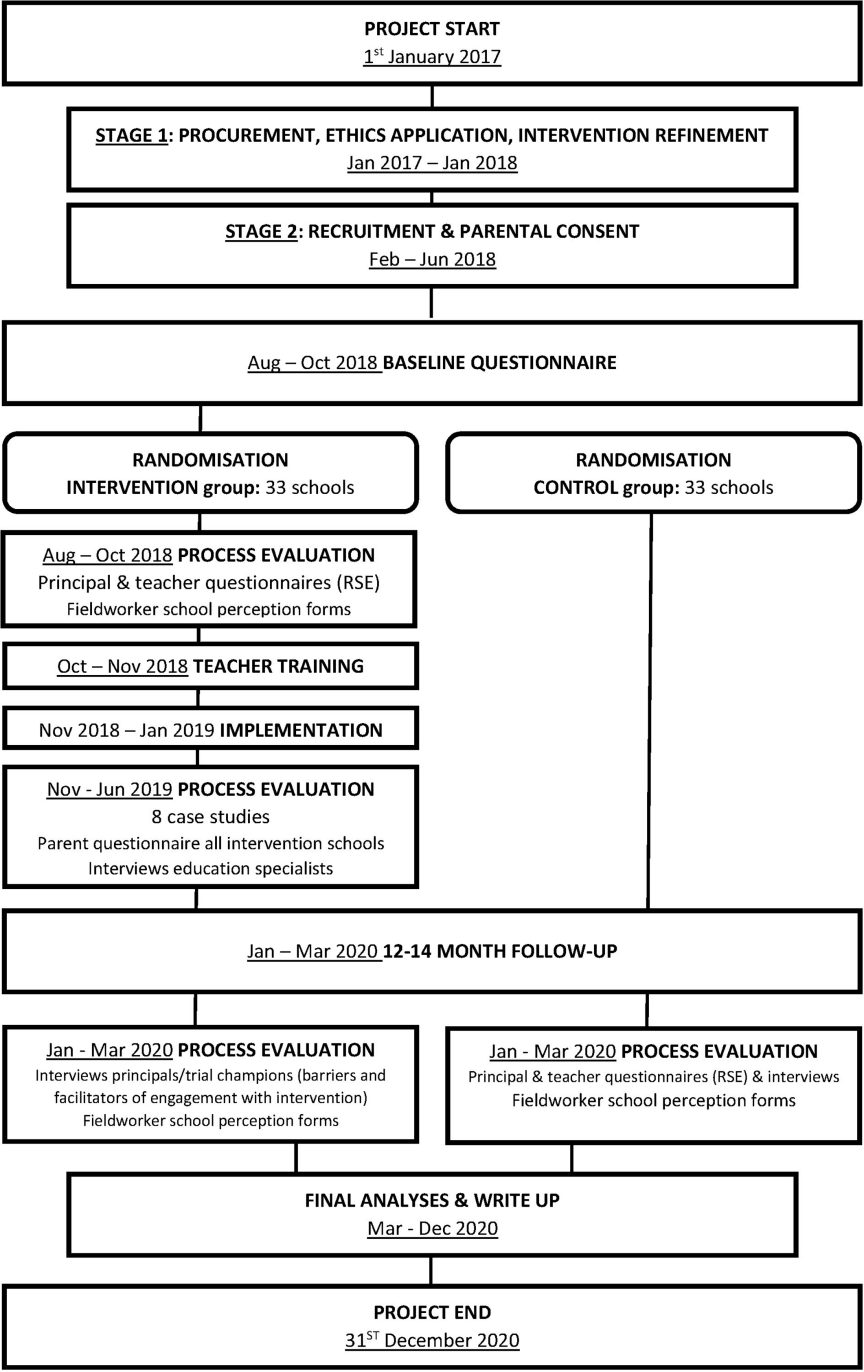
The JACK trial flow chart. RSE, relationship and sexuality education.

The JACK trial is a phase III multicentre, parallel-group cluster randomised trial (cRCT) (figure 1). Schools are the unit of randomisation with a 1:1 allocation. The study design has an embedded process evaluation and economic evaluation. The trial design follows the Medical Research Council’s Framework for Developing and Evaluating Complex Interventions.^42^

### Study setting

The trial will take place in 66 secondary level schools in the UK (24 in NI and 14 each in England, Scotland and Wales). The whole of NI is included but, for reasons of practicality, convenience and cost, representative geographical restrictions will be in place in England (Greater London area), Scotland (mainland Scotland) and Wales (South Wales). The intervention will be delivered by teachers, as part of the Key Stage 4 Personal and Health Education curriculum (NI, England and Wales) and in Scotland as part of the Curriculum for Excellence Relationships, Sexual Health and Parenthood education.

### Public involvement

Public involvement in the design of the *If I were Jack* intervention has been facilitated by a Young People’s Advisory Group (YPAG) composed of members from each of the four nations of the UK. The group of 20 members aged 14–16 years and their designated youth workers was brought together for a facilitated discussion about the intervention during one weekend in Cardiff in April 2017. The group contributed especially to production decisions for the interactive video drama. This YPAG also read and commented on the questionnaire and pupil information sheet remotely. In the earlier feasibility trial, we had consulted extensively with pupils about the questionnaire.^43^ The intervention and trial design has also been informed by a Trial Stakeholders Group. This group is composed of relationship and sexuality education experts and teachers and senior representatives from key statutory organisations and government departments from all four nations of the UK. Finally, the trial design has been informed by a Trial Steering Group, composed of methodological experts, pupils, teachers and school principals/head teachers. All three groups will continue to advise the research team throughout the trial. Dissemination to schools will first involve discussion with schools and our YPAG involving regular updates and final reports. Our research team across the four nations will disseminate at talks aimed at the public and policymakers in all four jurisdictions and a lay summary will be made available on our Jack trial website http://www.qub.ac.uk/sites/if-i-were-jack/

### Eligibility

#### Eligibility criteria for clusters

All state secondary-level schools in the 2018/2019 academic year will be included with the exception of independent private, special and Irish/Welsh-medium and Scottish Gaelic schools (but not excluding schools that have an embedded Irish/Welsh-medium component). Schools with <30 pupils in the target year group (year 11 in NI, S3 in Scotland and year 10 in England and Wales) will be excluded. Schools that have already participated in the feasibility (n=8 in NI),^43^ transferability (England n=3, Scotland n=3 and Wales n=3) and pilot studies (England n=1, Scotland n=1 and Wales n=1) involving the *If I Were Jack* intervention in preparation of this for phase III study will also be excluded.

#### Eligibility criteria for participants

Eligible teachers are those who will be responsible for the delivery of RSE to pupils in year 11 in NI, S3 in Scotland and year 10 in England and Wales during the 2018/2019 academic year.

Eligible pupils are the 2018/2019 academic year pupil cohort (all classes within) in year 11 in NI, S3 in Scotland and year 10 in England and Wales (mean age 14). This year group has been selected for a number of reasons. First, proximal risk factors of teenage pregnancy begin manifesting in this age group,^14 44^ making it an appropriate time for preventative sex education that is considered acceptable in society and education.^14 27 45^ Second, there is an identified deficit in resources for this age group in relation to teenage pregnancy,^46 47^ and third, findings from the JACK feasibility study^43^ indicated that there is a greater opportunity for implementation of the intervention during a year where there are no statutory examinations. Finally, this population has been chosen to facilitate a 12–14 months follow-up of pupils (postintervention) before some pupils exit formal education following their first major statutory exams or reaching the age of 16 years.

### Sample size

The sample size calculation is based on UK-wide data^48 49^ demonstrating that between 25% and 33% of 15 years old are having sex and the proportion of 15 years old reporting unprotected sex is 2.8% (overall in NI, England, Scotland and Wales). The study will be powered to detect a 50% reduction in the incidence of unprotected sex (from expected rate of 2.8% to 1.4%) by 15 years of age. Such a difference of 1.4% in unprotected sex has been shown to have a meaningful impact on pregnancy rates.^14 50–52^ The between-group difference in the incidence of unprotected sex of 1.3% (95% CI 0.5% to 2.2%) by 9 months in our feasibility trial^43^ demonstrates that such an effect size is plausible and is consistent with effect sizes seen in the literature.^50^ The study will take account of clustering. In the feasibility data, the intraclass correlation coefficient (ICC) was 0.01.^43^ As pilot studies can provide imprecise estimates of ICCs,^53^ we re-estimated using ICCs from three sources, the RIPPLE cRCT,^52^ a 2013/2014 WHO Health Behaviour in School-aged Children survey^48^ and a 2013 Young Persons’ Behaviour and Attitudes Survey in NI conducted by the Northern Ireland Statistics & Research Agency (NISRA).^49^ The data from WHO and NISRA studies were combined. The Randomised Intervention of a PuPil-led sex education trial (RIPPLE) and combined WHO and NISRA studies found an ICC of 0.004. Assuming 120 students per school, an ICC of 0.01% and 7% attrition (plus two additional schools to be conservative), a trial involving 33 schools per group will provide 80% power at a 5% significance level (a pupil participant sample size of n=7904, with n=224 reporting unprotected sex). The power would rise to 93% if the ICC is 0.004.

### Recruitment and retention

#### Recruitment of schools

In each country, eligible schools will be stratified based on the percentage of students eligible for free school meals (%FSM) for the 2018/2019 academic year (schools above and below the median national %FSM) as a proxy for the level of deprivation. In NI, 14 schools will be randomly selected from the above-median stratum and 10 from the below-median stratum (total 24). In England, Scotland and Wales, 8 schools will be randomly selected from the above-median stratum and 6 from the below-median stratum (to give a total of 14). The decision to select slightly more schools from the above-median %FSM reflects research which indicates that teenage pregnancy and unprotected sex is more acute in more deprived areas.^1 48^

**Figure 2:**
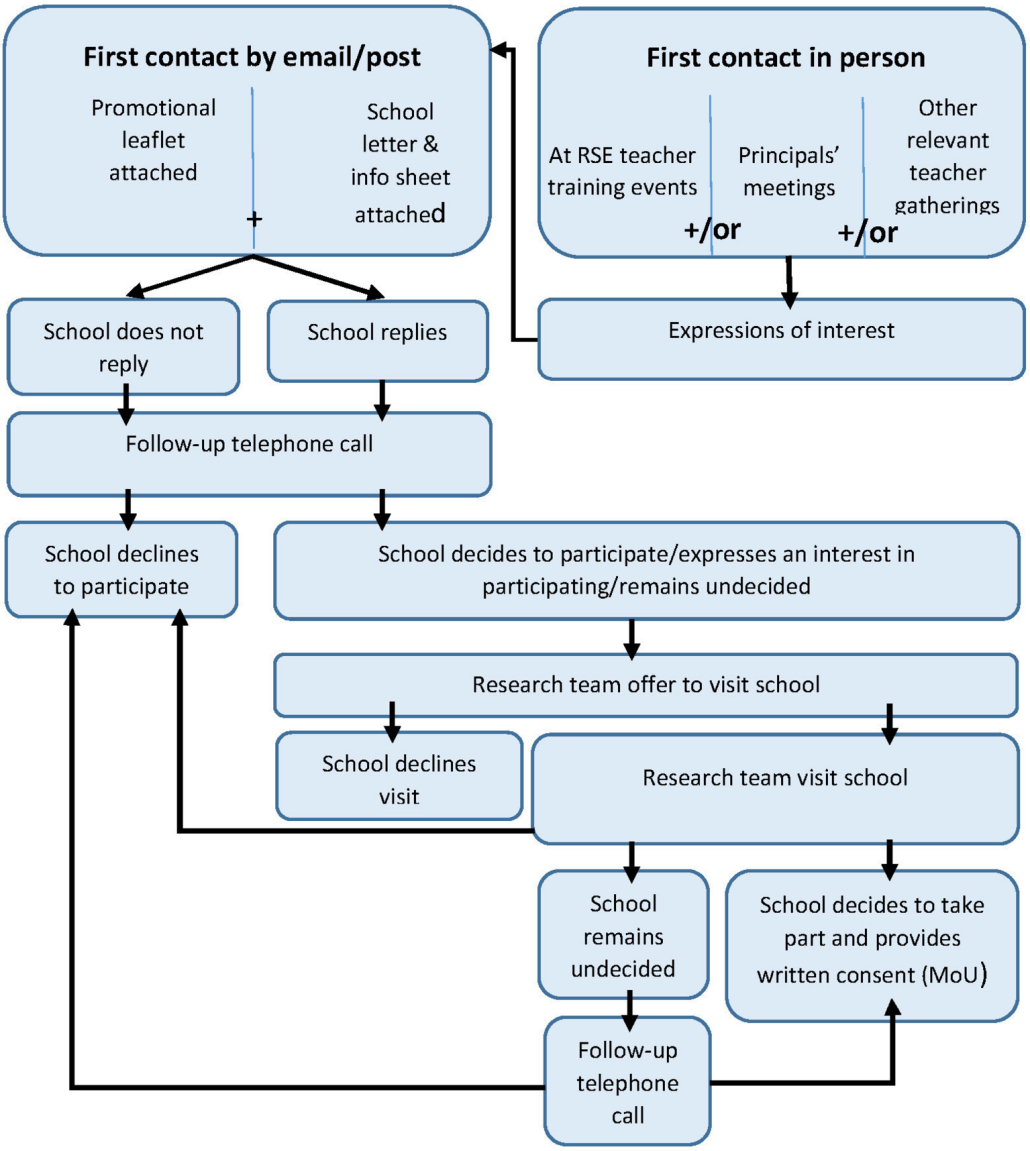
School recruitment strategy. RSE, relationship and sexuality education.

The school recruitment period will run from February to June 2018. The school recruitment strategy is represented in figure 2. In Scotland, it is required that permission is given from each local authority (typically by approaching the Director of Education) prior to commencing recruitment. Any schools that decline to participate will be replaced by a randomly selected school in the same stratum.

#### Recruitment of teachers

Based on our recruitment procedures in the feasibility study,^54^ once a school has made a decision to participate, a member of the research team will meet with teachers (identified by a school-assigned ‘Trial Champion’) responsible for the delivery of RSE to the target pupil year groups during the 2018/2019 academic year, to deliver an information session and answer any outstanding enquiries. Teachers will be provided with a copy of the school letter, information sheet, memorandum of understanding and consent form.

#### Recruitment of pupils

When a school and the relevant teachers who will be delivering the intervention to pupils have provided consent, the first step taken towards pupil recruitment is to inform parents/guardians. Schools (with the assistance of the school administrator) will be asked to post a hard copy of the parents’/guardians’ information sheet and an opt-out consent form with prepaid response envelopes. Schools will be responsible for addressing and preparing envelopes for postage. Parents/guardians will be advised within the material provided that they have to return the opt-out consent forms by a date no later than 3 weeks prior to commencement of baseline data collection within the school. The trial coordinator will collate a list of parents/guardians who have opted their child out of participation and return this to participating teachers.

Schools will be provided with printed copies of the pupil information sheets to be distributed to eligible pupils at least 1 week prior to baseline data collection. Only eligible pupils whose parents/guardians did not opt-out of providing consent for them to participate will be provided with a copy of the pupil information sheet. Immediately prior to administering the baseline questionnaire, eligible pupils will attend a short information session, delivered by a member of the research team, including an information video. Pupils will be given an opportunity to ask questions prior to deciding whether to participate. A repeat information session and baseline data collection session will be facilitated in agreement with the school to accommodate any pupils who are absent from the initial session. In the unlikely event that absenteeism remains in excess of 5%–10% in a school, the research team, in agreement with the school, will return a third time to facilitate an additional information session and baseline data collection. Pupils with mild learning difficulties or poor English will be supported where possible by fieldworkers to complete the questionnaires.

#### Retention

To promote school, teacher and pupil retention and complete follow-up, schools will be provided with £1000 on completion of baseline and follow-up measures. Trial coordinators will be proactive in resolving any issues that arise with schools. Periodic communications will be provided by trial coordinators to inform schools, staff, pupils and parents (depending on preferences of schools) of the current status of the study, and plans for the next phase, as well as to acknowledge their support.

### Randomisation

Randomisation will be carried out by the NI Clinical Trials Unit (NICTU, a UK Clinical Research Collaboration registered CTU), who will produce eight randomisation schedules (using unique identifiers for schools), one for each %FSM stratum within each country, using random permuted blocks of mixed size, generated using nQuery Advisor 7.0. The NICTU are not involved with recruitment and will only release the randomisation code (in sealed envelopes) when all schools have been recruited and baseline data collection completed, therefore allocation concealment will be ensured.

### Intervention

The intervention will be described in accordance with the Template for Intervention Description and Replication (TIDieR) guidelines.^55^

#### Name and brief description

**Figure 3:**
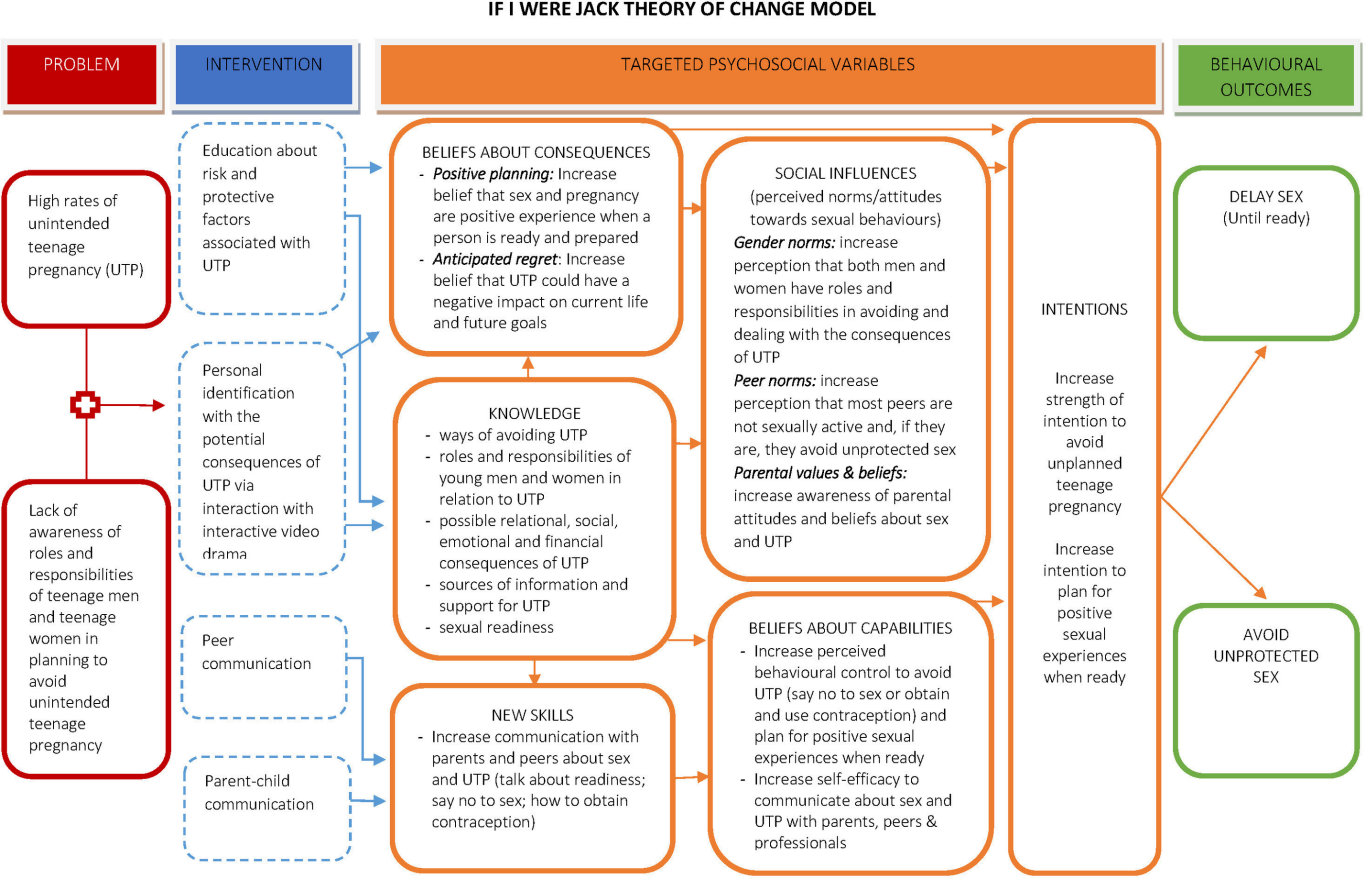
*If I Were Jack* theory of change logic model.

*If I Were Jack* is an evidenced-based RSE teacher-delivered intervention designed to prevent unintended pregnancy and promote positive sexual health by increasing teenagers’ intentions to avoid teenage pregnancy through delaying sexual intercourse or using contraception consistently. It is especially designed to engage with males but can be delivered to both male and female pupils. The underpinning theoretical framework for this intervention combines the well-established Theory of Planned Behaviour^56^ and critiques to this theory,^57^ which focus on the inclusion of an understanding of the broader socioenvironmental factors (such as socioeconomic status (SES)) and underlying values (such as religiosity and gender ideologies) associated with the occurrence of teenage pregnancy.^14 58 59^ The *If I Were Jack* theory of change logic model is depicted in figure 3.

#### Why, rationale of essential elements

*If I Were Jack* targets six psychosocial mechanisms which research indicates are related to a reduction in risk-taking behaviour: knowledge, communication skills, attitudes, social influences, beliefs about capabilities and intentions (table 1).^15 60 61^

**Table 1:** Psychosocial and behavioural components of the *If I Were Jack* intervention

Component	Aim
Knowledge	Increase knowledge of: ways of avoiding unintended pregnancy; roles and responsibilities of young men in relation to unintended pregnancy; possible negative relational, social, emotional and financial consequences of unintended pregnancy and sources of information and support for unintended pregnancy and sexual health more broadly.
Communication skills	Increase skills in communicating with parents, peers and sexual partners about avoiding unintended pregnancy.
Attitudes	Increase anticipated regret about the consequences of unintended pregnancy on current and future goals.
Social influences	Increase awareness of peer norms, stereotypical gender norms and parental attitudes and beliefs about teenage pregnancy. Gender norms: increase perception that both men and women have roles and responsibilities in avoiding and dealing with the consequences of unintended pregnancy. Peer norms: increase perception that most peers are not sexually active and use contraception when they are. Parental values and beliefs: increase awareness of parental attitudes and beliefs about unintended pregnancy.
Beliefs about capabilities	Increase perceived behavioural control to avoid unintended pregnancy (say no to sex or obtain and use contraception correctly) and increase self-efficacy to communicate about avoiding unintended pregnancy with parents, peers and professionals.
Intentions	Increase strength of intention to avoid unplanned teenage pregnancy.

The intervention components provide pupils with educational information and opportunities for discussion, skills practice, reflection and anticipatory thinking and are designed to specifically target one or more of the above psychosocial mechanisms.^62^ The intervention components also include explicit reference to the impact of SES, religion and gender norms on sexual behaviour, inviting participants to think through how underlying social influences, such as social class and gender norms of sexual behaviour, can be challenged through individual agency. A feasibility trial demonstrated the acceptability to teachers and pupils and feasibility of implementation across a wide range of schools in NI.^43^

#### What, a description of the materials

The *If I Were Jack* intervention consists of the following elements:

- A culturally sensitive interactive video drama (IVD) intended to immerse teenagers in a story of a week in the life of Jack, a teenager who has just been told by his girlfriend that she is pregnant. By asking males and females to imagine they were Jack and how they would think and feel if they were in his situation, it is designed to expose and challenge the gender assumptions around roles and responsibilities for teenage pregnancy by opening them up for reflection and negotiation. Informed by the findings of a prior transferability study that followed the feasibility trial of the intervention,^45^ two versions of the IVD have been made available: one for use in England and Wales, using actors with English accents and one for use in NI and Scotland, using actors with NI accents. The IVD is designed to be delivered on individual computers/tablets with the use of headphones.
- Classroom materials for teachers containing detailed lesson plans with specific classroom-based and homework activities designed to build pupils’ skills to a) obtain relevant sexual health information and b) develop communication skills with peers and trusted adults.
- A standardised 60 min training session for RSE teachers implementing the intervention. The training session will adhere to a predefined teacher-trainer protocol and will be delivered in schools by country-specific established statutory and non-statutory RSE coordinators who normally provide RSE teacher training in schools.
- Two short animated films to engage parents/guardians and help/encourage them to have a conversation with their teenager about avoiding unintended pregnancy. A link to the web-hosted films will be texted and/or emailed via a school administrator to all parents/guardians of participating pupils in intervention schools (with one additional reminder text/email).
- A dedicated website (www.qub.ac.uk/IfIWereJack) for the intervention will act as a portal of dissemination, providing password protected access to the intervention materials that teacher-trainers, teachers, parents and pupils can access.

#### Who delivers the intervention?

It is designed to be delivered by trained RSE teachers.

#### How, modes of delivery

*If I Were Jack* can be delivered either over four 50–60 min lessons or over six 35–45 min lessons and consists of a combination of classroom-based activities (mainly group discussion) having first viewed the IVD, in addition to pupils being asked to engage in two homework activities (one of which involves discussion with parents/guardians). Adherence to the intervention protocol will be determined as part of our process evaluation.

#### Where, locations where intervention has occurred

The intervention has been delivered in NI^43^ and Ireland, using a further locally produced IVD for Ireland. A version of the intervention, the IVD only, has been delivered in schools in South Australia.

### Outcomes

#### Primary outcome

In this trial, a reduction in unintended teenage pregnancy rates would be the ideal primary outcome measure, but the sample size would need to be very large to detect change in unintended pregnancy rates. We will therefore use a surrogate measure associated with unintended teenage pregnancy: unprotected sex at last sexual encounter, as defined by sexual intercourse without use of contraception (barrier or hormonal). Unprotected sex during teenage years is well established as the main proximate behavioural determinant of teenage pregnancy and is a commonly measured behavioural outcome in studies examining the impact of RSE interventions on teenage pregnancy.^10 12 14 50 63–65^ Studies indicate that, although other behavioural determinants (such as frequency of sexual intercourse and number of sexual partners) are important, avoidance of unprotected sex via consistent use of contraception is central in explaining variation in levels of teenage pregnancy.^18 51^ The primary outcome will be based on contraception use at last sexual intercourse (ie, answers to the question "Did you use any form of contraception the last time you had sex?”), consistent with the data on which the sample size calculation was based.^48^ An additional item will also be included related to lifetime incidence of unprotected sex in order to account for sporadic use of contraception that may not be reflected in the last sexual encounter. Participants reporting the use of natural family planning or withdrawal methods will be categorised as having engaged in unprotected sex due to the reduced efficacy of these methods in preventing pregnancy and transmission of sexually transmitted infections (STIs). This study will not undertake any data linkage with Health and Social Care or National Health Service records, given that data on conception rates are not available in NI and that data for sexual health-related services in England are not readily available as part of routinely collected data given patient privacy requirements.

#### Secondary outcomes

Secondary outcomes are 12-month impacts on knowledge, attitudes, skills and intentions to avoiding teenage pregnancy. Secondary outcomes informed by our theory of change (figure 3) include knowledge, attitudes, communication skills and intentions relating to avoiding teenage pregnancy at follow-up and are hypothesised to lead to increased intention to avoid unprotected sex. Data will be collected using a number of standardised measures, including comfort communicating about pregnancy and comfort communicating about contraception derived from mathtech behaviour inventory^65^; the male role attitudes scale^66^; sexual socialisation instrument^67^ and sexual self-efficacy scale.^68^ We will also collect data using an ‘intentions to avoid a teenage pregnancy scale’, developed and psychometrically tested in our feasibility trial.^43^ The measures were selected because the constructs they measure map closely to the theoretical framework underpinning the intervention and the reliability and completion rates of the measures were satisfactory in the feasibility trial.^43^ In addition, to assist with the economic evaluation, supplementary secondary outcomes include: frequency of engagement in sexual intercourse, contraception use, diagnosis of STIs and incidence of pregnancy and pregnancy outcomes. The collection of these data was also shown to be feasible in the feasibility trial. Finally, we will collect important individual level demographic and socioeconomic characteristics of the sample to deepen understandings of how these factors moderate effectiveness.

### Data collection

Participating pupils will be in the study for approximately 18 months and asked to complete a paper-based questionnaire during one RSE lesson at baseline and again between 12 and 14 months later. A fieldworker will administer questionnaires to pupils, under exam conditions, 2 weeks prior to commencement of intervention delivery. Informed by the process evaluation conducted in the feasibility study,^43^ teachers will be asked to stay at the front of the classroom to maintain order while alleviating any concerns that teachers may be able to see pupils’ answers. Additional fieldworkers will be available to provide support to pupils who require extra help and to ensure questionnaires are completed confidentially.

### Primary outcome

#### Process evaluation

**Figure 4:**
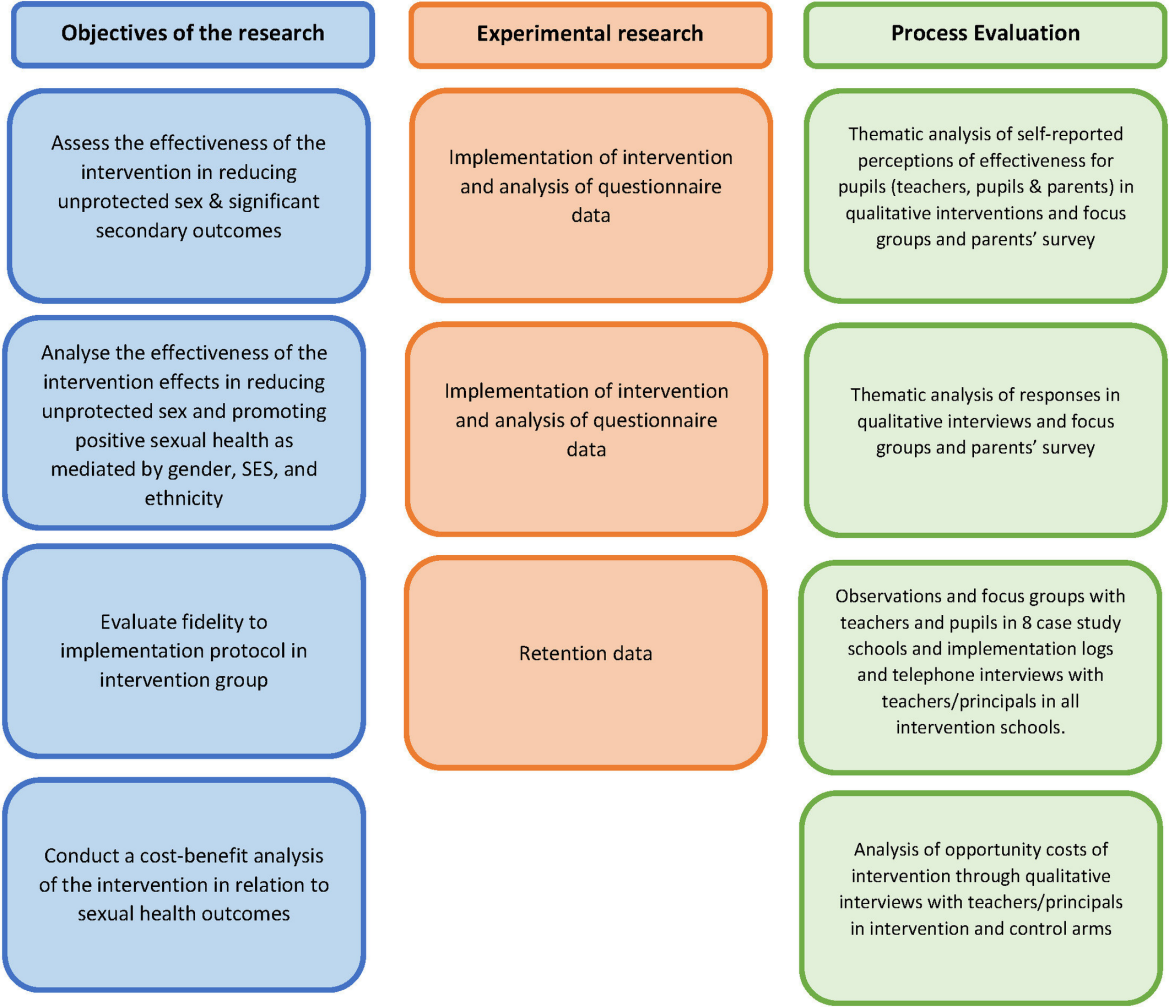
Integration of process evaluation with experimental design methodology to achieve research objectives. SES, socioeconomic status.

Informed by realist approaches to the evaluation of interventions,^42 69 70^ the process evaluation has four aims. First, we will examine reasons for school participation and non-participation to inform risk of bias in the trial as well as long-term sustainability of implementation of the intervention. Second, we will examine intervention delivery and fidelity in the context of overall RSE provision in intervention schools. Third, we will assess provision in control schools and potential contamination caused by any changes to provision that could be due to participation in the trial. Fourth, we will explore self-reported perceptions of effectiveness and moderating influences in intervention schools among a sample of pupils, teachers and school principals and parents. Triangulated data collection methods will include semi-structured interviews with teachers, focus group discussions with pupils, observations of a sample of lessons and a survey of parents/guardians with follow-up focus groups. For detail on our approach to integration of the process evaluation with the experimental design methodology to achieve research objectives, see figure 4.

##### All schools

The school-assigned trial champion or a suitable person identified by the trial champion will complete a school background questionnaire, designed to detail more general information pertinent to trial implementation, that is, school experience of teenage pregnancy, school holidays/closures, school experience of pupils/parents or guardians who do not speak English as a first language or who do not understand English at all and school involvement in other research. An appropriate member of staff identified by the school-assigned trial champion will also complete a questionnaire about current RSE provision in the school to gain a better understanding of the nature, quantity and quality of RSE currently taught within the school as well as the facilitators and barriers to current RSE provision within the school. A school administrator will be asked to fill out a school administrator resource use record detailing associated costs (ie, postage of parent/guardian information) and time spent. Schools will be reimbursed up to the value of £100 for these costs.

##### Intervention schools

*Parent/guardian online survey*: parents/guardians will have been made aware when they were in receipt of the parents’/guardians’ information sheet that there would be an opportunity for them to respond to a short online parent/guardian survey. Eligible parents/guardians (who have not opted out of providing consent for their child to participate) will receive the link to this survey (hosted using the SurveyMonkey UK platform) in an email and/or text message issued by the school administrator inviting them to participate (postintervention delivery). The survey will ask parents/guardians about their engagement with and opinion of the parent/guardian animations and homework session and whether their child has discussed with them their experience of engaging with *If I Were Jack*. The questionnaire will be translated where possible and where necessary.

*Teacher implementation log*: teachers responsible for delivering the intervention will be asked to complete an implementation log to detail what activities were completed or not completed during each lesson, and perception of pupil engagement with each activity.

*Telephone interviews*: 15–30 min telephone interviews will be conducted by trial coordinators with school principals or trial champions in intervention schools that are not ‘case study schools’ (see below) to determine any barriers or facilitators of engagement with the intervention.

##### Case study schools

Participating intervention schools will be randomly rank ordered in each country and two case study schools from each country will be randomly selected by NICTU to participate in the process evaluation. Should a school refuse participation, a further random selection will be made.

*Observations*: country-specific trial coordinators will conduct structured observations of one randomly selected lesson in every class group in receipt of the intervention in the eight case study schools. Observations will be focused primarily on measuring teacher fidelity to implementation protocol and pupil engagement.

*Focus groups*: trial coordinators will conduct three 60 min focus group discussions in each of the eight case study schools. One group will be composed of all teachers who delivered the intervention. The second group will include a maximum of six English-speaking pupils who received the intervention. Teachers who delivered the intervention will ask for a mixture of male and female pupil volunteers and pass details of those pupils to the trial coordinator. In the event that more pupils volunteer than are needed (per school), a random selection will be made. The third group will be a maximum of six English-speaking parents/guardians (of children who received the intervention). Discussions will focus on perceived barriers and facilitators of successful implementation and engagement with different components of the intervention.

##### Fieldworkers

Fieldworkers will complete a fieldworker perception form after each visit to a school, asking them to detail what worked well and what did not in relation to data collection and any other relevant observations they may have.

##### Education/policy specialists

Trial coordinators will conduct telephone or face-to-face interviews with one or two education/policy specialists in each country. Interviews will focus on the current context of RSE policy and perceptions of how this might influence the uptake of the *If I Were Jack* intervention.

### Economic evaluation

The economic evaluation will aim to describe the costs and consequences of implementing *If I Were Jack* in UK schools so as to provide information to decision makers on the implications of potential further roll-out of the intervention. This will include the duration of time taken up by *If I Were Jack* in school from the perspective of the teacher and impact on time spent on other important curricula activities compared with time spent on standard RSE. The aim of this will be to provide a measure of the opportunity cost to schools of implementing *If I Were Jack* compared with current RSE. The structure of the evaluation will follow the National Institute for Health and Care Excellence (NICE) guidance for evaluating public health interventions^71^ and recent guidance published by Edwards *et al* 72 on economic evaluations in public health. Costs will include the cost of implementing the intervention in schools including any training involved and the cost of current RSE in the control schools. We will also collect information on healthcare cost information in the intervention and control arms including the costs of sexual health-related primary care attendances, costs associated with STIs and unintended pregnancies (although numbers of these are likely to be small). The cost of adapting *If I Were Jack* to different groups will also be reported given that others may want to adapt the intervention before rolling it out. Mean cost per pupil will be reported alongside consequences including use of contraception, STIs and unintended pregnancies collected using questionnaires administered to pupils at baseline and follow-up. Although 12–14 months recall time is a relatively long time period, pupils are likely to be able to recall high impact events that occurred during this period. The follow-up time is also important to fit within the school year timetable. Costs will also be reported by country, given the different sexual health services provided and hence differential implications for health service costs by country.

**Figure 5:**
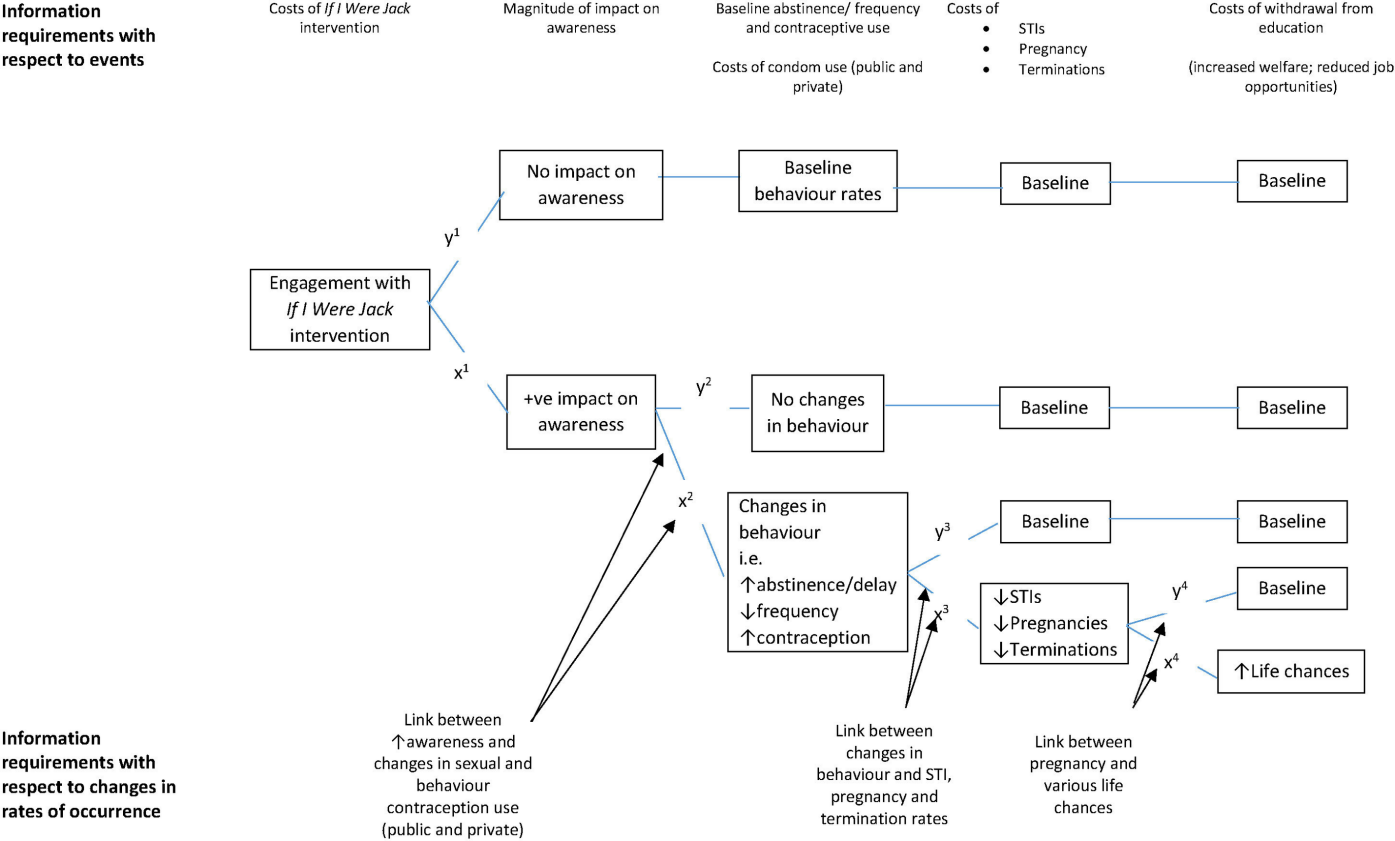
Current and future impacts of *If I Were Jack* on costs and benefits. STI, sexually transmitted infection.

Given that STIs and unintended pregnancies are likely to be rare but potentially high impact events in this population group, the long-term costs and consequences will be modelled as part of the cost-effectiveness decision model (figure 5), incorporating theories of behaviour change and identified as applicable for use in this trial during the feasibility trial.^45^ In addition to collecting information as part of the trial, we will look to systematic reviews of evidence of the impact of digital interventions on sexual health behaviour in this population group, for example, the review recently undertaken and published by Bailey *et al*.^73^ We will undertake one-way, two-way and probabilistic sensitivity analyses of the results. Cost-effectiveness acceptability curves and cost-effectiveness planes will be reported. The model will have a 20-year time horizon and discounting of future costs and benefits will comply with NICE guidance for evaluating public health interventions.^71^

### Statistical methods

The reporting and presentation of findings will be in accordance with the Consolidated Standards of Reporting Trials (CONSORT) guidelines for cRCTs.^74^ All analyses will take account of clustering by school using robust SEs, and intervention and control groups will be compared at baseline via frequencies/descriptive statistics (percentage, mean or median as appropriate) in relation to sex, ethnicity, SES at school level (using %FSM and postcode data) and at individual level (using highest education qualifications of parents), primary and secondary outcomes.

Primary analysis (12–14 months follow-up): the primary effectiveness analysis will be on an intention-to-treat basis, using a multilevel logistic regression model (two levels: pupils nested within schools) adjusting for the baseline outcome and stratification variables.^75^ Sensitivity analyses, making different assumptions on the best and worst case scenarios, as well as imputation models of missingness will be conducted to investigate the potential impact of missing data.

Secondary analysis (12–14 months follow-up): although the trial is not powered to detect the influence of mediating and moderating variables, we will examine the following outcomes informed by our theory of change model (figure 3): (i) interaction terms will be used to investigate possible differences in the effect of the intervention on the primary outcome by whether pupils at baseline reported having had unprotected sex or not, country (Wales, England, Scotland, NI), sex, socioeconomic group (see earlier section 5.5) and ethnicity); (ii) a mediational analysis, using an analytic framework recommended for RCTs,^76^ will be used to explore whether the effect of the intervention on the primary outcome is mediated by individual-level sexual health knowledge and sexual competence, perceived behavioural control, intentions to avoid an unintended pregnancy, communication with parents and gender ideologies. In these secondary analyses, p values will be interpreted with caution due to the low power and number of interactions being tested (eg, use of Bonferroni corrected p values).

#### Process evaluation

All data will be transcribed verbatim (in the case of interviews) or written up in detail (in the case of observational field notes and other secondary source data). These data will be organised using ‘NVivo’ software and analysed systematically and thematically based on the six steps proposed by Braun and Clarke^77^ to enable identification and analysis of patterns (or ‘themes’) within the data by moving iteratively between theoretical understandings and the new data. These inductively and deductively derived codes will be analysed to form overarching themes emerging from each of the participant groups outlined above. We will use qualitative software ‘NVivo 10’ to organise the data, and we will ensure methodological rigour by establishing credibility, transferability, dependability and confirmability using techniques suggested by Lincoln *et al*.^78^ In addition, following Hyde *et al*,^79^ specific attention will be given to analysing the group dynamics of the focus groups as part of the overall interpretive process.

## Discussion

The strengths of this study include that this is the first RSE intervention to be developed and trialled which explicitly promotes a gender-sensitive approach to addressing teenage pregnancy by focussing on male perspectives and a gender-transformative approach by encouraging males to share reproductive responsibility. The intervention is culturally sensitive to different parts of the UK, is non-directive in terms of pregnancy resolution options and is sufficiently flexible to be taught within the framework of a school’s ethos and personal development/RSE policy, including in faith-based schools. This has particular significance in NI, with almost half (46%) of all NI grammar and secondary schools identifying as a Roman-Catholic school.^80^ This is also the first RSE intervention to be developed and trialled across all four nations of the UK, allowing for exploration of what works best where. An additional strength of the study is that the embedded process evaluation involves triangulation of sources including school management, teachers, pupils, parents and RSE statutory and voluntary stakeholders. Study limitations include that the pragmatic setting —within schools—means that schools and participating teachers, pupils and parents will remain unblinded to the allocation. Finally, the use of the surrogate measure of unprotected sex rather than a biological measure (such as conception rates) introduces the possibility that the findings of the trial will be influenced by self-report bias, but the veracity of this measure is enhanced by privacy, confidentiality and a control group.

## Ethics and dissemination

In writing this protocol, we have endeavoured to adhere to the recommendations and guidance provided in the CONSORT 2010 statement and the extension for cRCTs,^74 81^ the Standard Protocol Items: Recommendations for Intervention Trials Statement 2013^82 83^ and the TIDieR guidelines.^55^ When registering the trial, we have provided structured summary information in accordance with the requirements of the WHO Trial Registration Data Set.^84^

Any modifications to the protocol which may impact on the conduct of the study, potential effectiveness or impact study participants, including changes of study objectives, study design, sample size, study procedures or significant administrative aspects will require a formal amendment to the protocol. Such amendment will be agreed on by the JACK Trial Steering Committee, reported to the funder (National Institute of Health Research (NIHR)) and approved by the ethics committee prior to implementation. Minor administrative changes, corrections or clarifications that have no effect on the way the study is to be conducted will be agreed on by the JACK Trial Steering Committee, documented and reported to the NIHR. The ethics committee may be notified of such changes at the discretion of the JACK Trial Steering Committee.

A full study report will be submitted to the NIHR by the end of December 2020 and made publicly available thereafter in the Public Health Research journal on the NIHR Journals Library. We shall make data available to the scientific community with as few restrictions as feasible, following receipt of a request to the corresponding author, while retaining exclusive use until the publication of major outputs via academic conference presentations and journal articles in addition to material created for relevant stakeholders. Pupil-friendly brief reports will be provided to all participating schools.

### Safety and data monitoring

This is a low-risk study, therefore, a Data Monitoring Committee is not required and no interim analysis is planned.

## Supplementary Material

Reviewer comments

Author's manuscript
