# Supplementary material for: JACK trial protocol: a phase III multicentre cluster randomised controlled trial of a school-based relationship and sexuality education intervention focusing on young male perspectives
Source: BMJ Open. 2018 Jul 28;8(7):e022128. doi: 10.1136/bmjopen-2018-022128 (PMC6067362; doi:10.1136/bmjopen-2018-022128)
Supplement: Author's manuscript [file bmjopen-2018-022128.draft_revisions.pdf]

BMJ Open is committed to open peer review. As part of this commitment we make the peer review history of every article we publish publicly available.

When an article is published we post the peer reviewers' comments and the authors' responses online. We also post the versions of the paper that were used during peer review. These are the versions that the peer review comments apply to.

The versions of the paper that follow are the versions that were submitted during the peer review process. They are not the versions of record or the final published versions. They should not be cited or distributed as the published version of this manuscript.

BMJ Open is an open access journal and the full, final, typeset and author-corrected version of record of the manuscript is available on our site with no access controls, subscription charges or pay-per-view fees (http://bmjopen.bmj.com).

If you have any questions on BMJ Open's open peer review process please email info.bmjopen@bmj.com

BMJ Open

The JACK Trial Protocol: A Phase III multi-centre cluster randomised controlled trial of a school-based relationship and sexuality education intervention focusing on young male perspectives

Journal:	*BMJ Open*
Manuscript ID	bmjopen-2018-022128
Article Type:	Protocol
Date Submitted by the Author:	09-Mar-2018

Complete List of Authors:	Lohan, Maria ; Queens University of Belfast,<br>Aventin, Áine; Queens University of Belfast<br>Clarke, Mike; Queen's University Belfast, Northern Ireland Methodology Hub<br>Curran, Rhonda; Queens University of Belfast<br>Maguire, Lisa; Queens University of Belfast<br>Hunter, Rachael; University College London, Research Dept of Primary Care and Population Health<br>McDowell, Clíona; Northern Ireland Clinical Trials Unit<br>McDaid, Lisa; University of Glasgow, MRC/CSO Social and Public Health Sciences Unit<br>Young, Honor; Cardiff University, Centre for the Development and Evaluation of Complex Interventions<br>White, James; Cardiff University, South East Wales Trials Unit<br>Fletcher, Adam; Cardiff University, School of Social Sciences<br>French, Rebecca ; London School of Hygiene and Tropical Medicine, Social and Environmental Health Research<br>Bonell, Christopher; London School of Hygiene and Tropical Medicine, Public Health and Policy<br>Bailey, Julia; University College London, Primary Care and Population Health<br>O'Hare, Liam; Queens University of Belfast

Keywords:	PUBLIC HEALTH, Community child health < PAEDIATRICS, SEXUAL MEDICINE, PREVENTIVE MEDICINE

SCHOLARONE™
Manuscripts

The JACK Trial Protocol: A Phase III multi-centre cluster randomised controlled trial of a school-based relationship and sexuality education intervention focusing on young male perspectives

Maria Lohan,[1] Áine Aventin,[1] Mike Clarke,[2,3] Rhonda M Curran,[1] Lisa Maguire,[1] Rachael Hunter,[4] Cliona McDowell,[3] Lisa McDaid,[5] Honor Young,[6] James White,[6] Adam Fletcher,[6] Rebecca S French,[7] Chris Bonell,[7] Julia Bailey,[7] Liam O'Hare[8]

1. School of Nursing and Midwifery, Queen's University Belfast, Belfast, UK

2. Centre for Public Health, Queen's University Belfast, Belfast, UK

3. Northern Ireland Clinical Trials Unit, Belfast, UK

4. Research Department of Primary Care and Population Health, University College London, London, UK

5. MRC/CSO Social & Public Health Sciences Unit, University of Glasgow, Glasgow, UK

6. The Centre for the Development and Evaluation of Complex Interventions for Public Health Improvement (DECIPHer), Cardiff University, Cardiff, UK

7. Department of Social and Environmental Health Research, London School of Hygiene and Tropical Medicine, London, UK

8. Centre for Evidence and Social Innovation, Queen's University Belfast, Belfast, UK

Corresponding Author: Prof Maria Lohan, School of Nursing and Midwifery, Queen's University Belfast, 97 Lisburn Road, Belfast, BT9 7BL, UK Email: m.lohan@qub.ac.uk Telephone: +44(0) 289 90972839

Word count: 6471, including funding statement and acknowledgments & excluding title page, abstract, references, figures and tables

For peer review only - http://bmjopen.bmj.com/site/about/guidelines.xhtml

ABSTRACT

Introduction Teenage pregnancy remains a worldwide health concern which is an outcome of, and contributor to, health inequalities. The need for gender-sensitive interventions with a focus on males in addressing teenage pregnancy has been highlighted as a global health need by the World Health Organisation and identified in systematic reviews of relationship and sexuality education (RSE). This study aims to test the effectiveness of an interactive film-based RSE intervention which draws explicit attention to the role of males in preventing an unintended pregnancy by reducing unprotected heterosexual teenage sex among males and females under age 16 years.

Methods and analysis A Phase III cluster randomised trial with embedded process and economic evaluations. *If I Were Jack* encompasses a culturally sensitive interactive film, classroom materials, a teacher-trainer session and parent animations, and will be delivered to replace some of the usual RSE for the target age group in schools in the intervention group. Schools in the control group will not receive the intervention and will continue with usual RSE. Participants will not be blinded to allocation. Schools are the unit of randomisation stratified per country and socio-economic status. We aim to recruit 66 UK schools (24 in Northern Ireland; 14 in each of England, Scotland and Wales), including approximately 7900 pupils. A questionnaire will be administered at baseline and at 12-14 months post-intervention. The primary outcome is reported unprotected sex, a surrogate measure associated with unintended teenage pregnancy. Secondary outcomes include knowledge, attitudes, skills and intentions relating to avoiding teenage pregnancy in addition to frequency of engagement in sexual intercourse, contraception use, and diagnosis of sexually transmitted infections.

Ethics and dissemination Ethical approval was obtained from Queen's University Belfast. Results will be published in peer-reviewed journals and disseminated to stakeholders. Funding is from the National Institute for Health Research.

Trial registration ISRCTN: 99459996; Pre-results

For peer review only - http://bmjopen.bmj.com/site/about/guidelines.xhtml

STRENGTHS AND LIMITATIONS OF THIS STUDY

- The study is evaluating the first relationship and sexuality education (RSE) intervention to be developed and trialled which explicitly promotes a gender-sensitive approach to addressing teenage pregnancy by focussing on male perspectives.
- The intervention is culturally sensitive to different parts of the UK, non-directive in terms of pregnancy resolution options and sufficiently flexible to allow use within schools which vary in their personal development/RSE policy, including in faith-based schools.
- It is the first RSE Intervention to be developed and trialled across all four nations of the UK, allowing for exploration of what works best where.
- Due to the nature of the intervention and setting – within schools – participating teachers, pupils and parents cannot be blinded to allocation.
- A biological measure of adolescent conception rates was not possible and hence we rely on a surrogate measure of incidence of unprotected sex.

For peer review only

For peer review only - http://bmjopen.bmj.com/site/about/guidelines.xhtml

INTRODUCTION

Teenage pregnancy remains a worldwide health concern and is both an outcome of, and contributor to, inequalities in health.[1] The UK has the highest rate of teenage pregnancy in Western Europe.[2] While conception rates for girls aged under 18 have halved since 1998 in England and Wales, and now stand at 21.0 per 1000 population,[3] it remains that just over 20,000 teenage women under 18 became pregnant in England and Wales in 2015 and approximately half of these ended in legal abortion.[3] The conception rate for Scotland was 32.4 per 1000 in 2015.[4] In Northern Ireland (NI), abortion is illegal and is only considered lawful in exceptional circumstances where the life of the pregnant woman is at immediate risk or if there is a risk of serious injury to her physical or mental health. Reflecting this different legal framework, government targets around reducing teenage pregnancies in NI relate to births and not conceptions. In NI, the birth rate for teenage mothers per 1000 young women aged 13-19 years was 11.3 in 2013.[5] In the same year, the teenage birth rate in the most deprived areas was 23.0 per 1000, nearly six times that of the least deprived areas in NI (3.9 per 1000).[6]

Although the life course for teenage parents is not universally negative,[7] the social disadvantage and exclusion that are linked to teenage pregnancy are considered problematic.[1] Unintended teenage pregnancy can lead to considerable adverse health problems for teenagers and their infants as well as generating emotional, social and economic costs for them, their families and society.[8, 9] While unintended teenage pregnancy is a complex phenomenon that cannot be prevented through Relationship and Sexuality Education (RSE) alone,[10–16] high quality RSE is an essential component in the process of reducing unintended pregnancy rates, as well as being a vital aspect of improving holistic sexual health and wellbeing.[17–21] The UK governments all emphasise the policy importance of decreasing under-18 conception rates and increasing sexual health precaution behaviours in teenagers via the implementation of RSE in schools as a key objective in their current sexual health policies.[22–24]

For peer review only - http://bmjopen.bmj.com/site/about/guidelines.xhtml

Several systematic reviews have identified the characteristics of effective RSE programmes which help increase their impact on sexual risk-taking behaviours.[27–33] These include: the use of theoretically-based interventions targeting sexual and psycho-social mediating variables such as knowledge, attitudes, self-efficacy, intentions, perceptions of risk, and perceptions of peer norms which are linked to sexual behaviour change; the use of culturally-sensitive and gender-specific interventions; the use of interactive modalities which promote personal identification with the educational issues and engagement of young people; the use of skills-building components; the involvement of parents in the RSE process; and facilitating linkages with support services. However, teenage boys have usually been neglected in relation to RSE, particularly with respect to teenage pregnancy.[18, 34–38] The lack of gender-sensitive interventions which acknowledge the potential influence of gender in successfully engaging both males and females in addressing teenage pregnancy has been highlighted as a global health need by the World Health Organisation (WHO)[39-41] and identified in systematic reviews of RSE.[15, 37, 42-43]

The *If I were Jack* teacher-led classroom-based RSE intervention represents an innovative combination of the effective characteristics identified in the above mentioned systematic reviews. It is aimed at both teenage boys and girls but with explicit attention drawn to the role of teenage boys in preventing an unintended teenage pregnancy. A specific aim of *If I Were Jack* is to encourage scrutiny of the gender norms which typically situate the issue of a teenage pregnancy as a woman's problem, by placing emphasis on the teenage male perspective whilst not excluding the teenage female perspective. The *If I Were Jack* intervention is predicted to decrease young people's sexual risk-taking behaviour in relation to avoiding teenage pregnancy and to promote positive sexual health.

We aim to evaluate the effectiveness and cost effectiveness of the *If I Were Jack* RSE intervention in reducing rates of unprotected sex among teenagers under 16 years of age and to better understand the contextual conditions through a process evaluation. The intervention will be delivered to replace

For peer review only - http://bmjopen.bmj.com/site/about/guidelines.xhtml

some of usual RSE in the intervention group. Schools in the control group will not receive the intervention and will continue to deliver RSE according to their current and existing practices, including meeting their statutory curriculum requirements. This is a pragmatic comparator reflecting typical routine practice, which allows for comparison of the intervention with the existing RSE experience.

METHODS AND ANALYSIS

Trial design

The JACK trial is a Phase III multi-centre, parallel-group cluster randomised trial (cRCT) (Figure 1). Schools are the unit of randomisation with a 1:1 allocation. The study design has an embedded process evaluation and economic evaluation. The trial design follows the Medical Research Council's Framework for Developing and Evaluating Complex Interventions.[44]

Study setting

The trial will take place in 66 secondary level schools in the UK (24 in Northern Ireland and 14 each in England, Scotland and Wales). The whole of NI is included but, for reasons of practicality, convenience and cost, representative geographical restrictions will be in place in England (Greater London area), Scotland (mainland Scotland) and Wales (South Wales). The intervention will be delivered by teachers, as part of the Key Stage 4 Personal and Health Education curriculum (NI, England and Wales) and in Scotland as part of the Curriculum for Excellence Relationships, Sexual Health and Parenthood education.

Eligibility

Eligibility criteria for clusters

For peer review only - http://bmjopen.bmj.com/site/about/guidelines.xhtml

All state secondary-level schools in the 2018/2019 academic year will be included with the exception of independent private, special and Irish/Welsh-medium and Scottish Gaelic schools (but not excluding schools that have an embedded Irish/Welsh-medium component). Schools with less than 30 pupils in the target year group (Year 11 in NI, S3 in Scotland and Year 10 in England and Wales) will be excluded. Schools that have already participated in the feasibility (*n*=8 in NI), [45] transferability (England *n*=3, Scotland *n*=3 and Wales *n*=3) and pilot studies (England *n*=1, Scotland *n*=1 and Wales *n*=1) involving the *If I Were Jack* intervention in preparation of this for phase III study will also be excluded.

Eligibility criteria for participants

Eligible teachers are those who will be responsible for the delivery of RSE to pupils in Year 11 in NI, S3 in Scotland and Year 10 in England and Wales during the 2018/2019 academic year.

Eligible pupils are the 2018/2019 academic year pupil cohort (all classes within) in Year 11 in NI, S3 in Scotland and Year 10 in England and Wales (mean age 14). This year group has been selected for a number of reasons. First, proximal risk factors of teenage pregnancy begin manifesting in this age group,[14, 46] making it an appropriate time for preventative sex education that is considered acceptable in society and education.[14, 29, 47] Second, there is an identified deficit in resources for this age group in relation to teenage pregnancy,[48-49] and third, findings from the JACK feasibility study[45] indicated that there is a greater opportunity for implementation of the intervention during a year where there are no statutory examinations. Finally, this population has been chosen to facilitate a 12-14 month follow-up of pupils (post-intervention) before some pupils exit formal education following their first major statutory exams or reaching the age of 16 years.

Sample size

The sample size calculation is based on UK-wide data[25-26] demonstrating that between 25% and 33% of 15 year olds are having sex and the proportion of 15 year olds reporting unprotected sex is

For peer review only - http://bmjopen.bmj.com/site/about/guidelines.xhtml

2.8% (overall in NI, England, Scotland and Wales). The study will be powered to detect a 50% reduction in the incidence of unprotected sex (from expected rate of 2.8% to 1.4%) by 15 years of age. Such a difference of 1.4% in unprotected sex has been shown to have a meaningful impact on pregnancy rates.[14, 50-52] The between-group difference in the incidence of unprotected sex of 1.3% (95% CI 0.5 to 2.2%) by nine months in our feasibility trial[45] demonstrates that such an effect size is plausible and is consistent with effect sizes seen in the literature.[50] The study will take account of clustering. In the feasibility data, the intraclass correlation coefficient (ICC) was 0.01.[45] As pilot studies can provide imprecise estimates of ICCs,[53] we re-estimated using ICCs from three sources, the RIPPLE cRCT,[52] a 2013/2014 WHO Health Behaviour in School-aged Children survey [25] and a 2013 Young Persons' Behaviour and Attitudes Survey in NI conducted by the Northern Ireland Statistics & Research Agency (NISRA).[26] The data from the WHO and NISRA studies were combined. The RIPPLE and combined WHO and NISRA studies found an ICC of 0.004. Assuming 120 students per school, an ICC of 0.01 and 7% attrition (plus two additional schools to be conservative), a trial involving 33 schools per group will provide 80% power at a 5% significance level (a pupil participant sample size of n=7904, with n=224 reporting unprotected sex). The power would rise to 93% if the ICC is 0.004.

Recruitment and retention

Recruitment of schools

In each country, eligible schools will be stratified based on the percentage of students eligible for free school meals (%FSM) for the 2018/2019 academic year (schools above and below the median national percentage free school meals) as a proxy for the level of deprivation. In NI, 14 schools will be randomly selected from the above-median stratum and 10 from the below-median stratum (total 24). In England, Scotland and Wales, eight schools will be randomly selected from the above-median stratum and six from the below-median stratum (to give a total of 14). The decision to select slightly

For peer review only - http://bmjopen.bmj.com/site/about/guidelines.xhtml

more schools from the above-median %FSM reflects research which indicates that teenage pregnancy and unprotected sex is more acute in more deprived areas.[1, 25]

The school recruitment period will run from February to June 2018. The school recruitment strategy is represented in Figure 2. In Scotland, it is required that permission is given from each local authority (typically by approaching the Director of Education) prior to commencing recruitment. Any schools that decline to participate will be replaced by a randomly selected school in the same stratum.

Recruitment of teachers

Once a school has made a decision to participate, a member of the research team will meet with teachers (identified by a school-assigned 'Trial Champion') responsible for the delivery of RSE to the target pupil year groups during the 2018/2019 academic year, to deliver an information session and answer any outstanding enquiries. Teachers will be provided with a copy of the school letter, information sheet, memorandum of understanding and consent form.

Recruitment of pupils

When a school and the relevant teachers who will be delivering the intervention to pupils have provided consent, the first step taken towards pupil recruitment is to inform parents/guardians. Schools (with the assistance of the school administrator) will be asked to post a hard copy of the parents'/guardians' information sheet and an opt-out consent form with pre-paid response envelopes. Schools will be responsible for addressing and preparing envelopes for postage. Parents/guardians will be advised within the material provided that they have to return the opt-out consent forms by a date no later than three weeks prior to commencement of baseline data collection within the school. The trial co-ordinator will collate a list of parents/guardians who have opted their child out of participation and return this to participating teachers.

For peer review only - http://bmjopen.bmj.com/site/about/guidelines.xhtml

Schools will be provided with printed copies of the pupil information sheets to be distributed to eligible pupils at least one week prior to baseline data collection. Only eligible pupils whose parents/guardians did not opt-out of providing consent for them to participate will be provided with a copy of the pupil information sheet. Immediately prior to administering the baseline questionnaire, eligible pupils will attend a short information session, delivered by a member of the research team, including an information video. Pupils will be given an opportunity to ask questions prior to deciding whether to participate. A repeat information session and baseline data collection session will be facilitated in agreement with the school to accommodate any pupils who are absent from the initial session. In the unlikely event that absenteeism remains in excess of 5-10% in a school, the research team, in agreement with the school, will return a third time to facilitate an additional information session and baseline data collection. Pupils with mild learning difficulties or poor English will be supported where possible by fieldworkers to complete the questionnaires.

Retention

To promote school, teacher and pupil retention and complete follow-up, schools will be provided with £1000 upon completion of baseline and follow up measures. Trial co-ordinators will be proactive in resolving any issues that arise with schools. Periodic communications will be provided by trial co-ordinators to inform schools, staff, pupils and parents (depending on preferences of schools) of the current status of the study, and plans for the next phase, as well as to acknowledge their support.

Randomisation

Following baseline data collection, randomisation will be carried out by the NI Clinical Trials Unit (NICTU, a UK Clinical Research Collaboration registered CTU), who will produce eight randomisation schedules (using unique identifiers for schools), one for each %FSM stratum within each country, using random permuted blocks of mixed size, generated using nQuery Advisor 7.0. The NICTU are not involved with recruitment and will only release the randomisation code (in sealed envelopes)

For peer review only - http://bmjopen.bmj.com/site/about/guidelines.xhtml

when all schools have been recruited and baseline data collection completed, therefore allocation concealment will be ensured.

Intervention

The intervention will be described in accordance with the Template for Intervention Description and Replication (TIDieR) guidelines.[87]

Name and brief description: If I Were Jack is an evidenced-based RSE teacher-delivered intervention designed to prevent unintended pregnancy and promote positive sexual health by increasing teenagers' intentions to avoid teenage pregnancy through delaying sexual intercourse or using contraception consistently. It is especially designed to engage with males but can be delivered to both male and female pupils. The underpinning theoretical framework for this intervention combines the well-established Theory of Planned Behaviour [57] and critiques to this theory[58] which focus on the inclusion of an understanding of the broader socio-environmental factors (such as socio-economic status; SES) and underlying values (such as religiosity and gender ideologies) associated with the occurrence of teenage pregnancy.[14, 59] The *If I Were Jack* Theory of Change Logic Model is depicted in Figure 3.

Why, rationale of essential elements: If I Were Jack targets six psycho-social mechanisms which research indicates are related to a reduction in risk-taking behaviour: knowledge, communication skills, attitudes, social influences, beliefs about capabilities, and intentions (Table 1).[15, 60, 61]

Table 1: Psychosocial and behavioural components of the *If I Were Jack* intervention

Component	Aim
Knowledge	Increase knowledge of: ways of avoiding unintended pregnancy; roles and responsibilities of young men in relation to unintended pregnancy; possible negative relational, social, emotional and financial consequences of unintended pregnancy; and sources of information and support for unintended pregnancy and sexual health more broadly

For peer review only - http://bmjopen.bmj.com/site/about/guidelines.xhtml

Communication skills	Increase skills in communicating with parents, peers and sexual partners about avoiding unintended pregnancy
Attitudes	Increase anticipated regret about the consequences of unintended pregnancy on current and future goals
Social influences	Increase awareness of peer norms, stereotypical gender norms and parental attitudes and beliefs about teenage pregnancy<br>Gender norms: increase perception that both men and women have roles and responsibilities in avoiding and dealing with the consequences of unintended pregnancy<br>Peer norms: increase perception that most peers are not sexually active and use contraception when they are<br>Parental values and beliefs: increase awareness of parental attitudes and beliefs about unintended pregnancy
Beliefs about capabilities	Increase perceived behavioural control to avoid unintended pregnancy (say no to sex or obtain and use contraception correctly) and increase self-efficacy to communicate about avoiding unintended pregnancy with parents, peers and professionals
Intentions	Increase strength of intention to avoid unplanned teenage pregnancy

For peer review only

The intervention components provide pupils with educational information and opportunities for discussion, skills practice, reflection and anticipatory thinking and are designed to specifically target one or more of the above psycho-social mechanisms.[62] The intervention components also include explicit reference to the impact of SES, religion and gender norms on sexual behaviour, inviting participants to think through how underlying social influences, such as social class and gender norms of sexual behaviour, can be challenged through individual agency. A feasibility trial demonstrated the acceptability to teachers and pupils and feasibility of implementation across a wide range of schools in Northern Ireland.[45]

What, a description of the materials: The *If I Were Jack* intervention consists of the following elements:

For peer review only - http://bmjopen.bmj.com/site/about/guidelines.xhtml

- A culturally sensitive interactive video drama (IVD) intended to immerse teenagers in a story of a week in the life of Jack, a teenager who has just been told by his girlfriend that she is pregnant. By asking males and females to imagine they were Jack and how they would think and feel if they were in his situation, it is designed to expose and challenge the gender assumptions around roles and responsibilities for teenage pregnancy by opening them up for reflection and negotiation. Informed by the findings of a prior transferability study that followed the feasibility trial of the intervention,[45] two versions of the IVD have been made available: one for use in England and Wales, using actors with English accents and one for use in NI and Scotland, using actors with NI accents. The IVD is designed to be delivered on individual computers/tablets with the use of headphones.
- Classroom materials for teachers containing detailed lesson plans with specific classroom-based and homework activities designed to build pupils' skills to a) obtain relevant sexual health information and b) develop communication skills with peers and trusted adults.
- A standardised 60-minute training session for RSE teachers implementing the intervention. The training session will adhere to a pre-defined teacher-trainer protocol and will be delivered in schools by country-specific established statutory and non-statutory RSE co-ordinators who normally provide RSE teacher training in schools.
- Two short animated films to engage parents/guardians and help/encourage them to have a conversation with their teenager about avoiding unintended pregnancy. A link to the web-hosted films will be texted and/or emailed via a school administrator to all parents/guardians of participating pupils in intervention schools (with one additional reminder text/email).
- A dedicated website (www.qub.ac.uk/IfIWereJack) for the intervention, will act as a portal of dissemination, providing password protected access to the intervention materials that teacher-trainers, teachers, parents, and pupils can access.

Who delivers the intervention? It is designed to be delivered by trained RSE teachers.

For peer review only - http://bmjopen.bmj.com/site/about/guidelines.xhtml

How, modes of delivery: *If I Were Jack* can be delivered either over four 50-60 minute lessons or over six 35-45 minute lessons and consists of a combination of classroom-based activities (mainly group discussion) having first viewed the IVD, in addition to pupils being asked to engage in two homework activities (one of which involves discussion with parents/guardians). Adherence to the intervention protocol will be determined as part of our process evaluation.

Where, locations where intervention has occurred: The intervention has been delivered in NI[45] and Ireland, using a further locally produced IVD for Ireland. A version of the intervention, the IVD only, has been delivered in schools in South Australia.

Outcomes

Primary outcome

In this trial, a reduction in unintended teenage pregnancy rates would be the ideal primary outcome measure, but the sample size would need to be very large to detect change in unintended pregnancy rates. We will therefore use a surrogate measure associated with unintended teenage pregnancy: unprotected sex at last sexual encounter, as defined by sexual intercourse without use of contraception (barrier or hormonal). Unprotected sex during teenage years is well established as the main proximate behavioural determinant of teenage pregnancy and is a commonly measured behavioural outcome in studies examining the impact of RSE interventions on teenage pregnancy.[10, 12, 14, 50, 63, 64] Studies indicate that, although other behavioural determinants (such as frequency of sexual intercourse and number of sexual partners) are important, avoidance of unprotected sex via consistent use of contraception is central in explaining variation in levels of teenage pregnancy.[18, 51] The primary outcome will be based on contraception use at last sexual intercourse of unprotected sex at follow-up (i.e. answers to the question "Did you use any form of contraception the last time you had sex?"), consistent with the data on which the sample size calculation was based.[25] An additional item will also be included related to lifetime incidence of unprotected sex in order to account for sporadic use of contraception that may not be reflected in

For peer review only - http://bmjopen.bmj.com/site/about/guidelines.xhtml

the last sexual encounter. Participants reporting the use of natural family planning or withdrawal methods will be categorised as having engaged in unprotected sex due to the reduced efficacy of these methods in preventing pregnancy and transmission of sexually transmitted infections (STIs). This study will not undertake any data linkage with Health and Social Care or National Health Service records, given that data on conception rates are not available in NI and that data for sexual health related services in England are not readily available as part of routinely collected data given patient privacy requirements.

Secondary outcomes

Secondary outcomes are 12-month impacts on knowledge, attitudes, skills and intentions to avoiding teenage pregnancy. Secondary outcomes informed by our theory of change (see Figure 3) include knowledge, attitudes, communication skills and intentions relating to avoiding teenage pregnancy at follow-up and are hypothesized to lead to increased intention to avoid unprotected sex. Data will be collected using a number of standardized measures, including the male role attitudes scale,[69] sexual socialisation instrument,[70] sexual self-efficacy scale.[71] comfort communicating about pregnancy and comfort communicating about contraception derived from mathtech behaviour inventory.[68] We will also collect data using an 'Intentions to avoid a teenage pregnancy scale', developed and psychometrically tested in our feasibility trial.[45] The measures were selected because the constructs they measure map closely to the theoretical framework underpinning the intervention and the reliability and completion rates of the measures were satisfactory in the feasibility trial.[45] In addition, to assist with the economic evaluation, supplementary secondary outcomes include: frequency of engagement in sexual intercourse, contraception use, diagnosis of STIs and incidence of pregnancy and pregnancy outcomes. The collection of this data was also shown to be feasible in the feasibility trial. Finally, we will collect important individual level demographic and socio-economic characteristics of the sample to deepen understandings of how these factors moderate effectiveness.

For peer review only - http://bmjopen.bmj.com/site/about/guidelines.xhtml

For peer review only

Data collection

Participating pupils will be in the study for approximately 18 months and asked to complete a paper-based questionnaire during one RSE lesson at baseline and again between 12 and 14 months later. A fieldworker will administer questionnaires to pupils, under exam conditions, two weeks prior to commencement of intervention delivery. Informed by the process evaluation conducted in the feasibility study,[45] teachers will be asked to stay at the front of the classroom to maintain order while alleviating any concerns that teachers may be able to see pupils' answers. Additional fieldworkers will be available to provide support to pupils who require extra help and to ensure questionnaires are completed confidentially.

Primary outcome

Process evaluation

Informed by realist approaches to the evaluation of interventions,[44, 72, 73] the process evaluation has four aims. First, we will examine reasons for school participation and non-participation to inform risk of bias in the trial as well as longer term sustainability of implementation of the intervention. Second, we will examine intervention delivery and fidelity in the context of overall RSE provision in intervention schools. Third, we will assess provision in control schools and potential contamination caused by any changes to provision that could be due to participation in the trial. Fourth, we will explore self-reported perceptions of effectiveness and moderating influences in intervention schools among a sample of pupils, teachers and school principals and parents. Triangulated data collection methods will include semi-structured interviews with teachers, focus group discussions with pupils, observations of a sample of lessons and a survey of parents/guardians with follow-up focus groups. For detail on our approach to integration of the process evaluation with the experimental design methodology to achieve research objectives, see Figure 4.

All schools

For peer review only - http://bmjopen.bmj.com/site/about/guidelines.xhtml

The school-assigned trial champion or a suitable person identified by the trial champion will complete a school background questionnaire, designed to detail more general information pertinent to trial implementation i.e. school experience of teenage pregnancy, school holidays/closures, school experience of pupils/parents or guardians who do not speak English as a first language or who do not understand English at all, and school involvement in other research. An appropriate member of staff identified by the school-assigned trial champion will also complete a questionnaire about current RSE provision in the school to gain a better understanding of the nature, quantity and quality of RSE currently taught within the school as well as the facilitators and barriers to current RSE provision within the school. A school administrator will be asked to fill out a school administrator resource use record detailing associated costs (i.e. postage of parent/guardian information) and time spent. Schools will be reimbursed up to the value of £100 for these costs.

Intervention schools

Parent/guardian online survey: Parents/guardians will have been made aware when they were in receipt of the parents'/guardians' information sheet that there would be an opportunity for them to respond to a short online parent/guardian survey. Eligible parents/guardians (who have not opted out of providing consent for their child to participate) will receive the link to this survey (hosted using the SurveyMonkey® UK platform) in an email and/or text message issued by the school administrator inviting them to participate (post intervention delivery). The survey will ask parents/guardians about their engagement with and opinion of the parent/guardian animations and homework session and whether their child has discussed with them their experience of engaging with *If I Were Jack*. The questionnaire will be translated where possible and where necessary.

Teacher implementation log: Teachers responsible for delivering the intervention will be asked to complete an implementation log to detail what activities were completed or not completed during each lesson, and perception of pupil engagement with each activity.

For peer review only - http://bmjopen.bmj.com/site/about/guidelines.xhtml

Telephone interviews: 15-30 minute telephone interviews will be conducted by trial co-ordinators with school principals or trial champions in intervention schools that are not ‘case study schools’ (see below) to determine any barriers or facilitators of engagement with the intervention.

Case study schools

Participating intervention schools will be randomly rank ordered in each country and two case study schools from each country will be randomly selected by NICTU to participate in the process evaluation. Should a school refuse participation, a further random selection will be made.

Observations: Country-specific trial co-ordinators will conduct structured observations of one randomly selected lesson in every class group in receipt of the intervention in the eight case study schools. Observations will be focused primarily on measuring teacher fidelity to implementation protocol and pupil engagement.

Focus groups: Trial co-ordinators will conduct three 60-minute focus group discussions in each of the eight case study schools. One group will be composed of all teachers who delivered the intervention. The second group will include a maximum of six English-speaking pupils who received the intervention. Teachers who delivered the intervention will ask for a mixture of male and female pupil volunteers and pass details of those pupils to the trial co-ordinator. In the event that more pupils volunteer than are needed (per school), a random selection will be made. The third group will be a maximum of six English-speaking parents/guardians (of children who received the intervention). Discussions will focus on perceived barriers and facilitators of successful implementation and engagement with different components of the intervention.

Fieldworkers

Fieldworkers will complete a fieldworker perception form after each visit to a school, asking them to detail what worked well and what did not in relation to data collection and any other relevant observations they may have.

For peer review only - http://bmjopen.bmj.com/site/about/guidelines.xhtml

Education/policy specialists

Trial co-ordinators will conduct telephone or face-to-face interviews with one or two education/policy specialists in each country. Interviews will focus on the current context of RSE policy and perceptions of how this might influence the uptake of the *If I Were Jack* intervention.

Economic evaluation

The economic evaluation will aim to describe the costs and consequences of implementing *If I Were Jack* in UK schools so as to provide information to decision makers on the implications of potential further roll-out of the intervention. This will include the duration of time taken up by *If I Were Jack* in school from the perspective of the teacher and impact on time spent on other important curricula activities compared to time spent on standard RSE. The aim of this will be to provide a measure of the opportunity cost to schools of implementing *If I Were Jack* compared to current RSE. The structure of the evaluation will follow NICE guidance for evaluating public health interventions[74] and recent guidance published by Edwards et al[75] on economic evaluations in public health. Costs will include the cost of implementing the intervention in schools including any training involved and the cost of current RSE in the control schools. We will also collect information on healthcare cost information in the intervention and control arms including the costs of sexual health related primary care attendances, costs associated with STIs and unintended pregnancies (although numbers of these are likely to be small). The cost of adapting *If I Were Jack* to different groups will also be reported given that others may want to adapt the intervention before rolling it out. Mean cost per pupil will be reported alongside consequences including use of contraception, STIs and unintended pregnancies collected using questionnaires administered to pupils at baseline and follow-up. Although 12-14 month recall time is a relatively long time-period, pupils are likely to be able to recall high impact events that occurred during this period. The follow-up time is also important to fit within the school year timetable. Costs will also be reported by country, given the different sexual health services provided and hence differential implications for health service costs by country.

For peer review only - http://bmjopen.bmj.com/site/about/guidelines.xhtml

Given that STIs and unintended pregnancies are likely to be rare but potentially high impact events in this population group, the long-term costs and consequences will be modelled as part of the cost-effectiveness decision model (Figure 5), incorporating theories of behaviour change and identified as applicable for use in this trial during the feasibility trial.[45] In addition to collecting information as part of the trial, we will look to systematic reviews of evidence of the impact of digital interventions on sexual health behaviour in this population group, for example the review recently undertaken and published by Bailey et al.[76] We will undertake one way, two way and probabilistic sensitivity analyses of the results. Cost effectiveness acceptability curves and cost-effectiveness planes will be reported. The model will have a 20-year time horizon and discounting of future costs and benefits will comply with NICE guidance for evaluating public health interventions.[74]

Statistical Methods

The reporting and presentation of findings will be in accordance with the CONSORT guidelines for cRCTs.[77] All analyses will take account of clustering by school using robust standard errors, and intervention and control groups will be compared at baseline via frequencies/descriptive statistics (percentage, mean or median as appropriate) in relation to sex, ethnicity, SES at school level (using percentage free school meals and post-code data) and at individual level (using highest education qualifications of parents), primary and secondary outcomes.

Primary analysis (12-14 month follow-up): The primary effectiveness analysis will be on an intention to treat basis, using a multi-level logistic regression model (two levels: pupils nested within schools) adjusting for the baseline outcome and stratification variables.[78] Sensitivity analyses, making different assumptions on the best and worst case scenarios, as well as imputation models of missingness will be conducted to investigate the potential impact of missing data.

Secondary analysis (12-14 month follow-up): Although the trial is not powered to detect the influence of mediating and moderating variables, we will examine the following outcomes informed by our theory of change model (see Figure 3): i) interaction terms will be used to investigate possible

For peer review only - http://bmjopen.bmj.com/site/about/guidelines.xhtml

differences in the effect of the intervention on the primary outcome by whether pupils at baseline reported having had unprotected sex or not, country (Wales, England, Scotland, NI), sex, socio-economic group (see earlier section 5.5) and ethnicity); ii) a mediational analysis, using an analytic framework recommended for RCTs,[79] will be used to explore whether the effect of the intervention on the primary outcome is mediated by individual-level sexual health knowledge and sexual competence, perceived behavioural control, intentions to avoid an unintended pregnancy, communication with parents, and gender ideologies. In these secondary analyses, p-values will be interpreted with caution due to the low power and number of interactions being tested (e.g. use of Bonferroni corrected p-values).

Process evaluation

All data will be transcribed verbatim (in the case of interviews) or written up in detail (in the case of observational field notes and other secondary source data). These data will be organised using 'NVivo' software and analysed systematically and thematically based on the six steps proposed by Braun and Clarke[80] to enable identification and analysis of patterns (or 'themes') within the data by moving iteratively between theoretical understandings and the new data. These inductively and deductively derived codes will be analysed to form overarching themes emerging from each of the participant groups outlined above. We will use qualitative software 'NVivo 10' to organise the data, and we will ensure methodological rigour by establishing credibility, transferability, dependability and confirmability using techniques suggested by Lincoln and Guba.[81] In addition, following Hyde et al,[82] specific attention will be given to analysing the group dynamics of the focus groups as part of the overall interpretive process.

DISCUSSION

The strengths of this study include that this is the first RSE intervention to be developed and trialled which explicitly promotes a gender-sensitive approach to addressing teenage pregnancy by focussing on male perspectives. The intervention is culturally sensitive to different parts of the UK, is

For peer review only - http://bmjopen.bmj.com/site/about/guidelines.xhtml

non-directive in terms of pregnancy resolution options and is sufficiently flexible to be taught within the framework of a school's ethos and personal development/RSE policy, including in faith-based schools. This has particular significance in NI, with almost half (46%) of all NI grammar and secondary schools identifying as a Roman-Catholic school.[83] This is also the first RSE intervention to be developed and trialled across all four nations of the UK, allowing for exploration of what works best where. An additional strength of the study is that the embedded process evaluation involves triangulation of sources including school management, teachers, pupils, parents and RSE statutory and voluntary stakeholders. Study limitations include that the pragmatic setting – within schools – means that schools and participating teachers, pupils and parents will remain unblinded to the allocation. Finally, the use of the surrogate measure of unprotected sex rather than a biological measure (such as conception rates) introduces the possibility that the findings of the trial will be influenced by self-report bias, but the veracity of this measure is enhanced by privacy, confidentiality and a control group.

ETHICS AND DISSEMINATION

In writing this protocol, we have endeavoured to adhere to the recommendations and guidance provided in the Consolidated Standards of Reporting Trials (CONSORT) 2010 statement and the extension for cRCTs,[77, 84] the Standard Protocol Items: Recommendations for Intervention Trials (SPIRIT) Statement 2013[85, 86] and the Template for Intervention Description and Replication (TIDieR).[87] When registering the trial, we have provided structured summary information in accordance with the requirements of the WHO Trial Registration Data Set (Supplementary File 3).[88]

Any modifications to the protocol which may impact on the conduct of the study, potential effectiveness, or impact study participants, including changes of study objectives, study design, sample size, study procedures or significant administrative aspects will require a formal amendment to the protocol. Such amendment will be agreed upon by the JACK Trial Steering Committee,

For peer review only - http://bmjopen.bmj.com/site/about/guidelines.xhtml

reported to the funder (National Institute of Health Research, NIHR) and approved by the ethics committee prior to implementation. Minor administrative changes, corrections or clarifications that have no effect on the way the study is to be conducted will be agreed upon by the JACK Trial Steering Committee, documented and reported to the NIHR. The ethics committee may be notified of such changes at the discretion of the JACK Trial Steering Committee.

A full study report will be submitted to the NIHR by the end of December 2020 and made publically available thereafter in the Public Health Research journal on the NIHR Journals Library. We shall make data available to the scientific community with as few restrictions as feasible, following receipt of a request to the corresponding author, while retaining exclusive use until the publication of major outputs via academic conference presentations and journal articles in addition to material created for relevant stakeholders. Pupil friendly brief reports will be provided to all participating schools.

Ethical approval for the JACK Trial was granted by the Research Ethics Committee of the School of Nursing and Midwifery, Queen's University Belfast in July 2017 (Ref: 11.MLohan.05.17.M6.V1).

Safety and data monitoring

This is a low-risk study, therefore, a Data Monitoring Committee is not required and no interim analysis is planned.

ACKNOWLEDGEMENTS

In addition to the authors listed, the JACK Trial study thus far has been dependent on the commitment provided by extensive public involvement. The intervention has been designed, developed and piloted in Ireland, NI and South Australia involving over ten years of research with pupils, teachers, sex education specialists, and education and health promotion departments. These collaborations have influenced the study by allowing different perspectives to inform the design and optimal conditions of implementation. We specifically acknowledge the role of the Trial Steering Committee, comprised of trials experts, a school principal, a school teacher, parent and pupils, who

For peer review only - http://bmjopen.bmj.com/site/about/guidelines.xhtml

have provided independent expert advice on the intervention itself as well as refining research methods for delivery in schools. We also wish to acknowledge our Stakeholders Group, composed of a UK-wide group of RSE specialists and senior representatives from key statutory organisations and government departments who have helped to refine the intervention and the study design by ensuring the research outcomes are important to public concerns and the methods proposed are acceptable and sensitive to all study participants. We would also like to acknowledge the invaluable involvement of our Young People's Advisory Groups (one in each country in the UK) who have enabled to us to further refine the intervention in a culturally sensitive way for effectiveness testing in the whole of the UK. Finally, we thank all schools, teachers, parents and pupils who have participated in the feasibility, transferability and pilot studies that have preceded this trial.

FUNDING STATEMENT

This research is funded by the National Institute for Health Research (NIHR) Public Health Research programme, grant number NIHR PHR 15/181/01. LMc is funded by the UK Medical Research Council (MRC) and Scottish Government Chief Scientist Office (CSO) at the MRC/CSO Social & Public Health Sciences Unit, University of Glasgow (MC_UU_12017/11, SPHSU11). The views expressed are those of the authors and not necessarily those of the NHS, the NIHR or the Department of Health. This funding source had no role in the design of this study and will not have any role during its execution, analyses, interpretation of the data, or decision to submit results.

COMPETING INTERESTS STATEMENT

QUB holds copyright and the researchers do not benefit financially from its evaluation or use. We report no competing interests or conflicts of interest.

TRIAL STATUS

At the time of submission of this protocol (January 2018) optimisation of the intervention is underway, but no aspect of the trial has begun yet.

For peer review only - http://bmjopen.bmj.com/site/about/guidelines.xhtml

AUTHORS' CONTRIBUTIONS

ML, AA, MC and LM conceived of the study and led study design. RC and ML drafted this manuscript. All authors made substantive contributions to the development of the protocol, critically reviewed and gave final approval to the manuscript. AA is responsible for management of the trial and process evaluation supported by ML. HY, AF, LMc, CB, RF, LO'H and JB contributed to the design of study and development of trial recruitment areas and PPI involvement. HY and AF led on young people's advisory group. CM and JY contributed to sample size calculation and statistical analysis plan. RH developed the economic evaluation. LO'H contributed to knowledge translation and dissemination plan.

REFERENCES

1. Harden A, Brunton G, Fletcher A, et al. Teenage pregnancy and social disadvantage: systematic review integrating controlled trials and qualitative studies. *BMJ* 2009;339:b4254 doi: 10.1136/bmj.b4254 [published Online First: 13 November 2009].

2. Office of National Statistics (2014) International comparisons of teenage births 2014. http://web.ons.gov.uk/ons/rel/vsob1/births-by-area-of-usual-residence-of-mother--england-and-wales/2012/sty-international-comparisons-of-teenage-pregnancy.html (Accessed May 20th 2016)

3. Office of National Statistics. Dataset: 2015 Conception Statistics, England and Wales https://www.ons.gov.uk/peoplepopulationandcommunity/birthsdeathsandmarriages/conceptionandfertilityrates/datasets/conceptionstatisticsenglandandwalesreferencetables [Accessed October 2017].

4. NHS Scotland Information Services Division. Teenage Pregnancy Year of conception ending 31 December 2015. https://www.isdscotland.org/Health-Topics/Sexual-Health/Publications/2017-07-04/2017-07-04-TeenPreg-Summary.pdf [published 04 July 2017, Accessed October 2017].

For peer review only - http://bmjopen.bmj.com/site/about/guidelines.xhtml

5. NISRA. Births (administrative geographies) 1999-2014. Northern Ireland Statistics & Research Agency (NISRA); 2015.

6. Department of Health Social Services and Public Safety. NI health and social care inequalities monitoring system (HSCIMS) - Health inequalities in Northern Ireland: key facts 2015; https://www.health-ni.gov.uk/sites/default/files/publications/dhssps/hscims-2015-key-facts.pdf [published 2015, Accessed September 2017].

7. Bonell C. Why is teenage pregnancy conceptualized as a social problem? A review of quantitative research from the USA and UK. *Cult Health Sex* 2004;6:255–72 doi: 10.1080/13691050310001643025 [published Online First: 6 October 2011].

8. Beers LAS, Hollo RE. Approaching the adolescent-headed family: a review of teen parenting. *Curr Probl Pediatr Adolesc Health Care* 2009;39:216–33 doi: 10.1016/j.cppeds.2009.09.001 [published Online First 24 October 2009].

9. Francesconi M. Adult outcomes for children of teenage mothers. *Scand J Econ* 2008;110:93–117 doi: 10.1111/j.1467-9442.2008.00526.x [published Online First March 2008].

10. Wight D, Raab GM, Henderson M, et al. Limits of teacher delivered sex education: interim behavioural outcomes from randomised trial. *BMJ* 2002;324:1430 doi: 10.1136/bmj.324.7351.1430 [published Online First 15 June 2002].

11. Silva M. The effectiveness of school-based sex education programs in the promotion of abstinent behavior: a meta-analysis. *Health Educ Res* 2002;17:471–81 doi: 10.1093/her/17.4.471 [published Online First 01 August 2002].

12. Stephenson JM, Oakley A, Johnson AM, et al. A school-based randomized controlled trial of peer-led sex education in England. *Control Clin Trials* 2003;24:643–57 doi: 10.1016/S0197-2456(03)00070-9 [published Online First 13 September 2003].

For peer review only - http://bmjopen.bmj.com/site/about/guidelines.xhtml

13. Fullerton D. Promoting Positive Adolescent Sexual Health & Preventing Teenage Pregnancy-of Recent Effectiveness Research. Dublin: Crisis Pregnancy Programme http://crisispregnancy.ie/wp-content/uploads/2012/05/Rep22.-promoting-positive-adolescent-sexual-health-and-preventing-teenage-pregnancy-a-review-of-recent-effectiveness-research.pdf [published 2004, Accessed September 2017].

14. Henderson M, Wight D, Raab GM, et al. Impact of a theoretically based sex education programme (SHARE) delivered by teachers on NHS registered conceptions and terminations: final results of cluster randomised trial. *BMJ* 2007;334:133 doi: 10.1136/bmj.39014.503692.55 [published Online First 18 January 2007].

15. Shepherd J, Kavanagh J, Picot J, et al. The effectiveness and cost-effectiveness of behavioural interventions for the prevention of sexually transmitted infections in young people aged 13-19: a systematic review and economic evaluation. *Health Technol Assess* 2010;14:1–206 doi: 10.3310/hta14070 [published Online First February 2010].

16. Elliott L, Henderson M, Nixon C, et al. Has untargeted sexual health promotion for young people reached its limit? A quasi-experimental study. *J Epidemiol Community Health* 2013;67:398–404 doi: 10.1136/jech-2012-201034 [published Online First 20 February 2013].

17. Downing J, Jones L, Cook P, et al. Prevention of sexually transmitted infections (STIs): a review of reviews into the effectiveness of non-clinical interventions: Evidence Briefing Update https://www.nice.org.uk/guidance/ph3/evidence/evidence-briefing-update-prevention-of-sexually-transmitted-infections-stis-2006-65843250 [published 2006, Accessed September 2017].

18. Swann C, Bowe K, McCormick G, et al. Teenage pregnancy and parenthood: a review of reviews. Evidence briefing for Health Development Agency. London: Health Development Agency; 2003.

For peer review only - http://bmjopen.bmj.com/site/about/guidelines.xhtml

19. Ellis S, Grey A. Prevention of Sexually Transmitted Infections (STIs): a review of reviews into the effectiveness of non-clinical interventions to reduce the risk of sexual transmission. Evidence Briefing. London: Health Development Agency; 2004.

20. Ingham R, Hirst J. Promoting sexual health. In: Dennison C, Warwick I, editors. Promoting Health and Wellbeing in Schools. London: Routledge; 2010.

21. Oringanje C, Meremikwu MM, Eko H, et al. Interventions for preventing unintended pregnancies among adolescents. *Cochrane Database Syst Rev* 2009;3;2:CD005215 doi: 10.1002/14651858.CD005215.pub3 [published Online First 3 February 2016].

22. National Institute for Health and Care Excellence. Sexually transmitted infections and under-18 conceptions: prevention; 2007.

23. Committee for Health, Social Services and Public Safety, Northern Ireland Assembly. Official Report (Hansard) Sexual Health Promotion Strategy and Action Plan 2008-2013 http://www.niassembly.gov.uk/globalassets/documents/official-reports/health/2011-2012/sexual-health-promotion-strategy-and-action-plan-2008-2013.pdf [published 18 April 2012, Accessed September 2017].

24. The Scottish Government. Pregnancy and Parenthood in Young People Strategy 2016-2026 https://www.scottishwomensconvention.org/content/outcomes-scotland-2016/Pregnancy-and-Parenthood.pdf [published March 2016, Accessed September 2017].

25. WHO, 2016. Growing up unequal: gender and socioeconomic differences in young people's health and well-being. Health Behaviour in School-aged Children (HBSC) study: international report from the 2013/2014 survey. Health Policy for Children and Adolescents, No. 7; 2016 http://www.euro.who.int/__data/assets/pdf_file/0003/303438/HSBC-No.7-Growing-up-unequal-Full-Report.pdf?ua=1 [Accessed September 2017].

For peer review only - http://bmjopen.bmj.com/site/about/guidelines.xhtml

26. Northern Ireland Statistics and Research Agency. Central Survey Unit. (2015). Young Persons' Behaviour and Attitudes Survey, 2013. [data collection]. UK Data Service. SN: 7624, http://doi.org/10.5255/UKDA-SN-7624-1

27. Kirby D, Laris B, Rolleri L. Sex and HIV education programs for youth: Their impact and important characteristics http://recapp.etr.org/recapp/documents/programs/SexHIVedProgs.pdf [published 13 May 2006, Accessed September 2017].

28. Kirby D. Effective approaches to reducing adolescent unprotected sex, pregnancy, and childbearing. *J Sex Res* 2002;39:51–7 doi: 10.1080/00224490209552120 [published Online First 11 January 2010].

29. Kirby D. Emerging Answers 2007: Research findings on programs to reduce teen pregnancy and sexually transmitted diseases. Washington DC: National Campaign to Prevent Teen and Unplanned Pregnancy http://www.siecus.org/index.cfm?fuseaction=Feature.showFeature&featureID=1151 [published November 2007, Accessed September 2017].

30. Robin L, Dittus P, Whitaker D, et al. Behavioral interventions to reduce incidence of HIV, STD, and pregnancy among adolescents: a decade in review. *J Adolesc Health* 2004;34:3–26 doi: 10.1016/S1054-139X(03)00244-1 [published Online First 29 December 2003].

31. Bailey JV, Murray E, Rait G, et al. Interactive computer-based interventions for sexual health promotion. *Cochrane Database Syst Rev* 2010;9:CD006483 doi: 10.1002/14651858.CD006483.pub2 [published Online First 8 September 2010].

32. Guse K, Levine D, Martins S, et al. Interventions using new digital media to improve adolescent sexual health: A systematic review. *J Adolesc Health* 2012;51:535–43 doi: 10.1016/j.jadohealth.2012.03.014 [published First Online 4 May 2012].

For peer review only - http://bmjopen.bmj.com/site/about/guidelines.xhtml

33. Noar SM, Pierce LB, Black HG. Can Computer-Mediated Interventions Change Theoretical Mediators of Safer Sex? A Meta-Analysis. *Hum Commun Res* 2010;36:261–97 doi: 10.1111/j.1468-2958.2010.01376.x [published Online First 7 June 2010].

34. Lohan M, Cruise S, O'Halloran P, et al. Adolescent men's attitudes and decision-making in relation to an unplanned pregnancy. Responses to an interactive video drama. *Soc Sci Med* 2011;72:1507–14 doi: 10.1016/j.socscimed.2011.02.044 [published Online First 24 March 2011].

35. Smith LH, Guthrie BJ, Oakley DJ. Studying adolescent male sexuality: Where are we? *J Youth Adolesc* 2005;34:361–77 doi: 10.1007/s10964-005-5762-5

36. Guttmacher Institute. In their own right: Addressing the sexual and reproductive health needs of American men https://www.guttmacher.org/report/their-own-right-addressing-sexual-and-reproductive-health-needs-american-men [published October 2003, Accessed September 2017].

37. Marsiglio W, Ries A, Sonenstein F, et al. It's a Guy Thing: Boys, Young Men, and Teen Pregnancy Prevention. The National Campaign to Prevent Teen Pregnancy https://thenationalcampaign.org/resource/its-guy-thing [published February 2006, Accessed September 2017].

38. Lindberg LD, Sonfield A, Gemmill A. Reassessing adolescent male sexual and reproductive health in the United States: research and recommendations. *Am J Mens Health* 2008;2:40–56 doi: 10.1177/1557988307309460 [published Online First 5 December 2007].

39. WHO. Evidence for gender responsive actions to prevent and manage adolescent pregnancy http://www.euro.who.int/__data/assets/pdf_file/0008/158093/316637_WHO_brochure_226x226_5-AdolecentPregnancy.pdf [published 2011, Accessed September 2017].

40. WHO. Health 2020: a European policy framework supporting action across government and society for health and well-being http://www.euro.who.int/__data/assets/pdf_file/0006/199536/Health2020-Short.pdf?ua=1

For peer review only - http://bmjopen.bmj.com/site/about/guidelines.xhtml

[published 2013, Accessed Setember 2017].

41. WHO. Health policy framework supporting action across government and society for health and well-being. 2012.

42. Ries A, Sonenstein F. What works for boys: A re-examination of coeducational school curricula. In: Troccoli K, editor. It's a guy thing boys young men and teen pregnancy prevention. Washington DC: National Campaign to Prevent Teen Pregnancy 2006:101–38.

43. Bennett SE, Assefi NP. School-based teenage pregnancy prevention programs: a systematic review of randomized controlled trials. *J Adolesc Health* 2005;36:72–81 doi: https://doi.org/10.1016/j.jadohealth.2003.11.097 [published Online First 18 January 2005].

44. Craig P, Dieppe P, Macintyre S, et al. Developing and evaluating complex interventions: the new Medical Research Council guidance. *BMJ* 2008;337:a1655 doi: 10.1136/bmj.a1655 [published Online First 29 September 2008].

45. Lohan M, Aventin Á, Maguire L, et al. Increasing boys' and girls' intention to avoid teenage pregnancy: a cluster randomised control feasibility trial of an interactive video drama based intervention in post-primary schools in Northern Ireland. *Public Health Res* 2017;5 doi: 10.3310/phr05010 [published Online First March 2017].

46. Crawford C, Cribb J, Kelly E. Teenage Pregnancy in England. Centre for the Analysis of Youth Transitions https://www.ifs.org.uk/publications/6702 [published 21 May 2013, Accessed September 2017].

47. Jones L, Bates G, Downing J, et al. A review of the effectiveness and cost-effectiveness of personal social and health education in secondary schools focussing on sex and relationships and alcohol education for young people aged 11-19 years. Final Report https://www.nice.org.uk/guidance/gid-phg0/documents/pshe-evidence-review-secondary-education-and-fe2 [published October 2009, Accessed September 2017].

For peer review only - http://bmjopen.bmj.com/site/about/guidelines.xhtml

48. Education and Training Inspectorate NI. Report of an evaluation of relationships and sexuality education in post-primary schools https://www.etini.gov.uk/publications/report-evaluation-relationships-and-sexuality-education-post-primary-schools [published 11 January 2011, Accessed September 2017].

49. Ofsted. Not yet good enough: personal, social, health and economic education in schools http://webarchive.nationalarchives.gov.uk/20141107025925/http://www.ofsted.gov.uk/resources/not-yet-good-enough-personal-social-health-and-economic-education-schools [published 01 May 2013, Accessed September 2017].

50. Chin HB, Sipe TA, Elder R, et al. The effectiveness of group-based comprehensive risk-reduction and abstinence education interventions to prevent or reduce the risk of adolescent pregnancy, human immunodeficiency virus, and sexually transmitted infections: two systematic reviews for the Guide to Community Preventive Services. *Am J Prev Med* 2012;42:272–94 doi: 10.1016/j.amepre.2011.11.006 [published Online First 14 February 2012].

51. Santelli JS, Lindberg LD, Finer LB, et al. Explaining recent declines in adolescent pregnancy in the United States: the contribution of abstinence and improved contraceptive use. *Am J Public Health* 2007;97:150–6 doi: 10.2105/AJPH.2006.089169 [published Online First 10 October 2011].

52. Parkes A, Wight D, Henderson M, et al. Contraceptive method at first sexual intercourse and subsequent pregnancy risk: findings from a secondary analysis of 16-year-old girls from the RIPPLE and SHARE studies. *J Adolesc Health* 2009;44:55–63 doi: 10.1016/j.jadohealth.2008.06.006 [published Online First 18 October 2008].

53. Eldridge SM, Costelloe CE, Kahan BC, et al. How big should the pilot study for my cluster randomised trial be? *Stat Methods Med Res* 2016;25:1039-56 doi: 10.1177/0962280215588242 [published First Online 12June 2015].

For peer review only - http://bmjopen.bmj.com/site/about/guidelines.xhtml

54. Aventin A, Lohan M, Maguire L, et al. Recruiting faith- and non-faith-based schools, adolescents and parents to a cluster randomised sexual-health trial: experiences, challenges and lessons from the mixed-methods Jack Feasibility Trial. *Trials* 2016;17:365 doi: 10.1186/s13063-016-1506-y [published Online First 29 July 2016].

55. Wight D, Abraham C, Scott S. Towards a psycho-social theoretical framework for sexual health promotion. *Health Educ Res* 1998;13:317–30 doi: 10.1093/her/13.3.317-a

56. NHS Health Scotland. An overview of the effectiveness of sexual health improvement interventions. Final Report. June 2010 http://www.healthscotland.com/uploads/documents/13786-REO28-OverviewEffectivenessSexualHealthInterventions.pdf [Accessed September 2017].

57. Ajzen I, Madden TJ. Prediction of goal-directed behavior: Attitudes, intentions, and perceived behavioral control. *J Exp Soc Psychol* 1986;22:453–74 doi: 10.1016/0022-1031(86)90045-4 [published Online First 27 August 2004].

58. Ajzen I. The theory of planned behaviour: reactions and reflections. *Psychol Health* 2011;26:1113–27 doi: 10.1080/08870446.2011.613995 [published Online First 20 September 2011].

59. Moore G, Evans R. What theory, for whom and in which context? Reflections on the application of theory in the development and evaluation of complex population health intervention. *SSM - Population Health* 2017;3:132-5 doi: 10.1016/j.ssmph.2016.12.005 [published Online First 13 December 2016].

60. Philliber S, Kaye JW, Herrling S, West E. Preventing pregnancy and improving health care access among teenagers: An evaluation of the Children's Aid Society-Carrera Program. *Perspect Sex Reprod Health* 2002;244–51 doi: 10.1363/3424402 [published Online First 1 September 2002].

61. Coyle K, Basen-Engquist K, Kirby D, et al. Safer choices: reducing teen pregnancy, HIV, and STDs. Public Health Rep 2001;116(Suppl 1):82–93 doi: 10.1093/phr/116.S1.82

For peer review only

62. Aventin Á, Lohan M, O'Halloran P, et al. Design and development of a film-based Intervention about teenage men and unintended pregnancy: Applying the Medical Research Council Framework in practice. *Eval Progr Plan* 2015;49:19–30 doi: 10.1016/j.evalprogplan.2014.11.003 [published Online First 15 November 2014].

63. NHS Health Scotland. Evaluation of Healthy Respect Phase Two: Final Report March 2010 http://www.healthscotland.com/uploads/documents/12159-HealthyRespectEvaluationReport_3342.pdf [Accessed September 2017].

64. Guttmacher Institute. Adolescent sexual and reproductive health in the United States https://www.guttmacher.org/fact-sheet/american-teens-sexual-and-reproductive-health [Accessed September 2017].

65. Carswell K, McCarthy O, Murray E, et al. Integrating psychological theory into the design of an online intervention for sexual health: The sexunzipped website. *JMIR Research Protocols* 2012;1 doi: 10.2196/resprot.2114 {published Online First 19 November 2012].

66. Elliott L, Henderson M, Wight D, et al. Evaluation of healthy respect phase two: Final report. 2010. Edinburgh: NHS Health Scotland.

67. Fullard W, Johnston D, Lief H. The sexual knowledge and attitude test for adolescents. In: Davis C, Yarber W, Bauserman R, Schreer G, Davis S, editors. Handbook of sexuality-related measures. London: SAGE; 2005. p. 33-35.

68. Kirby D. Sexuality Education: An evaluation of programs and their Effects. An executive summary. 1984. Washington DC: Network Publications.

69. Pleck JH, Sonenstein FL, Ku LC. Masculinity ideology: Its impact on adolescent males' heterosexual relationships. *J Soc Issues* 1993;49:1-29 doi: 10.1111/j.1540-4560.1993.tb01166.x [published Online First 14 April 2010].

For peer review only - http://bmjopen.bmj.com/site/about/guidelines.xhtml

70. Lottes I, Kuriloff P. Sexual socialization differences by gender, Greek membership, ethnicity and religious background. *Psychol Women Quar* 1994;18:203-19 doi: 10.1111/j.1471-6402.1994.tb00451.x {publish Online First 28 July 2006].

71. Quinn-Nilas C, Milhausen RR, Breuer R, Bailey J, Pavlou M, DiClemente RJ, Wingood GM. Validation of the Sexual Communication Self-Efficacy Scale. Health Education & Behavior. 2016 Apr;43(2):165-71.

72. May C. Towards a general theory of implementation. *Implement Sci* 2013;8:18 doi: 10.1186/1748-5908-8-18 [published Online First 13 February 2013].

73. Moore GF, Audrey S, Barker M, et al. Process evaluation of complex interventions: Medical Research Council guidance. *BMJ* 2015;350:h1258 doi: 10.1136/bmj.h1258 [published 19 March 2015].

74. National Institute for Health and Care Excellence. Methods for the development of NICE Public Health Guidance (third edition) https://www.nice.org.uk/process/pmg4/chapter/introduction [published September 2012, accessed September 2017].

75. Edwards RT, Charles JM, Lloyd-Williams H. Public health economics: a systematic review of guidance for the economic evaluation of public health interventions and discussion of key methodological issues. *BMC Public Health* 2013;13:1001 doi: 10.1186/1471-2458-13-1001 [published Online First 24 October 2013].

76. Bailey J, Mann S, Wayal S, et al. Sexual health promotion for young people delivered via digital media: a scoping review. *Public Health Res* 2015;3 doi: 10.3310/phr03130 [published Online First November 2015].

77. Campbell MK, Piaggio G, Elbourne DR, et al. Consort 2010 statement: extension to cluster randomised trials. *BMJ* 2012;345:e5661 doi: 10.1136/bmj.e5661 [published Online First 04 September 2012].

For peer review only - http://bmjopen.bmj.com/site/about/guidelines.xhtml

78. Kahan BC, Jairath V, Doré CJ, et al. The risks and rewards of covariate adjustment in randomized trials: an assessment of 12 outcomes from 8 studies. *Trials* 2014;15:1 doi: 10.1186/1745-6215-15-139 [published Online First 23 April 2014].

79. Kraemer HC, Wilson GT, Fairburn CG, et al. Mediators and moderators of treatment effects in randomized clinical trials. *Arch Gen Psychiatry* 2002;59:877–83 doi: 10.1001/archpsyc.59.10.877

80. Braun V, Clarke V. Using thematic analysis in psychology. *Qual Res Psychol* 2006;3:77–101 doi: 10.1191/1478088706qp063oa [published Online First 21 July 2008].

81. Lincoln YS, Guba EG. Naturalistic Inquiry. Newbury Park CA: Sage; 1985.

82. Hyde A, Howlett E, Brady D, et al. The focus group method: Insights from focus group interviews on sexual health with adolescents. *Soc Sci Med* 2005;61:2588–99 doi: https://doi.org/10.1016/j.socscimed.2005.04.040 [published Online First 13 June 2005].

83. Department of Education NI. Institution search. http://apps.education-ni.gov.uk/appinstitutes/default.aspx [Accessed October 2017].

84. Schulz KF, Altman DG, Moher D, for the CONSORT Group. CONSORT 2010 Statement: updated guidelines for reporting parallel group randomised trials. *BMJ* 2010;340:c332 doi: 10.1136/bmj.c332 [published Online First 24 March 2010].

85. Chan A-W, Tetzlaff JM, Altman, DG, et al. SPIRIT 2013 Statement: Defining standard protocol items for clinical trials. *Ann Intern Med* 2013;158:200-7 doi: 10.7326/0003-4819-158-3-201302050-00583

86. Chan A-W, Tetzlaff JM, Gøtzsche PC, et al. SPIRIT 2013 explanation and elaboration: guidance for protocols of clinical trials. *BMJ* 2013;346:e7568 doi: 10.1136/bmj.e7586 [published Online First 09 January 2013].

For peer review only - http://bmjopen.bmj.com/site/about/guidelines.xhtml

87. Hoffmann TC, Glasziou PP, Boutron I, et al. Better reporting of interventions: template for intervention description (TIDieR) checklist and guide. *BMJ* 2014;348:g1687 doi: 10.1136/bmj.g1687 [published Online First 07 March 2014].

88. WHO Trial Registration Data Set (Version 1.2.1). Available at http://www.who.int/ictrp/network/trds/en/ [Accessed 07.08.17]

89. Vellinga A, Cormican M, Hanahoe B, et al. Opt-out as an acceptable method of obtaining consent in medical research: a short report. *BMC Med Res Meth* 2011;11:40 doi: 10.1186/1471-2288-11-40 [published Online First 6 April 2011].

90. Liu C, Cox RB, Washburn IJ, et al. The effects of requiring parental consent for research on adolescents' risk behaviors: A meta-analysis. *J of Adolesc Health* 2017;61:45-52 doi: 10.1016/j.jadohealth.2017.01.015 [published Online First 28 March 2017].

For peer review only

FIGURE LEGENDS

Figure 1: The JACK Trial Flowchart

Figure 2: School recruitment strategy

Figure 3: *If I Were Jack* Theory of Change Logic Model

Figure 4: Integration of process evaluation with experimental design methodology to achieve research objectives

Figure 5: Current and future impacts of *If I Were Jack* on costs and benefits

For peer review only - http://bmjopen.bmj.com/site/about/guidelines.xhtml

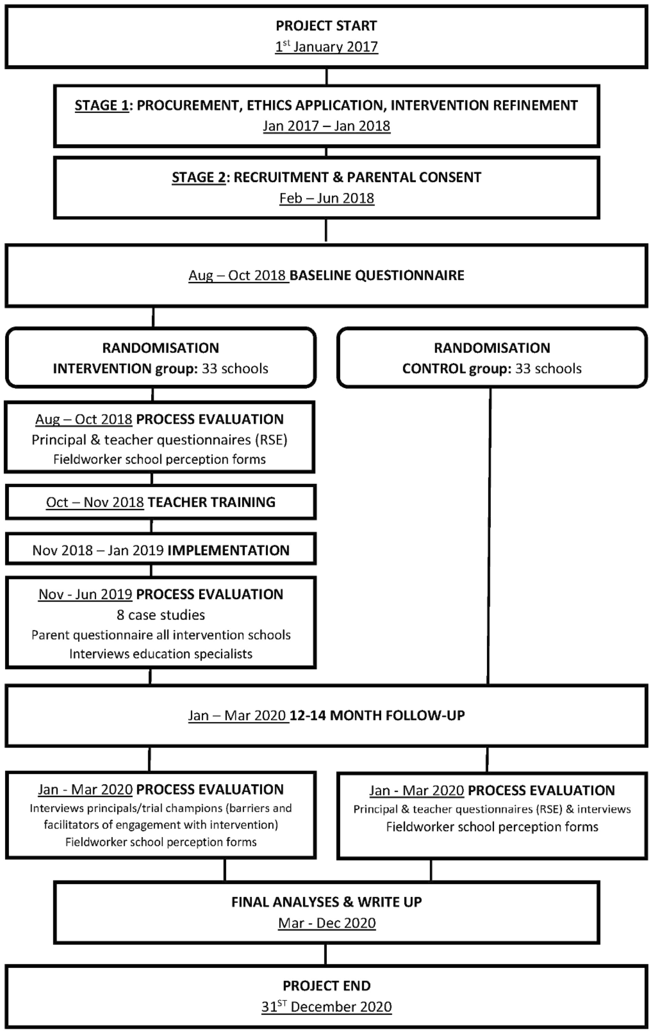

Figure 1: The JACK Trial Flowchart

210x297mm (300 x 300 DPI)

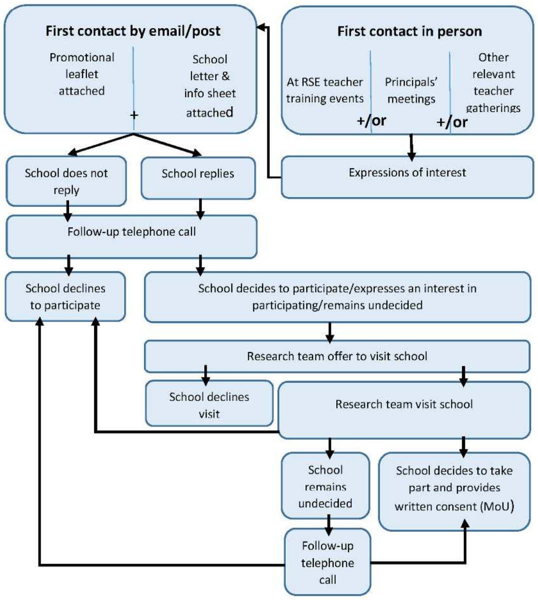

Figure 2: School recruitment strategy

210x297mm (300 x 300 DPI)

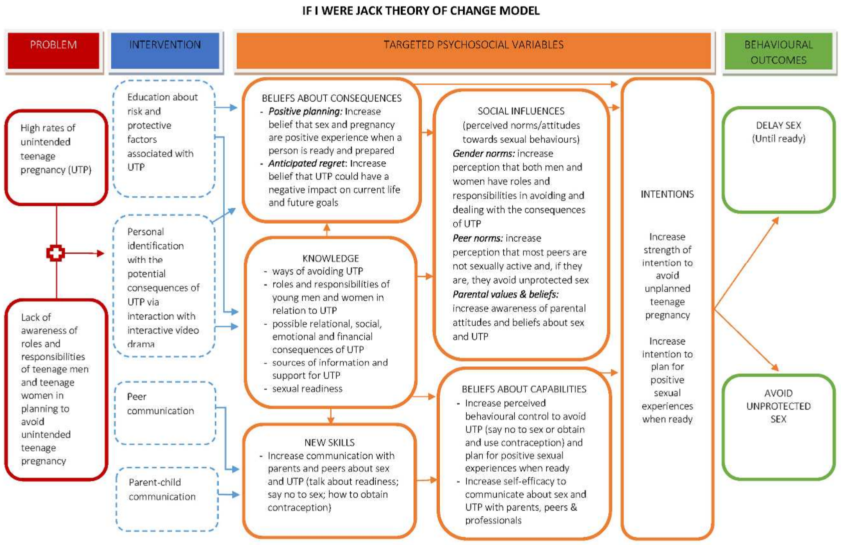

Figure 3: If I Were Jack Theory of Change Logic Model

297x210mm (300 x 300 DPI)

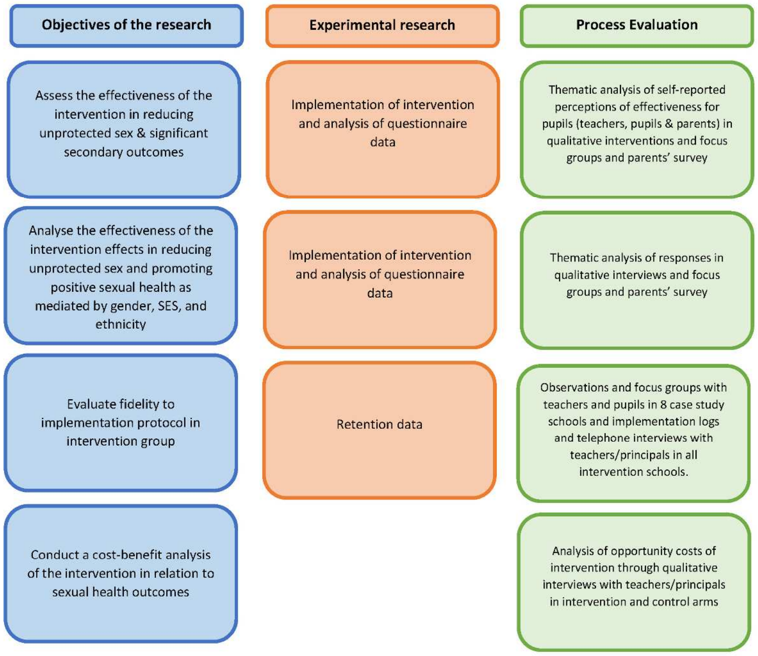

Figure 4: Integration of process evaluation with experimental design methodology to achieve research objectives

210x297mm (300 x 300 DPI)

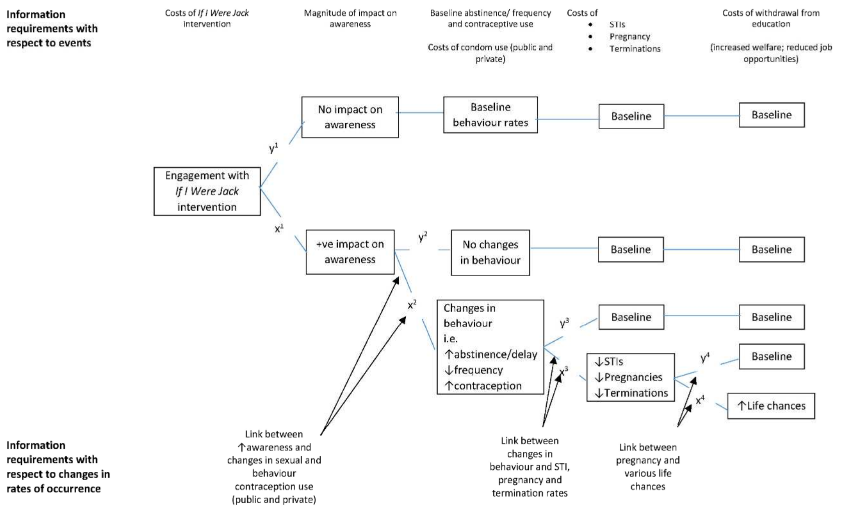

Figure 5: Current and future impacts of If I Were Jack on costs and benefits

297x210mm (300 x 300 DPI)

SPIRIT

STANDARD PROTOCOL ITEMS: RECOMMENDATIONS FOR INTERVENTIONAL TRIALS

SPIRIT 2013 Checklist: Recommended items to address in a clinical trial protocol and related documents*

Section/item	Item No	Description	Addressed on page number
**Administrative information**			
Title	1	Descriptive title identifying the study design, population, interventions, and, if applicable, trial acronym	______1_______
Trial registration	2a	Trial identifier and registry name. If not yet registered, name of intended registry	______2_______
	2b	All items from the World Health Organization Trial Registration Data Set	22 (sup file 3)
Protocol version	3	Date and version identifier	_____________
Funding	4	Sources and types of financial, material, and other support	______24_______
Roles and responsibilities	5a	Names, affiliations, and roles of protocol contributors	______1, 24_____
	5b	Name and contact information for the trial sponsor	_____________
	5c	Role of study sponsor and funders, if any, in study design; collection, management, analysis, and interpretation of data; writing of the report; and the decision to submit the report for publication, including whether they will have ultimate authority over any of these activities	_____24________
	5d	Composition, roles, and responsibilities of the coordinating centre, steering committee, endpoint adjudication committee, data management team, and other individuals or groups overseeing the trial, if applicable (see Item 21a for data monitoring committee)	_____________

For peer review only - http://bmjopen.bmj.com/site/about/guidelines.xhtml

For peer review only

**Introduction**			
Background and rationale	6a	Description of research question and justification for undertaking the trial, including summary of relevant studies (published and unpublished) examining benefits and harms for each intervention	_____4,5______
	6b	Explanation for choice of comparators	_____4,5______
Objectives	7	Specific objectives or hypotheses	_____5,6_____
Trial design	8	Description of trial design including type of trial (eg, parallel group, crossover, factorial, single group), allocation ratio, and framework (eg, superiority, equivalence, noninferiority, exploratory)	_____6_____
**Methods: Participants, interventions, and outcomes**			
Study setting	9	Description of study settings (eg, community clinic, academic hospital) and list of countries where data will be collected. Reference to where list of study sites can be obtained	_____6________
Eligibility criteria	10	Inclusion and exclusion criteria for participants. If applicable, eligibility criteria for study centres and individuals who will perform the interventions (eg, surgeons, psychotherapists)	_____6,7,13_____
Interventions	11a	Interventions for each group with sufficient detail to allow replication, including how and when they will be administered	_____10-13_____
	11b	Criteria for discontinuing or modifying allocated interventions for a given trial participant (eg, drug dose change in response to harms, participant request, or improving/worsening disease)	______________
	11c	Strategies to improve adherence to intervention protocols, and any procedures for monitoring adherence (eg, drug tablet return, laboratory tests)	_____16________
	11d	Relevant concomitant care and interventions that are permitted or prohibited during the trial	______________
Outcomes	12	Primary, secondary, and other outcomes, including the specific measurement variable (eg, systolic blood pressure), analysis metric (eg, change from baseline, final value, time to event), method of aggregation (eg, median, proportion), and time point for each outcome. Explanation of the clinical relevance of chosen efficacy and harm outcomes is strongly recommended	____14,15_____
Participant timeline	13	Time schedule of enrolment, interventions (including any run-ins and washouts), assessments, and visits for participants. A schematic diagram is highly recommended (see Figure)	___Figure 1_____

For peer review only - http://bmjopen.bmj.com/site/about/guidelines.xhtml

Sample size	14	Estimated number of participants needed to achieve study objectives and how it was determined, including clinical and statistical assumptions supporting any sample size calculations	____7,8______
Recruitment	15	Strategies for achieving adequate participant enrolment to reach target sample size	____8-10_______
**Methods: Assignment of interventions (for controlled trials)**			
Allocation:			
Sequence generation	16a	Method of generating the allocation sequence (eg, computer-generated random numbers), and list of any factors for stratification. To reduce predictability of a random sequence, details of any planned restriction (eg, blocking) should be provided in a separate document that is unavailable to those who enrol participants or assign interventions	____10_______
Allocation concealment mechanism	16b	Mechanism of implementing the allocation sequence (eg, central telephone; sequentially numbered, opaque, sealed envelopes), describing any steps to conceal the sequence until interventions are assigned	____10______
Implementation	16c	Who will generate the allocation sequence, who will enrol participants, and who will assign participants to interventions	____10______
Blinding (masking)	17a	Who will be blinded after assignment to interventions (eg, trial participants, care providers, outcome assessors, data analysts), and how	_____6________
	17b	If blinded, circumstances under which unblinding is permissible, and procedure for revealing a participant's allocated intervention during the trial	_____________
**Methods: Data collection, management, and analysis**			
Data collection methods	18a	Plans for assessment and collection of outcome, baseline, and other trial data, including any related processes to promote data quality (eg, duplicate measurements, training of assessors) and a description of study instruments (eg, questionnaires, laboratory tests) along with their reliability and validity, if known. Reference to where data collection forms can be found, if not in the protocol	____15-20_____
	18b	Plans to promote participant retention and complete follow-up, including list of any outcome data to be collected for participants who discontinue or deviate from intervention protocols	_____8-10_____

For peer review only - http://bmjopen.bmj.com/site/about/guidelines.xhtml

Data management	19	Plans for data entry, coding, security, and storage, including any related processes to promote data quality (eg, double data entry; range checks for data values). Reference to where details of data management procedures can be found, if not in the protocol	______________
Statistical methods	20a	Statistical methods for analysing primary and secondary outcomes. Reference to where other details of the statistical analysis plan can be found, if not in the protocol	___20-21____
	20b	Methods for any additional analyses (eg, subgroup and adjusted analyses)	___20-21____
	20c	Definition of analysis population relating to protocol non-adherence (eg, as randomised analysis), and any statistical methods to handle missing data (eg, multiple imputation)	___20-21____
**Methods: Monitoring**			
Data monitoring	21a	Composition of data monitoring committee (DMC); summary of its role and reporting structure; statement of whether it is independent from the sponsor and competing interests; and reference to where further details about its charter can be found, if not in the protocol. Alternatively, an explanation of why a DMC is not needed	____23_________
	21b	Description of any interim analyses and stopping guidelines, including who will have access to these interim results and make the final decision to terminate the trial	____23______
Harms	22	Plans for collecting, assessing, reporting, and managing solicited and spontaneously reported adverse events and other unintended effects of trial interventions or trial conduct	____23_________
Auditing	23	Frequency and procedures for auditing trial conduct, if any, and whether the process will be independent from investigators and the sponsor	______________
**Ethics and dissemination**			
Research ethics approval	24	Plans for seeking research ethics committee/institutional review board (REC/IRB) approval	____23_____
Protocol amendments	25	Plans for communicating important protocol modifications (eg, changes to eligibility criteria, outcomes, analyses) to relevant parties (eg, investigators, REC/IRBs, trial participants, trial registries, journals, regulators)	____22_______

For peer review only - http://bmjopen.bmj.com/site/about/guidelines.xhtml

Consent or assent	26a	Who will obtain informed consent or assent from potential trial participants or authorised surrogates, and how (see Item 32)	____8-10______
	26b	Additional consent provisions for collection and use of participant data and biological specimens in ancillary studies, if applicable	______________
Confidentiality	27	How personal information about potential and enrolled participants will be collected, shared, and maintained in order to protect confidentiality before, during, and after the trial	______________
Declaration of interests	28	Financial and other competing interests for principal investigators for the overall trial and each study site	____24_________
Access to data	29	Statement of who will have access to the final trial dataset, and disclosure of contractual agreements that limit such access for investigators	_____23________
Ancillary and post-trial care	30	Provisions, if any, for ancillary and post-trial care, and for compensation to those who suffer harm from trial participation	______________
Dissemination policy	31a	Plans for investigators and sponsor to communicate trial results to participants, healthcare professionals, the public, and other relevant groups (eg, via publication, reporting in results databases, or other data sharing arrangements), including any publication restrictions	_____23________
	31b	Authorship eligibility guidelines and any intended use of professional writers	_____24,25____
	31c	Plans, if any, for granting public access to the full protocol, participant-level dataset, and statistical code	______________
**Appendices**			
Informed consent materials	32	Model consent form and other related documentation given to participants and authorised surrogates	______________
Biological specimens	33	Plans for collection, laboratory evaluation, and storage of biological specimens for genetic or molecular analysis in the current trial and for future use in ancillary studies, if applicable	______________

*It is strongly recommended that this checklist be read in conjunction with the SPIRIT 2013 Explanation & Elaboration for important clarification on the items. Amendments to the protocol should be tracked and dated. The SPIRIT checklist is copyrighted by the SPIRIT Group under the Creative Commons "Attribution-NonCommercial-NoDerivs 3.0 Unported" license.

For peer review only
For peer review only - http://bmjopen.bmj.com/site/about/guidelines.xhtml

BMJ Open

The JACK Trial Protocol: A Phase III multi-centre cluster randomised controlled trial of a school-based relationship and sexuality education intervention focusing on young male perspectives

Journal:	*BMJ Open*
Manuscript ID	bmjopen-2018-022128.R1
Article Type:	Protocol
Date Submitted by the Author:	04-Jun-2018
Complete List of Authors:	Lohan, Maria ; Queens University of Belfast,<br>Aventin, Áine; Queens University of Belfast<br>Clarke, Mike; Queen's University Belfast, Northern Ireland Methodology Hub<br>Curran, Rhonda; Queens University of Belfast<br>Maguire, Lisa; Queens University of Belfast<br>Hunter, Rachael; University College London, Research Dept of Primary Care and Population Health<br>McDowell, Clíona; Northern Ireland Clinical Trials Unit<br>McDaid, Lisa; University of Glasgow, MRC/CSO Social and Public Health Sciences Unit<br>Young, Honor; Cardiff University, Centre for the Development and Evaluation of Complex Interventions<br>White, James; Cardiff University, South East Wales Trials Unit<br>Fletcher, Adam; Cardiff University, School of Social Sciences<br>French, Rebecca ; London School of Hygiene and Tropical Medicine, Social and Environmental Health Research<br>Bonell, Christopher; London School of Hygiene and Tropical Medicine, Public Health and Policy<br>Bailey, Julia; University College London, Primary Care and Population Health<br>O'Hare, Liam; Queens University of Belfast
**Primary Subject Heading**:	Sexual health
Secondary Subject Heading:	Evidence based practice, Research methods, Sociology, Public health
Keywords:	PUBLIC HEALTH, Community child health < PAEDIATRICS, SEXUAL MEDICINE, PREVENTIVE MEDICINE

SCHOLARONE™
Manuscripts

The JACK Trial Protocol: A Phase III multi-centre cluster randomised controlled trial of a school-based relationship and sexuality education intervention focusing on young male perspectives

Maria Lohan,[1] Áine Aventin,[1] Mike Clarke,[2,3] Rhonda M Curran,[1] Lisa Maguire,[1] Rachael Hunter,[4] Clíona McDowell,[3] Lisa McDaid,[5] Honor Young,[6] James White,[6] Adam Fletcher,[6] Rebecca French,[7] Christopher Bonell,[7] Julia V Bailey,[7] Liam O'Hare[8]

1. School of Nursing and Midwifery, Queen's University Belfast, Belfast, UK

2. Centre for Public Health, Queen's University Belfast, Belfast, UK

3. Northern Ireland Clinical Trials Unit, Belfast, UK

4. Research Department of Primary Care and Population Health, University College London, London, UK

5. MRC/CSO Social & Public Health Sciences Unit, University of Glasgow, Glasgow, UK

6. The Centre for the Development and Evaluation of Complex Interventions for Public Health Improvement (DECIPHer), Cardiff University, Cardiff, UK

7. Department of Social and Environmental Health Research, London School of Hygiene and Tropical Medicine, London, UK

8. Centre for Evidence and Social Innovation, Queen's University Belfast, Belfast, UK

Corresponding Author: Prof Maria Lohan, School of Nursing and Midwifery, Queen's University Belfast, 97 Lisburn Road, Belfast, BT9 7BL, UK Email: m.lohan@qub.ac.uk Telephone: +44(0) 289 90972839

Word count: 6471, including funding statement and acknowledgments & excluding title page, abstract, references, figures and tables

For peer review only - http://bmjopen.bmj.com/site/about/guidelines.xhtml

For peer review only

ABSTRACT

Introduction Teenage pregnancy remains a worldwide health concern which is an outcome of, and contributor to, health inequalities. The need for gender-sensitive interventions with a focus on males in addressing teenage pregnancy has been highlighted as a global health need by the World Health Organisation and identified in systematic reviews of relationship and sexuality education (RSE). This study aims to test the effectiveness of an interactive film-based RSE intervention which draws explicit attention to the role of males in preventing an unintended pregnancy by reducing unprotected heterosexual teenage sex among males and females under age 16 years.

Methods and analysis A Phase III cluster randomised trial with embedded process and economic evaluations. *If I Were Jack* encompasses a culturally sensitive interactive film, classroom materials, a teacher-trainer session and parent animations, and will be delivered to replace some of the usual RSE for the target age group in schools in the intervention group. Schools in the control group will not receive the intervention and will continue with usual RSE. Participants will not be blinded to allocation. Schools are the unit of randomisation stratified per country and socio-economic status. We aim to recruit 66 UK schools (24 in Northern Ireland; 14 in each of England, Scotland and Wales), including approximately 7900 pupils. A questionnaire will be administered at baseline and at 12-14 months post-intervention. The primary outcome is reported unprotected sex, a surrogate measure associated with unintended teenage pregnancy. Secondary outcomes include knowledge, attitudes, skills and intentions relating to avoiding teenage pregnancy in addition to frequency of engagement in sexual intercourse, contraception use, and diagnosis of sexually transmitted infections.

Ethics and dissemination Ethical approval was obtained from Queen's University Belfast. Results will be published in peer-reviewed journals and disseminated to stakeholders. Funding is from the National Institute for Health Research.

Trial registration ISRCTN: 99459996; Pre-results

For peer review only

For peer review only - http://bmjopen.bmj.com/site/about/guidelines.xhtml

STRENGTHS AND LIMITATIONS OF THIS STUDY

- The study is evaluating the first relationship and sexuality education (RSE) intervention to be developed and trialled which explicitly promotes a gender-sensitive approach to addressing teenage pregnancy by focussing on male perspectives.
- The intervention is culturally sensitive to different parts of the UK, non-directive in terms of pregnancy resolution options and sufficiently flexible to allow use within schools which vary in their personal development/RSE policy, including in faith-based schools.
- It is the first RSE Intervention to be developed and trialled across all four nations of the UK, allowing for exploration of what works best where.
- Due to the nature of the intervention and setting – within schools – participating teachers, pupils and parents cannot be blinded to allocation.
- A biological measure of adolescent conception rates was not possible and hence we rely on a surrogate measure of incidence of unprotected sex.

For peer review only

For peer review only - http://bmjopen.bmj.com/site/about/guidelines.xhtml

INTRODUCTION

Teenage pregnancy remains a worldwide health concern and is both an outcome of, and contributor to, inequalities in health.[1] The UK has the highest rate of teenage pregnancy in Western Europe.[2] While conception rates for girls aged under 18 have halved since 1998 in England and Wales, and now stand at 21.0 per 1000 population,[3] it remains that just over 20,000 teenage women under 18 became pregnant in England and Wales in 2015 and approximately half of these ended in legal abortion.[3] The conception rate for Scotland was 32.4 per 1000 in 2015.[4] In Northern Ireland (NI), abortion is illegal and is only considered lawful in exceptional circumstances where the life of the pregnant woman is at immediate risk or if there is a risk of serious injury to her physical or mental health. Reflecting this different legal framework, government targets around reducing teenage pregnancies in NI relate to births and not conceptions. In NI, the birth rate for teenage mothers per 1000 young women aged 13-19 years was 11.3 in 2013.[5] In the same year, the teenage birth rate in the most deprived areas was 23.0 per 1000, nearly six times that of the least deprived areas in NI (3.9 per 1000).[6]

Although the life course for teenage parents is not universally negative,[7] the social disadvantage and exclusion that are linked to teenage pregnancy are considered problematic.[1] Unintended teenage pregnancy can lead to considerable adverse health problems for teenagers and their infants as well as generating emotional, social and economic costs for them, their families and society.[8, 9] While unintended teenage pregnancy is a complex phenomenon that cannot be prevented through Relationship and Sexuality Education (RSE) alone,[10–16] high quality RSE is an essential component in the process of reducing unintended pregnancy rates, as well as being a vital aspect of improving holistic sexual health and wellbeing.[17–21] The UK governments all emphasise the policy importance of decreasing under-18 conception rates and increasing sexual health precaution behaviours in teenagers via the implementation of RSE in schools as a key objective in their current sexual health policies.[22–24]

For peer review only - http://bmjopen.bmj.com/site/about/guidelines.xhtml

Several systematic reviews have identified the characteristics of effective RSE programmes which help increase their impact on sexual risk-taking behaviours.[25-31] These include: the use of theoretically-based interventions targeting sexual and psycho-social mediating variables such as knowledge, attitudes, self-efficacy, intentions, perceptions of risk, and perceptions of peer norms which are linked to sexual behaviour change; the use of culturally-sensitive and gender-specific interventions; the use of interactive modalities which promote personal identification with the educational issues and engagement of young people; the use of skills-building components; the involvement of parents in the RSE process; and facilitating linkages with support services. However, teenage boys have usually been neglected in relation to RSE, particularly with respect to teenage pregnancy.[18, 32–36] The lack of gender-sensitive interventions which acknowledge the potential influence of gender in successfully engaging both males and females in addressing teenage pregnancy has been highlighted as a global health need by the World Health Organisation (WHO)[37-39] and identified in systematic reviews of RSE.[15, 35, 40-41]

The *If I were Jack* teacher-led classroom-based RSE intervention represents an innovative combination of the effective characteristics identified in the above mentioned systematic reviews. It is aimed at both teenage boys and girls but with explicit attention drawn to the role of teenage boys in preventing an unintended teenage pregnancy. A specific aim of *If I Were Jack* is to encourage scrutiny of the gender norms which typically situate the issue of a teenage pregnancy as a woman's problem, by placing emphasis on the teenage male perspective whilst not excluding the teenage female perspective. The *If I Were Jack* intervention is predicted to decrease young people's sexual risk-taking behaviour in relation to avoiding teenage pregnancy and to promote positive sexual health.

We aim to evaluate the effectiveness and cost effectiveness of the *If I Were Jack* RSE intervention in reducing rates of unprotected sex among teenagers under 16 years of age and to better understand the contextual conditions through a process evaluation. The intervention will be delivered to replace

For peer review only - http://bmjopen.bmj.com/site/about/guidelines.xhtml

some of usual RSE in the intervention group. Schools in the control group will not receive the intervention and will continue to deliver RSE according to their current and existing practices, including meeting their statutory curriculum requirements. This is a pragmatic comparator reflecting typical routine practice, which allows for comparison of the intervention with the existing RSE experience.

METHODS AND ANALYSIS

Trial design

The JACK trial is a Phase III multi-centre, parallel-group cluster randomised trial (cRCT) (Figure 1). Schools are the unit of randomisation with a 1:1 allocation. The study design has an embedded process evaluation and economic evaluation. The trial design follows the Medical Research Council's Framework for Developing and Evaluating Complex Interventions.[42]

Study setting

The trial will take place in 66 secondary level schools in the UK (24 in Northern Ireland and 14 each in England, Scotland and Wales). The whole of NI is included but, for reasons of practicality, convenience and cost, representative geographical restrictions will be in place in England (Greater London area), Scotland (mainland Scotland) and Wales (South Wales). The intervention will be delivered by teachers, as part of the Key Stage 4 Personal and Health Education curriculum (NI, England and Wales) and in Scotland as part of the Curriculum for Excellence Relationships, Sexual Health and Parenthood education.

Public Involvement

Public involvement in the design of the If I were Jack intervention has been facilitated by a Young People's Advisory Group (YPAG) composed of members from each of the four nations of the UK. The group of twenty 14-16 year olds and their designated youth workers was brought together for a

For peer review only - http://bmjopen.bmj.com/site/about/guidelines.xhtml

facilitated discussion about the intervention during one weekend in Cardiff in April 2017. The group contributed especially to production decisions for the interactive video drama. This YPAG also read and commented on the questionnaire and pupil information sheet remotely. In the earlier feasibility trial, we had consulted extensively with pupils about the questionnaire.[43] The intervention and trial design has also been informed by a Trial Stakeholders Group. This group is composed of relationship and sexuality education experts and teachers and senior representatives from key statutory organisations and government departments from all four nations of the UK. Finally, the trial design has been informed by a Trial Steering Group, composed of methodological experts, pupils, teachers and school principals/head teachers. All three groups will continue to advise the research team throughout the trial. Dissemination to schools will first involve discussion with Schools and our YPAG involving regular updates and final reports. Our research team across the four nations will disseminate at talks aimed at the public and policy-makers in all four jurisdictions and a lay summary will be made available on our Jack Trial website http://www.qub.ac.uk/sites/if-i-were-jack/

Eligibility

Eligibility criteria for clusters

All state secondary-level schools in the 2018/2019 academic year will be included with the exception of independent private, special and Irish/Welsh-medium and Scottish Gaelic schools (but not excluding schools that have an embedded Irish/Welsh-medium component). Schools with less than 30 pupils in the target year group (Year 11 in NI, S3 in Scotland and Year 10 in England and Wales) will be excluded. Schools that have already participated in the feasibility (n=8 in NI), [43] transferability (England n=3, Scotland n=3 and Wales n=3) and pilot studies (England n=1, Scotland n=1 and Wales n=1) involving the *If I Were Jack* intervention in preparation of this for phase III study will also be excluded.

Eligibility criteria for participants

For peer review only - http://bmjopen.bmj.com/site/about/guidelines.xhtml

Eligible teachers are those who will be responsible for the delivery of RSE to pupils in Year 11 in NI, S3 in Scotland and Year 10 in England and Wales during the 2018/2019 academic year.

Eligible pupils are the 2018/2019 academic year pupil cohort (all classes within) in Year 11 in NI, S3 in Scotland and Year 10 in England and Wales (mean age 14). This year group has been selected for a number of reasons. First, proximal risk factors of teenage pregnancy begin manifesting in this age group,[14, 44] making it an appropriate time for preventative sex education that is considered acceptable in society and education.[14, 27, 45] Second, there is an identified deficit in resources for this age group in relation to teenage pregnancy,[46-47] and third, findings from the JACK feasibility study[43] indicated that there is a greater opportunity for implementation of the intervention during a year where there are no statutory examinations. Finally, this population has been chosen to facilitate a 12-14 month follow-up of pupils (post-intervention) before some pupils exit formal education following their first major statutory exams or reaching the age of 16 years.

Sample size

The sample size calculation is based on UK-wide data[48-49] demonstrating that between 25% and 33% of 15 year olds are having sex and the proportion of 15 year olds reporting unprotected sex is 2.8% (overall in NI, England, Scotland and Wales). The study will be powered to detect a 50% reduction in the incidence of unprotected sex (from expected rate of 2.8% to 1.4%) by 15 years of age. Such a difference of 1.4% in unprotected sex has been shown to have a meaningful impact on pregnancy rates.[14, 50-52] The between-group difference in the incidence of unprotected sex of 1.3% (95% CI 0.5 to 2.2%) by nine months in our feasibility trial[43] demonstrates that such an effect size is plausible and is consistent with effect sizes seen in the literature.[50] The study will take account of clustering. In the feasibility data, the intraclass correlation coefficient (ICC) was 0.01.[43] As pilot studies can provide imprecise estimates of ICCs,[53] we re-estimated using ICCs from three sources, the RIPPLE cRCT,[52] a 2013/2014 WHO Health Behaviour in School-aged Children survey [48] and a 2013 Young Persons' Behaviour and Attitudes Survey in NI conducted by the Northern

For peer review only - http://bmjopen.bmj.com/site/about/guidelines.xhtml

Ireland Statistics & Research Agency (NISRA).[49] The data from the WHO and NISRA studies were combined. The RIPPLE and combined WHO and NISRA studies found an ICC of 0.004. Assuming 120 students per school, an ICC of 0.01 and 7% attrition (plus two additional schools to be conservative), a trial involving 33 schools per group will provide 80% power at a 5% significance level (a pupil participant sample size of n=7904, with n=224 reporting unprotected sex). The power would rise to 93% if the ICC is 0.004.

Recruitment and retention

Recruitment of schools

In each country, eligible schools will be stratified based on the percentage of students eligible for free school meals (%FSM) for the 2018/2019 academic year (schools above and below the median national percentage free school meals) as a proxy for the level of deprivation. In NI, 14 schools will be randomly selected from the above-median stratum and 10 from the below-median stratum (total 24). In England, Scotland and Wales, eight schools will be randomly selected from the above-median stratum and six from the below-median stratum (to give a total of 14). The decision to select slightly more schools from the above-median %FSM reflects research which indicates that teenage pregnancy and unprotected sex is more acute in more deprived areas.[1, 48]

The school recruitment period will run from February to June 2018. The school recruitment strategy is represented in Figure 2. In Scotland, it is required that permission is given from each local authority (typically by approaching the Director of Education) prior to commencing recruitment. Any schools that decline to participate will be replaced by a randomly selected school in the same stratum.

Recruitment of teachers

Based on our recruitment procedures in the feasibility study,[54] once a school has made a decision to participate, a member of the research team will meet with teachers (identified by a school-

For peer review only - http://bmjopen.bmj.com/site/about/guidelines.xhtml

assigned 'Trial Champion') responsible for the delivery of RSE to the target pupil year groups during the 2018/2019 academic year, to deliver an information session and answer any outstanding enquiries. Teachers will be provided with a copy of the school letter, information sheet, memorandum of understanding and consent form.

Recruitment of pupils

When a school and the relevant teachers who will be delivering the intervention to pupils have provided consent, the first step taken towards pupil recruitment is to inform parents/guardians. Schools (with the assistance of the school administrator) will be asked to post a hard copy of the parents'/guardians' information sheet and an opt-out consent form with pre-paid response envelopes. Schools will be responsible for addressing and preparing envelopes for postage. Parents/guardians will be advised within the material provided that they have to return the opt-out consent forms by a date no later than three weeks prior to commencement of baseline data collection within the school. The trial co-ordinator will collate a list of parents/guardians who have opted their child out of participation and return this to participating teachers.

Schools will be provided with printed copies of the pupil information sheets to be distributed to eligible pupils at least one week prior to baseline data collection. Only eligible pupils whose parents/guardians did not opt-out of providing consent for them to participate will be provided with a copy of the pupil information sheet. Immediately prior to administering the baseline questionnaire, eligible pupils will attend a short information session, delivered by a member of the research team, including an information video. Pupils will be given an opportunity to ask questions prior to deciding whether to participate. A repeat information session and baseline data collection session will be facilitated in agreement with the school to accommodate any pupils who are absent from the initial session. In the unlikely event that absenteeism remains in excess of 5-10% in a school, the research team, in agreement with the school, will return a third time to facilitate an

For peer review only - http://bmjopen.bmj.com/site/about/guidelines.xhtml

additional information session and baseline data collection. Pupils with mild learning difficulties or poor English will be supported where possible by fieldworkers to complete the questionnaires.

Retention

To promote school, teacher and pupil retention and complete follow-up, schools will be provided with £1000 upon completion of baseline and follow up measures. Trial co-ordinators will be proactive in resolving any issues that arise with schools. Periodic communications will be provided by trial co-ordinators to inform schools, staff, pupils and parents (depending on preferences of schools) of the current status of the study, and plans for the next phase, as well as to acknowledge their support.

Randomisation

Following baseline data collection, randomisation will be carried out by the NI Clinical Trials Unit (NICTU, a UK Clinical Research Collaboration registered CTU), who will produce eight randomisation schedules (using unique identifiers for schools), one for each %FSM stratum within each country, using random permuted blocks of mixed size, generated using nQuery Advisor 7.0. The NICTU are not involved with recruitment and will only release the randomisation code (in sealed envelopes) when all schools have been recruited and baseline data collection completed, therefore allocation concealment will be ensured.

Intervention

The intervention will be described in accordance with the Template for Intervention Description and Replication (TIDieR) guidelines.[55]

Name and brief description: If I Were Jack is an evidenced-based RSE teacher-delivered intervention designed to prevent unintended pregnancy and promote positive sexual health by increasing teenagers' intentions to avoid teenage pregnancy through delaying sexual intercourse or using contraception consistently. It is especially designed to engage with males but can be delivered to

For peer review only - http://bmjopen.bmj.com/site/about/guidelines.xhtml

both male and female pupils. The underpinning theoretical framework for this intervention combines the well-established Theory of Planned Behaviour [56] and critiques to this theory [57] which focus on the inclusion of an understanding of the broader socio-environmental factors (such as socio-economic status; SES) and underlying values (such as religiosity and gender ideologies) associated with the occurrence of teenage pregnancy.[14, 58-59] The *If I Were Jack* Theory of Change Logic Model is depicted in Figure 3.

Why, rationale of essential elements: If I Were Jack targets six psycho-social mechanisms which research indicates are related to a reduction in risk-taking behaviour: knowledge, communication skills, attitudes, social influences, beliefs about capabilities, and intentions (Table 1).[15, 60, 61]

Table 1: Psychosocial and behavioural components of the *If I Were Jack* intervention

Component	Aim
Knowledge	Increase knowledge of: ways of avoiding unintended pregnancy; roles and responsibilities of young men in relation to unintended pregnancy; possible negative relational, social, emotional and financial consequences of unintended pregnancy; and sources of information and support for unintended pregnancy and sexual health more broadly
Communication skills	Increase skills in communicating with parents, peers and sexual partners about avoiding unintended pregnancy
Attitudes	Increase anticipated regret about the consequences of unintended pregnancy on current and future goals
Social influences	Increase awareness of peer norms, stereotypical gender norms and parental attitudes and beliefs about teenage pregnancy<br>Gender norms: increase perception that both men and women have roles and responsibilities in avoiding and dealing with the consequences of unintended pregnancy<br>Peer norms: increase perception that most peers are not sexually active and use contraception when they are<br>Parental values and beliefs: increase awareness of parental attitudes and beliefs about unintended pregnancy

For peer review only - http://bmjopen.bmj.com/site/about/guidelines.xhtml

Beliefs about capabilities	Increase perceived behavioural control to avoid unintended pregnancy (say no to sex or obtain and use contraception correctly) and increase self-efficacy to communicate about avoiding unintended pregnancy with parents, peers and professionals
Intentions	Increase strength of intention to avoid unplanned teenage pregnancy

The intervention components provide pupils with educational information and opportunities for discussion, skills practice, reflection and anticipatory thinking and are designed to specifically target one or more of the above psycho-social mechanisms.[62] The intervention components also include explicit reference to the impact of SES, religion and gender norms on sexual behaviour, inviting participants to think through how underlying social influences, such as social class and gender norms of sexual behaviour, can be challenged through individual agency. A feasibility trial demonstrated the acceptability to teachers and pupils and feasibility of implementation across a wide range of schools in Northern Ireland.[43]

What, a description of the materials: The *If I Were Jack* intervention consists of the following elements:

- A culturally sensitive interactive video drama (IVD) intended to immerse teenagers in a story of a week in the life of Jack, a teenager who has just been told by his girlfriend that she is pregnant. By asking males and females to imagine they were Jack and how they would think and feel if they were in his situation, it is designed to expose and challenge the gender assumptions around roles and responsibilities for teenage pregnancy by opening them up for reflection and negotiation. Informed by the findings of a prior transferability study that followed the feasibility trial of the intervention,[45] two versions of the IVD have been made available: one for use in England and Wales, using actors with English accents and one for use in NI and Scotland, using actors with NI accents. The IVD is designed to be delivered on individual computers/tablets with the use of headphones.

For peer review only - http://bmjopen.bmj.com/site/about/guidelines.xhtml

- Classroom materials for teachers containing detailed lesson plans with specific classroom-based and homework activities designed to build pupils' skills to a) obtain relevant sexual health information and b) develop communication skills with peers and trusted adults.
- A standardised 60-minute training session for RSE teachers implementing the intervention. The training session will adhere to a pre-defined teacher-trainer protocol and will be delivered in schools by country-specific established statutory and non-statutory RSE co-ordinators who normally provide RSE teacher training in schools.
- Two short animated films to engage parents/guardians and help/encourage them to have a conversation with their teenager about avoiding unintended pregnancy. A link to the web-hosted films will be texted and/or emailed via a school administrator to all parents/guardians of participating pupils in intervention schools (with one additional reminder text/email).
- A dedicated website (www.qub.ac.uk/IfIWereJack) for the intervention, will act as a portal of dissemination, providing password protected access to the intervention materials that teacher-trainers, teachers, parents, and pupils can access.

Who delivers the intervention? It is designed to be delivered by trained RSE teachers.

How, modes of delivery: *If I Were Jack* can be delivered either over four 50-60 minute lessons or over six 35-45 minute lessons and consists of a combination of classroom-based activities (mainly group discussion) having first viewed the IVD, in addition to pupils being asked to engage in two homework activities (one of which involves discussion with parents/guardians). Adherence to the intervention protocol will be determined as part of our process evaluation.

Where, locations where intervention has occurred: The intervention has been delivered in NI[43] and Ireland, using a further locally produced IVD for Ireland. A version of the intervention, the IVD only, has been delivered in schools in South Australia.

Outcomes

For peer review only - http://bmjopen.bmj.com/site/about/guidelines.xhtml

Primary outcome

In this trial, a reduction in unintended teenage pregnancy rates would be the ideal primary outcome measure, but the sample size would need to be very large to detect change in unintended pregnancy rates. We will therefore use a surrogate measure associated with unintended teenage pregnancy: unprotected sex at last sexual encounter, as defined by sexual intercourse without use of contraception (barrier or hormonal). Unprotected sex during teenage years is well established as the main proximate behavioural determinant of teenage pregnancy and is a commonly measured behavioural outcome in studies examining the impact of RSE interventions on teenage pregnancy.[10, 12, 14, 50, 63, 64, 65] Studies indicate that, although other behavioural determinants (such as frequency of sexual intercourse and number of sexual partners) are important, avoidance of unprotected sex via consistent use of contraception is central in explaining variation in levels of teenage pregnancy.[18, 51] The primary outcome will be based on contraception use at last sexual intercourse of unprotected sex at follow-up (i.e. answers to the question "Did you use any form of contraception the last time you had sex?"), consistent with the data on which the sample size calculation was based.[48] An additional item will also be included related to lifetime incidence of unprotected sex in order to account for sporadic use of contraception that may not be reflected in the last sexual encounter. Participants reporting the use of natural family planning or withdrawal methods will be categorised as having engaged in unprotected sex due to the reduced efficacy of these methods in preventing pregnancy and transmission of sexually transmitted infections (STIs). This study will not undertake any data linkage with Health and Social Care or National Health Service records, given that data on conception rates are not available in NI and that data for sexual health related services in England are not readily available as part of routinely collected data given patient privacy requirements.

Secondary outcomes

For peer review only - http://bmjopen.bmj.com/site/about/guidelines.xhtml

Secondary outcomes are 12-month impacts on knowledge, attitudes, skills and intentions to avoiding teenage pregnancy. Secondary outcomes informed by our theory of change (see Figure 3) include knowledge, attitudes, communication skills and intentions relating to avoiding teenage pregnancy at follow-up and are hypothesized to lead to increased intention to avoid unprotected sex. Data will be collected using a number of standardized measures, including comfort communicating about pregnancy and comfort communicating about contraception derived from mathtech behaviour inventory;[65] the male role attitudes scale;[66] sexual socialisation instrument;[67] and sexual self-efficacy scale.[68] We will also collect data using an 'Intentions to avoid a teenage pregnancy scale', developed and psychometrically tested in our feasibility trial.[43] The measures were selected because the constructs they measure map closely to the theoretical framework underpinning the intervention and the reliability and completion rates of the measures were satisfactory in the feasibility trial.[43] In addition, to assist with the economic evaluation, supplementary secondary outcomes include: frequency of engagement in sexual intercourse, contraception use, diagnosis of STIs and incidence of pregnancy and pregnancy outcomes. The collection of this data was also shown to be feasible in the feasibility trial. Finally, we will collect important individual level demographic and socio-economic characteristics of the sample to deepen understandings of how these factors moderate effectiveness.

Data collection

Participating pupils will be in the study for approximately 18 months and asked to complete a paper-based questionnaire during one RSE lesson at baseline and again between 12 and 14 months later. A fieldworker will administer questionnaires to pupils, under exam conditions, two weeks prior to commencement of intervention delivery. Informed by the process evaluation conducted in the feasibility study,[43] teachers will be asked to stay at the front of the classroom to maintain order while alleviating any concerns that teachers may be able to see pupils' answers. Additional

For peer review only - http://bmjopen.bmj.com/site/about/guidelines.xhtml

fieldworkers will be available to provide support to pupils who require extra help and to ensure questionnaires are completed confidentially.

Primary outcome

Process evaluation

Informed by realist approaches to the evaluation of interventions,[42, 69, 70] the process evaluation has four aims. First, we will examine reasons for school participation and non-participation to inform risk of bias in the trial as well as longer term sustainability of implementation of the intervention. Second, we will examine intervention delivery and fidelity in the context of overall RSE provision in intervention schools. Third, we will assess provision in control schools and potential contamination caused by any changes to provision that could be due to participation in the trial. Fourth, we will explore self-reported perceptions of effectiveness and moderating influences in intervention schools among a sample of pupils, teachers and school principals and parents. Triangulated data collection methods will include semi-structured interviews with teachers, focus group discussions with pupils, observations of a sample of lessons and a survey of parents/guardians with follow-up focus groups. For detail on our approach to integration of the process evaluation with the experimental design methodology to achieve research objectives, see Figure 4.

All schools

The school-assigned trial champion or a suitable person identified by the trial champion will complete a school background questionnaire, designed to detail more general information pertinent to trial implementation i.e. school experience of teenage pregnancy, school holidays/closures, school experience of pupils/parents or guardians who do not speak English as a first language or who do not understand English at all, and school involvement in other research. An appropriate member of staff identified by the school-assigned trial champion will also complete a questionnaire about current RSE provision in the school to gain a better understanding of the nature, quantity and quality

For peer review only - http://bmjopen.bmj.com/site/about/guidelines.xhtml

of RSE currently taught within the school as well as the facilitators and barriers to current RSE provision within the school. A school administrator will be asked to fill out a school administrator resource use record detailing associated costs (i.e. postage of parent/guardian information) and time spent. Schools will be reimbursed up to the value of £100 for these costs.

Intervention schools

Parent/guardian online survey: Parents/guardians will have been made aware when they were in receipt of the parents'/guardians' information sheet that there would be an opportunity for them to respond to a short online parent/guardian survey. Eligible parents/guardians (who have not opted out of providing consent for their child to participate) will receive the link to this survey (hosted using the SurveyMonkey® UK platform) in an email and/or text message issued by the school administrator inviting them to participate (post intervention delivery). The survey will ask parents/guardians about their engagement with and opinion of the parent/guardian animations and homework session and whether their child has discussed with them their experience of engaging with *If I Were Jack*. The questionnaire will be translated where possible and where necessary.

Teacher implementation log: Teachers responsible for delivering the intervention will be asked to complete an implementation log to detail what activities were completed or not completed during each lesson, and perception of pupil engagement with each activity.

Telephone interviews: 15-30 minute telephone interviews will be conducted by trial co-ordinators with school principals or trial champions in intervention schools that are not 'case study schools' (see below) to determine any barriers or facilitators of engagement with the intervention.

Case study schools

Participating intervention schools will be randomly rank ordered in each country and two case study schools from each country will be randomly selected by NICTU to participate in the process evaluation. Should a school refuse participation, a further random selection will be made.

For peer review only - http://bmjopen.bmj.com/site/about/guidelines.xhtml

Observations: Country-specific trial co-ordinators will conduct structured observations of one randomly selected lesson in every class group in receipt of the intervention in the eight case study schools. Observations will be focused primarily on measuring teacher fidelity to implementation protocol and pupil engagement.

Focus groups: Trial co-ordinators will conduct three 60-minute focus group discussions in each of the eight case study schools. One group will be composed of all teachers who delivered the intervention. The second group will include a maximum of six English-speaking pupils who received the intervention. Teachers who delivered the intervention will ask for a mixture of male and female pupil volunteers and pass details of those pupils to the trial co-ordinator. In the event that more pupils volunteer than are needed (per school), a random selection will be made. The third group will be a maximum of six English-speaking parents/guardians (of children who received the intervention). Discussions will focus on perceived barriers and facilitators of successful implementation and engagement with different components of the intervention.

Fieldworkers

Fieldworkers will complete a fieldworker perception form after each visit to a school, asking them to detail what worked well and what did not in relation to data collection and any other relevant observations they may have.

Education/policy specialists

Trial co-ordinators will conduct telephone or face-to-face interviews with one or two education/policy specialists in each country. Interviews will focus on the current context of RSE policy and perceptions of how this might influence the uptake of the *If I Were Jack* intervention.

Economic evaluation

The economic evaluation will aim to describe the costs and consequences of implementing *If I Were Jack* in UK schools so as to provide information to decision makers on the implications of potential

For peer review only - http://bmjopen.bmj.com/site/about/guidelines.xhtml

further roll-out of the intervention. This will include the duration of time taken up by *If I Were Jack* in school from the perspective of the teacher and impact on time spent on other important curricula activities compared to time spent on standard RSE. The aim of this will be to provide a measure of the opportunity cost to schools of implementing *If I Were Jack* compared to current RSE. The structure of the evaluation will follow NICE guidance for evaluating public health interventions [71] and recent guidance published by Edwards et al [72] on economic evaluations in public health. Costs will include the cost of implementing the intervention in schools including any training involved and the cost of current RSE in the control schools. We will also collect information on healthcare cost information in the intervention and control arms including the costs of sexual health related primary care attendances, costs associated with STIs and unintended pregnancies (although numbers of these are likely to be small). The cost of adapting *If I Were Jack* to different groups will also be reported given that others may want to adapt the intervention before rolling it out. Mean cost per pupil will be reported alongside consequences including use of contraception, STIs and unintended pregnancies collected using questionnaires administered to pupils at baseline and follow-up. Although 12-14 month recall time is a relatively long time-period, pupils are likely to be able to recall high impact events that occurred during this period. The follow-up time is also important to fit within the school year timetable. Costs will also be reported by country, given the different sexual health services provided and hence differential implications for health service costs by country.

Given that STIs and unintended pregnancies are likely to be rare but potentially high impact events in this population group, the long-term costs and consequences will be modelled as part of the cost-effectiveness decision model (Figure 5), incorporating theories of behaviour change and identified as applicable for use in this trial during the feasibility trial.[45] In addition to collecting information as part of the trial, we will look to systematic reviews of evidence of the impact of digital interventions on sexual health behaviour in this population group, for example the review recently undertaken and published by Bailey et al.[73] We will undertake one way, two way and probabilistic sensitivity analyses of the results. Cost effectiveness acceptability curves and cost-effectiveness planes will be

For peer review only - http://bmjopen.bmj.com/site/about/guidelines.xhtml

reported. The model will have a 20-year time horizon and discounting of future costs and benefits will comply with NICE guidance for evaluating public health interventions.[71]

Statistical Methods

The reporting and presentation of findings will be in accordance with the CONSORT guidelines for cRCTs.[74] All analyses will take account of clustering by school using robust standard errors, and intervention and control groups will be compared at baseline via frequencies/descriptive statistics (percentage, mean or median as appropriate) in relation to sex, ethnicity, SES at school level (using percentage free school meals and post-code data) and at individual level (using highest education qualifications of parents), primary and secondary outcomes.

Primary analysis (12-14 month follow-up): The primary effectiveness analysis will be on an intention to treat basis, using a multi-level logistic regression model (two levels: pupils nested within schools) adjusting for the baseline outcome and stratification variables.[75] Sensitivity analyses, making different assumptions on the best and worst case scenarios, as well as imputation models of missingness will be conducted to investigate the potential impact of missing data.

Secondary analysis (12-14 month follow-up): Although the trial is not powered to detect the influence of mediating and moderating variables, we will examine the following outcomes informed by our theory of change model (see Figure 3): i) interaction terms will be used to investigate possible differences in the effect of the intervention on the primary outcome by whether pupils at baseline reported having had unprotected sex or not, country (Wales, England, Scotland, NI), sex, socio-economic group (see earlier section 5.5) and ethnicity); ii) a mediational analysis, using an analytic framework recommended for RCTs,[76] will be used to explore whether the effect of the intervention on the primary outcome is mediated by individual-level sexual health knowledge and sexual competence, perceived behavioural control, intentions to avoid an unintended pregnancy, communication with parents, and gender ideologies. In these secondary analyses, p-values will be

For peer review only - http://bmjopen.bmj.com/site/about/guidelines.xhtml

interpreted with caution due to the low power and number of interactions being tested (e.g. use of Bonferroni corrected p-values).

Process evaluation

All data will be transcribed verbatim (in the case of interviews) or written up in detail (in the case of observational field notes and other secondary source data). These data will be organised using 'NVivo' software and analysed systematically and thematically based on the six steps proposed by Braun and Clarke[77] to enable identification and analysis of patterns (or 'themes') within the data by moving iteratively between theoretical understandings and the new data. These inductively and deductively derived codes will be analysed to form overarching themes emerging from each of the participant groups outlined above. We will use qualitative software 'NVivo 10' to organise the data, and we will ensure methodological rigour by establishing credibility, transferability, dependability and confirmability using techniques suggested by Lincoln and Guba.[78] In addition, following Hyde et al,[79] specific attention will be given to analysing the group dynamics of the focus groups as part of the overall interpretive process.

DISCUSSION

The strengths of this study include that this is the first RSE intervention to be developed and trialled which explicitly promotes a gender-sensitive approach to addressing teenage pregnancy by focussing on male perspectives. The intervention is culturally sensitive to different parts of the UK, is non-directive in terms of pregnancy resolution options and is sufficiently flexible to be taught within the framework of a school's ethos and personal development/RSE policy, including in faith-based schools. This has particular significance in NI, with almost half (46%) of all NI grammar and secondary schools identifying as a Roman-Catholic school.[80] This is also the first RSE intervention to be developed and trialled across all four nations of the UK, allowing for exploration of what works best where. An additional strength of the study is that the embedded process evaluation involves triangulation of sources including school management, teachers, pupils, parents and RSE statutory

For peer review only - http://bmjopen.bmj.com/site/about/guidelines.xhtml

and voluntary stakeholders. Study limitations include that the pragmatic setting – within schools – means that schools and participating teachers, pupils and parents will remain unblinded to the allocation. Finally, the use of the surrogate measure of unprotected sex rather than a biological measure (such as conception rates) introduces the possibility that the findings of the trial will be influenced by self-report bias, but the veracity of this measure is enhanced by privacy, confidentiality and a control group.

ETHICS AND DISSEMINATION

In writing this protocol, we have endeavoured to adhere to the recommendations and guidance provided in the Consolidated Standards of Reporting Trials (CONSORT) 2010 statement and the extension for cRCTs,[74, 81] the Standard Protocol Items: Recommendations for Intervention Trials (SPIRIT) Statement 2013[82, 83] and the Template for Intervention Description and Replication (TIDieR).[55] When registering the trial, we have provided structured summary information in accordance with the requirements of the WHO Trial Registration Data Set (Supplementary File 3).[84]

Any modifications to the protocol which may impact on the conduct of the study, potential effectiveness, or impact study participants, including changes of study objectives, study design, sample size, study procedures or significant administrative aspects will require a formal amendment to the protocol. Such amendment will be agreed upon by the JACK Trial Steering Committee, reported to the funder (National Institute of Health Research, NIHR) and approved by the ethics committee prior to implementation. Minor administrative changes, corrections or clarifications that have no effect on the way the study is to be conducted will be agreed upon by the JACK Trial Steering Committee, documented and reported to the NIHR. The ethics committee may be notified of such changes at the discretion of the JACK Trial Steering Committee.

A full study report will be submitted to the NIHR by the end of December 2020 and made publically available thereafter in the Public Health Research journal on the NIHR Journals Library. We shall

For peer review only - http://bmjopen.bmj.com/site/about/guidelines.xhtml

make data available to the scientific community with as few restrictions as feasible, following receipt of a request to the corresponding author, while retaining exclusive use until the publication of major outputs via academic conference presentations and journal articles in addition to material created for relevant stakeholders. Pupil friendly brief reports will be provided to all participating schools.

Ethical approval for the JACK Trial was granted by the Research Ethics Committee of the School of Nursing and Midwifery, Queen's University Belfast in July 2017 (Ref: 11.MLohan.05.17.M6.V1).

Safety and data monitoring

This is a low-risk study, therefore, a Data Monitoring Committee is not required and no interim analysis is planned.

ACKNOWLEDGEMENTS

In addition to the authors listed, the JACK Trial study thus far has been dependent on the commitment provided by extensive public involvement. The intervention has been designed, developed and piloted in Ireland, NI and South Australia involving over ten years of research with pupils, teachers, sex education specialists, and education and health promotion departments. These collaborations have influenced the study by allowing different perspectives to inform the design and optimal conditions of implementation. We specifically acknowledge the role of the Trial Steering Committee, comprised of trials experts, a school principal, a school teacher, parent and pupils, who have provided independent expert advice on the intervention itself as well as refining research methods for delivery in schools. We also wish to acknowledge our Stakeholders Group, composed of a UK-wide group of RSE specialists and senior representatives from key statutory organisations and government departments who have helped to refine the intervention and the study design by ensuring the research outcomes are important to public concerns and the methods proposed are acceptable and sensitive to all study participants. We would also like to acknowledge the invaluable involvement of our Young People's Advisory Groups (one in each country in the UK) who have

For peer review only - http://bmjopen.bmj.com/site/about/guidelines.xhtml

enabled to us to further refine the intervention in a culturally sensitive way for effectiveness testing in the whole of the UK. Finally, we thank all schools, teachers, parents and pupils who have participated in the feasibility, transferability and pilot studies that have preceded this trial.

FUNDING STATEMENT

This research is funded by the National Institute for Health Research (NIHR) Public Health Research programme, grant number NIHR PHR 15/181/01. LMc is funded by the UK Medical Research Council (MRC) and Scottish Government Chief Scientist Office (CSO) at the MRC/CSO Social & Public Health Sciences Unit, University of Glasgow (MC_UU_12017/11, SPHSU11). The views expressed are those of the authors and not necessarily those of the NHS, the NIHR or the Department of Health. This funding source had no role in the design of this study and will not have any role during its execution, analyses, interpretation of the data, or decision to submit results.

COMPETING INTERESTS STATEMENT

QUB holds copyright and the researchers do not benefit financially from its evaluation or use. We report no competing interests or conflicts of interest.

TRIAL STATUS

At the time of submission of this protocol (January 2018) optimisation of the intervention is underway, but no aspect of the trial has begun yet.

AUTHORS' CONTRIBUTIONS

ML, AA, MC and LM conceived of the study and led study design. RC and ML drafted this manuscript. All authors made substantive contributions to the development of the protocol, critically reviewed and gave final approval to the manuscript. AA is responsible for management of the trial and process evaluation supported by ML. HY, AF, LMc, JW, CB, RF, LO'H and JB contributed to the design of study and development of trial recruitment areas and PPI involvement. HY and AF led on young people's advisory group. CM and JW contributed to sample size calculation and statistical analysis plan. RH

For peer review only - http://bmjopen.bmj.com/site/about/guidelines.xhtml

For peer review only

developed the economic evaluation. LO'H contributed to knowledge translation and dissemination plan.

REFERENCES

1. Harden A, Brunton G, Fletcher A, et al. Teenage pregnancy and social disadvantage: systematic review integrating controlled trials and qualitative studies. *BMJ* 2009;339:b4254 doi: 10.1136/bmj.b4254 [published Online First: 13 November 2009].

2. Office of National Statistics (2014) International comparisons of teenage births 2014. http://web.ons.gov.uk/ons/rel/vsob1/births-by-area-of-usual-residence-of-mother--england-and-wales/2012/sty-international-comparisons-of-teenage-pregnancy.html (Accessed May 20th 2016)

3. Office of National Statistics. Dataset: 2015 Conception Statistics, England and Wales https://www.ons.gov.uk/peoplepopulationandcommunity/birthsdeathsandmarriages/conceptionandfertilityrates/datasets/conceptionstatisticsenglandandwalesreferencetables [Accessed October 2017].

4. NHS Scotland Information Services Division. Teenage Pregnancy Year of conception ending 31 December 2015. https://www.isdscotland.org/Health-Topics/Sexual-Health/Publications/2017-07-04/2017-07-04-TeenPreg-Summary.pdf [published 04 July 2017, Accessed October 2017].

5. NISRA. Births (administrative geographies) 1999-2014. Northern Ireland Statistics & Research Agency (NISRA); 2015.

6. Department of Health Social Services and Public Safety. NI health and social care inequalities monitoring system (HSCIMS) - Health inequalities in Northern Ireland: key facts 2015; https://www.health-ni.gov.uk/sites/default/files/publications/dhssps/hscims-2015-key-facts.pdf [published 2015, Accessed September 2017].

7. Bonell C. Why is teenage pregnancy conceptualized as a social problem? A review of quantitative research from the USA and UK. *Cult Health Sex* 2004;6:255–72 doi:

For peer review only - http://bmjopen.bmj.com/site/about/guidelines.xhtml

10.1080/13691050310001643025 [published Online First: 6 October 2011].

8. Beers LAS, Hollo RE. Approaching the adolescent-headed family: a review of teen parenting. *Curr Probl Pediatr Adolesc Health Care* 2009;39:216–33 doi: 10.1016/j.cppeds.2009.09.001 [published Online First 24 October 2009].

9. Francesconi M. Adult outcomes for children of teenage mothers. *Scand J Econ* 2008;110:93–117 doi: 10.1111/j.1467-9442.2008.00526.x [published Online First March 2008].

10. Wight D, Raab GM, Henderson M, et al. Limits of teacher delivered sex education: interim behavioural outcomes from randomised trial. *BMJ* 2002;324:1430 doi: 10.1136/bmj.324.7351.1430 [published Online First 15 June 2002].

11. Silva M. The effectiveness of school-based sex education programs in the promotion of abstinent behavior: a meta-analysis. *Health Educ Res* 2002;17:471–81 doi: 10.1093/her/17.4.471 [published Online First 01 August 2002].

12. Stephenson JM, Oakley A, Johnson AM, et al. A school-based randomized controlled trial of peer-led sex education in England. *Control Clin Trials* 2003;24:643–57 doi: 10.1016/S0197-2456(03)00070-9 [published Online First 13 September 2003].

13. Fullerton D. Promoting Positive Adolescent Sexual Health & Preventing Teenage Pregnancy-of Recent Effectiveness Research. Dublin: Crisis Pregnancy Programme http://crisispregnancy.ie/wp-content/uploads/2012/05/Rep22.-promoting-positive-adolescent-sexual-health-and-preventing-teenage-pregnancy-a-review-of-recent-effectiveness-research.pdf [published 2004, Accessed September 2017].

14. Henderson M, Wight D, Raab GM, et al. Impact of a theoretically based sex education programme (SHARE) delivered by teachers on NHS registered conceptions and terminations: final results of cluster randomised trial. *BMJ* 2007;334:133 doi: 10.1136/bmj.39014.503692.55 [published Online First 18 January 2007].

For peer review only - http://bmjopen.bmj.com/site/about/guidelines.xhtml

15. Shepherd J, Kavanagh J, Picot J, et al. The effectiveness and cost-effectiveness of behavioural interventions for the prevention of sexually transmitted infections in young people aged 13-19: a systematic review and economic evaluation. *Health Technol Assess* 2010;14:1–206 doi: 10.3310/hta14070 [published Online First February 2010].

16. Elliott L, Henderson M, Nixon C, et al. Has untargeted sexual health promotion for young people reached its limit? A quasi-experimental study. *J Epidemiol Community Health* 2013;67:398–404 doi: 10.1136/jech-2012-201034 [published Online First 20 February 2013].

17. Downing J, Jones L, Cook P, et al. Prevention of sexually transmitted infections (STIs): a review of reviews into the effectiveness of non-clinical interventions: Evidence Briefing Update https://www.nice.org.uk/guidance/ph3/evidence/evidence-briefing-update-prevention-of-sexually-transmitted-infections-stis-2006-65843250 [published 2006, Accessed September 2017].

18. Swann C, Bowe K, McCormick G, et al. Teenage pregnancy and parenthood: a review of reviews. Evidence briefing for Health Development Agency. London: Health Development Agency; 2003.

19. Ellis S, Grey A. Prevention of Sexually Transmitted Infections (STIs): a review of reviews into the effectiveness of non-clinical interventions to reduce the risk of sexual transmission. Evidence Briefing. London: Health Development Agency; 2004.

20. Ingham R, Hirst J. Promoting sexual health. In: Dennison C, Warwick I, editors. Promoting Health and Wellbeing in Schools. London: Routledge; 2010.

21. Oringanje C, Meremikwu MM, Eko H, et al. Interventions for preventing unintended pregnancies among adolescents. *Cochrane Database Syst Rev* 2009;3;2:CD005215 doi: 10.1002/14651858.CD005215.pub3 [published Online First 3 February 2016].

22. National Institute for Health and Care Excellence. Sexually transmitted infections and under-18 conceptions: prevention; 2007.

For peer review only - http://bmjopen.bmj.com/site/about/guidelines.xhtml

23. Committee for Health, Social Services and Public Safety, Northern Ireland Assembly. Official Report (Hansard) Sexual Health Promotion Strategy and Action Plan 2008-2013 http://www.niassembly.gov.uk/globalassets/documents/official-reports/health/2011-2012/sexual-health-promotion-strategy-and-action-plan-2008-2013.pdf [published 18 April 2012, Accessed September 2017].

24. The Scottish Government. Pregnancy and Parenthood in Young People Strategy 2016-2026 https://www.scottishwomensconvention.org/content/outcomes-scotland-2016/Pregnancy-and-Parenthood.pdf [published March 2016, Accessed September 2017].

25. Kirby D, Laris B, Rolleri L. Sex and HIV education programs for youth: Their impact and important characteristics http://recapp.etr.org/recapp/documents/programs/SexHIVedProgs.pdf [published 13 May 2006, Accessed September 2017].

26. Kirby D. Effective approaches to reducing adolescent unprotected sex, pregnancy, and childbearing. *J Sex Res* 2002;39:51–7 doi: 10.1080/00224490209552120 [published Online First 11 January 2010].

27. Kirby D. Emerging Answers 2007: Research findings on programs to reduce teen pregnancy and sexually transmitted diseases. Washington DC: National Campaign to Prevent Teen and Unplanned Pregnancy http://www.siecus.org/index.cfm?fuseaction=Feature.showFeature&featureID=1151 [published November 2007, Accessed September 2017].

28. Robin L, Dittus P, Whitaker D, et al. Behavioral interventions to reduce incidence of HIV, STD, and pregnancy among adolescents: a decade in review. *J Adolesc Health* 2004;34:3–26 doi: 10.1016/S1054-139X(03)00244-1 [published Online First 29 December 2003].

29. Bailey JV, Murray E, Rait G, et al. Interactive computer-based interventions for sexual health promotion. *Cochrane Database Syst Rev* 2010;9:CD006483 doi: 10.1002/14651858.CD006483.pub2 [published Online First 8 September 2010].

For peer review only - http://bmjopen.bmj.com/site/about/guidelines.xhtml

30. Guse K, Levine D, Martins S, et al. Interventions using new digital media to improve adolescent sexual health: A systematic review. *J Adolesc Health* 2012;51:535–43 doi: 10.1016/j.jadohealth.2012.03.014 [published First Online 4 May 2012].

31. Noar SM, Pierce LB, Black HG. Can Computer-Mediated Interventions Change Theoretical Mediators of Safer Sex? A Meta-Analysis. *Hum Commun Res* 2010;36:261–97 doi: 10.1111/j.1468-2958.2010.01376.x [published Online First 7 June 2010].

32. Lohan M, Cruise S, O'Halloran P, et al. Adolescent men's attitudes and decision-making in relation to an unplanned pregnancy. Responses to an interactive video drama. *Soc Sci Med* 2011;72:1507–14 doi: 10.1016/j.socscimed.2011.02.044 [published Online First 24 March 2011].

33. Smith LH, Guthrie BJ, Oakley DJ. Studying adolescent male sexuality: Where are we? *J Youth Adolesc* 2005;34:361–77 doi: 10.1007/s10964-005-5762-5

34. Guttmacher Institute. In their own right: Addressing the sexual and reproductive health needs of American men https://www.guttmacher.org/report/their-own-right-addressing-sexual-and-reproductive-health-needs-american-men [published October 2003, Accessed September 2017].

35. Marsiglio W, Ries A, Sonenstein F, et al. It's a Guy Thing: Boys, Young Men, and Teen Pregnancy Prevention. The National Campaign to Prevent Teen Pregnancy https://thenationalcampaign.org/resource/its-guy-thing [published February 2006, Accessed September 2017].

36. Lindberg LD, Sonfield A, Gemmill A. Reassessing adolescent male sexual and reproductive health in the United States: research and recommendations. *Am J Mens Health* 2008;2:40–56 doi: 10.1177/1557988307309460 [published Online First 5 December 2007].

37. WHO. Evidence for gender responsive actions to prevent and manage adolescent pregnancy http://www.euro.who.int/__data/assets/pdf_file/0008/158093/316637_WHO_brochure_226x226_5-AdolecentPregnancy.pdf [published 2011, Accessed September 2017].

For peer review only - http://bmjopen.bmj.com/site/about/guidelines.xhtml

38. WHO. Health 2020: a European policy framework supporting action across government and society for health and well-being http://www.euro.who.int/__data/assets/pdf_file/0006/199536/Health2020-Short.pdf?ua=1 [published 2013, Accessed Setember 2017].

39. WHO. Health policy framework supporting action across government and society for health and well-being. 2012.

40. Ries A, Sonenstein F. What works for boys: A re-examination of coeducational school curricula. In: Troccoli K, editor. It's a guy thing boys young men and teen pregnancy prevention. Washington DC: National Campaign to Prevent Teen Pregnancy 2006:101–38.

41. Bennett SE, Assefi NP. School-based teenage pregnancy prevention programs: a systematic review of randomized controlled trials. *J Adolesc Health* 2005;36:72–81 doi: https://doi.org/10.1016/j.jadohealth.2003.11.097 [published Online First 18 January 2005].

42. Craig P, Dieppe P, Macintyre S, et al. Developing and evaluating complex interventions: the new Medical Research Council guidance. *BMJ* 2008;337:a1655 doi: 10.1136/bmj.a1655 [published Online First 29 September 2008].

43. Lohan M, Aventin Á, Maguire L, et al. Increasing boys' and girls' intention to avoid teenage pregnancy: a cluster randomised control feasibility trial of an interactive video drama based intervention in post-primary schools in Northern Ireland. *Public Health Res* 2017;5 doi: 10.3310/phr05010 [published Online First March 2017].

44. Crawford C, Cribb J, Kelly E. Teenage Pregnancy in England. Centre for the Analysis of Youth Transitions https://www.ifs.org.uk/publications/6702 [published 21 May 2013, Accessed September 2017].

45. Jones L, Bates G, Downing J, et al. A review of the effectiveness and cost-effectiveness of personal social and health education in secondary schools focussing on sex and relationships and

For peer review only - http://bmjopen.bmj.com/site/about/guidelines.xhtml

alcohol education for young people aged 11-19 years. Final Report https://www.nice.org.uk/guidance/gid-phg0/documents/pshe-evidence-review-secondary-education-and-fe2 [published October 2009, Accessed September 2017].

46. Education and Training Inspectorate NI. Report of an evaluation of relationships and sexuality education in post-primary schools https://www.etini.gov.uk/publications/report-evaluation-relationships-and-sexuality-education-post-primary-schools [published 11 January 2011, Accessed September 2017].

47. Ofsted. Not yet good enough: personal, social, health and economic education in schools http://webarchive.nationalarchives.gov.uk/20141107025925/http://www.ofsted.gov.uk/resources/not-yet-good-enough-personal-social-health-and-economic-education-schools [published 01 May 2013, Accessed September 2017].

48. WHO, 2016. Growing up unequal: gender and socioeconomic differences in young people's health and well-being. Health Behaviour in School-aged Children (HBSC) study: international report from the 2013/2014 survey. Health Policy for Children and Adolescents, No. 7; 2016 http://www.euro.who.int/__data/assets/pdf_file/0003/303438/HSBC-No.7-Growing-up-unequal-Full-Report.pdf?ua=1 [Accessed September 2017].

49. Northern Ireland Statistics and Research Agency. Central Survey Unit. (2015). Young Persons' Behaviour and Attitudes Survey, 2013. [data collection]. UK Data Service. SN: 7624, http://doi.org/10.5255/UKDA-SN-7624-1

50. Chin HB, Sipe TA, Elder R, et al. The effectiveness of group-based comprehensive risk-reduction and abstinence education interventions to prevent or reduce the risk of adolescent pregnancy, human immunodeficiency virus, and sexually transmitted infections: two systematic reviews for the Guide to Community Preventive Services. *Am J Prev Med* 2012;42:272–94 doi: 10.1016/j.amepre.2011.11.006 [published Online First 14 February 2012].

For peer review only - http://bmjopen.bmj.com/site/about/guidelines.xhtml

For peer review only

51. Santelli JS, Lindberg LD, Finer LB, et al. Explaining recent declines in adolescent pregnancy in the United States: the contribution of abstinence and improved contraceptive use. *Am J Public Health* 2007;97:150–6 doi: 10.2105/AJPH.2006.089169 [published Online First 10 October 2011].

52. Parkes A, Wight D, Henderson M, et al. Contraceptive method at first sexual intercourse and subsequent pregnancy risk: findings from a secondary analysis of 16-year-old girls from the RIPPLE and SHARE studies. *J Adolesc Health* 2009;44:55–63 doi: 10.1016/j.jadohealth.2008.06.006 [published Online First 18 October 2008].

53. Eldridge SM, Costelloe CE, Kahan BC, et al. How big should the pilot study for my cluster randomised trial be? *Stat Methods Med Res* 2016;25:1039-56 doi: 10.1177/0962280215588242 [published First Online 12June 2015].

54. Aventin A, Lohan M, Maguire L, et al. Recruiting faith- and non-faith-based schools, adolescents and parents to a cluster randomised sexual-health trial: experiences, challenges and lessons from the mixed-methods Jack Feasibility Trial. *Trials* 2016;17:365 doi: 10.1186/s13063-016-1506-y [published Online First 29 July 2016].

55. Hoffmann TC, Glasziou PP, Boutron I, et al. Better reporting of interventions: template for intervention description (TIDieR) checklist and guide. *BMJ* 2014;348:g1687 doi: 10.1136/bmj.g1687 [published Online First 07 March 2014].

56. Ajzen I, Madden TJ. Prediction of goal-directed behavior: Attitudes, intentions, and perceived behavioral control. *J Exp Soc Psychol* 1986;22:453–74 doi: 10.1016/0022-1031(86)90045-4 [published Online First 27 August 2004].

57. Ajzen I. The theory of planned behaviour: reactions and reflections. *Psychol Health* 2011;26:1113–27 doi: 10.1080/08870446.2011.613995 [published Online First 20 September 2011].

For peer review only

For peer review only - http://bmjopen.bmj.com/site/about/guidelines.xhtml

58. NHS Health Scotland. An overview of the effectiveness of sexual health improvement interventions. Final Report. June 2010 http://www.healthscotland.com/uploads/documents/13786-REO28-OverviewEffectivenessSexualHealthInterventions.pdf [Accessed September 2017].

59. Moore G, Evans R. What theory, for whom and in which context? Reflections on the application of theory in the development and evaluation of complex population health intervention. *SSM - Population Health* 2017;3:132-5 doi: 10.1016/j.ssmph.2016.12.005 [published Online First 13 December 2016].

60. Philliber S, Kaye JW, Herrling S, West E. Preventing pregnancy and improving health care access among teenagers: An evaluation of the Children's Aid Society-Carrera Program. *Perspect Sex Reprod Health* 2002;244–51 doi: 10.1363/3424402 [published Online First 1 September 2002].

61. Coyle K, Basen-Engquist K, Kirby D, et al. Safer choices: reducing teen pregnancy, HIV, and STDs. Public Health Rep 2001;116(Suppl 1):82–93 doi: 10.1093/phr/116.S1.82

62. Aventin Á, Lohan M, O'Halloran P, et al. Design and development of a film-based Intervention about teenage men and unintended pregnancy: Applying the Medical Research Council Framework in practice. *Eval Progr Plan* 2015;49:19–30 doi: 10.1016/j.evalprogplan.2014.11.003 [published Online First 15 November 2014].

63. NHS Health Scotland. Evaluation of Healthy Respect Phase Two: Final Report March 2010 http://www.healthscotland.com/uploads/documents/12159-HealthyRespectEvaluationReport_3342.pdf [Accessed September 2017].

64. Guttmacher Institute. Adolescent sexual and reproductive health in the United States https://www.guttmacher.org/fact-sheet/american-teens-sexual-and-reproductive-health [Accessed September 2017].

65. Kirby D. Sexuality Education: An evaluation of programs and their Effects. An executive summary. 1984. Washington DC: Network Publications.

For peer review only - http://bmjopen.bmj.com/site/about/guidelines.xhtml

66. Pleck JH, Sonenstein FL, Ku LC. Masculinity ideology: Its impact on adolescent males' heterosexual relationships. *J Soc Issues* 1993;49:1-29 doi: 10.1111/j.1540-4560.1993.tb01166.x [published Online First 14 April 2010].

67. Lottes I, Kuriloff P. Sexual socialization differences by gender, Greek membership, ethnicity and religious background. *Psychol Women Quar* 1994;18:203-19 doi: 10.1111/j.1471-6402.1994.tb00451.x {publish Online First 28 July 2006].

68. Quinn-Nilas C, Milhausen RR, Breuer R, Bailey J, Pavlou M, DiClemente RJ, Wingood GM. Validation of the Sexual Communication Self-Efficacy Scale. Health Education & Behavior. 2016 Apr;43(2):165-71.

69. May C. Towards a general theory of implementation. *Implement Sci* 2013;8:18 doi: 10.1186/1748-5908-8-18 [published Online First 13 February 2013].

70. Moore GF, Audrey S, Barker M, et al. Process evaluation of complex interventions: Medical Research Council guidance. *BMJ* 2015;350:h1258 doi: 10.1136/bmj.h1258 [published 19 March 2015].

71. National Institute for Health and Care Excellence. Methods for the development of NICE Public Health Guidance (third edition) https://www.nice.org.uk/process/pmg4/chapter/introduction [published September 2012, accessed September 2017].

72. Edwards RT, Charles JM, Lloyd-Williams H. Public health economics: a systematic review of guidance for the economic evaluation of public health interventions and discussion of key methodological issues. *BMC Public Health* 2013;13:1001 doi: 10.1186/1471-2458-13-1001 [published Online First 24 October 2013].

For peer review only - http://bmjopen.bmj.com/site/about/guidelines.xhtml

73. Bailey J, Mann S, Wayal S, et al. Sexual health promotion for young people delivered via digital media: a scoping review. *Public Health Res* 2015;3 doi: 10.3310/phr03130 [published Online First November 2015].

74. Campbell MK, Piaggio G, Elbourne DR, et al. Consort 2010 statement: extension to cluster randomised trials. *BMJ* 2012;345:e5661 doi: 10.1136/bmj.e5661 [published Online First 04 September 2012].

75. Kahan BC, Jairath V, Doré CJ, et al. The risks and rewards of covariate adjustment in randomized trials: an assessment of 12 outcomes from 8 studies. *Trials* 2014;15:1 doi: 10.1186/1745-6215-15-139 [published Online First 23 April 2014].

76. Kraemer HC, Wilson GT, Fairburn CG, et al. Mediators and moderators of treatment effects in randomized clinical trials. *Arch Gen Psychiatry* 2002;59:877–83 doi: 10.1001/archpsyc.59.10.877

77. Braun V, Clarke V. Using thematic analysis in psychology. *Qual Res Psychol* 2006;3:77–101 doi: 10.1191/1478088706qp063oa [published Online First 21 July 2008].

78. Lincoln YS, Guba EG. Naturalistic Inquiry. Newbury Park CA: Sage; 1985.

79. Hyde A, Howlett E, Brady D, et al. The focus group method: Insights from focus group interviews on sexual health with adolescents. *Soc Sci Med* 2005;61:2588–99 doi: https://doi.org/10.1016/j.socscimed.2005.04.040 [published Online First 13 June 2005].

80. Department of Education NI. Institution search. http://apps.education-ni.gov.uk/appinstitutes/default.aspx [Accessed October 2017].

81. Schulz KF, Altman DG, Moher D, for the CONSORT Group. CONSORT 2010 Statement: updated guidelines for reporting parallel group randomised trials. *BMJ* 2010;340:c332 doi: 10.1136/bmj.c332 [published Online First 24 March 2010].

For peer review only

For peer review only - http://bmjopen.bmj.com/site/about/guidelines.xhtml

82. Chan A-W, Tetzlaff JM, Altman, DG, et al. SPIRIT 2013 Statement: Defining standard protocol items for clinical trials. *Ann Intern Med* 2013;158:200-7 doi: 10.7326/0003-4819-158-3-201302050-00583

83. Chan A-W, Tetzlaff JM, Gøtzsche PC, et al. SPIRIT 2013 explanation and elaboration: guidance for protocols of clinical trials. *BMJ* 2013;346:e7568 doi: 10.1136/bmj.e7586 [published Online First 09 January 2013].

84. WHO Trial Registration Data Set (Version 1.2.1). Available at http://www.who.int/ictrp/network/trds/en/ [Accessed 07.08.17]

FIGURE LEGENDS

Figure 1: The JACK Trial Flowchart

Figure 2: School recruitment strategy

Figure 3: *If I Were Jack* Theory of Change Logic Model

Figure 4: Integration of process evaluation with experimental design methodology to achieve research objectives

Figure 5: Current and future impacts of *If I Were Jack* on costs and benefits

For peer review only

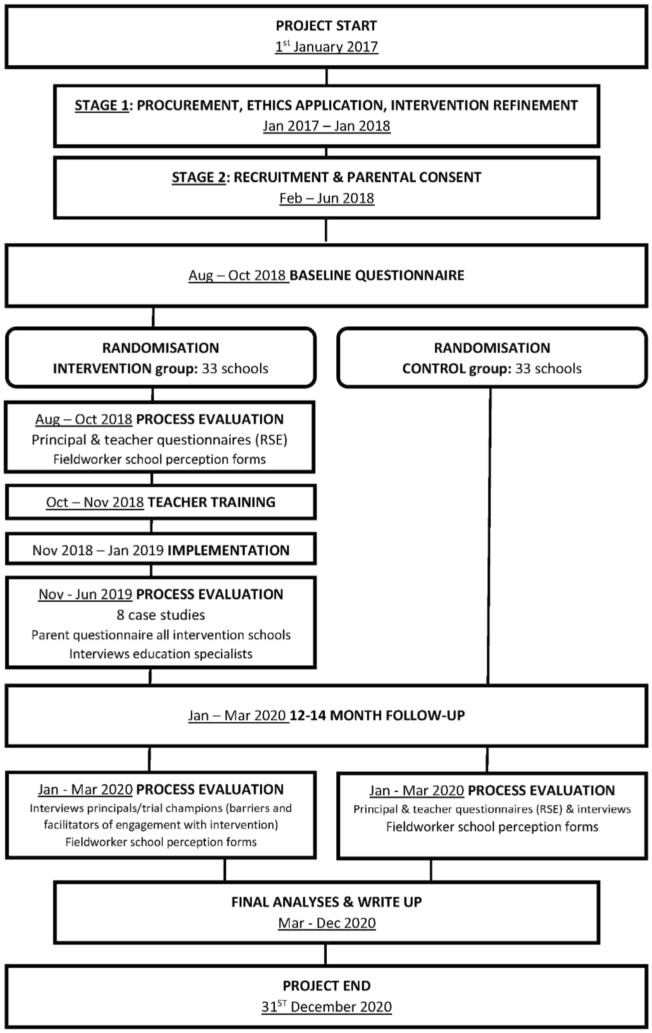

Figure 1: The JACK Trial Flowchart

210x297mm (300 x 300 DPI)

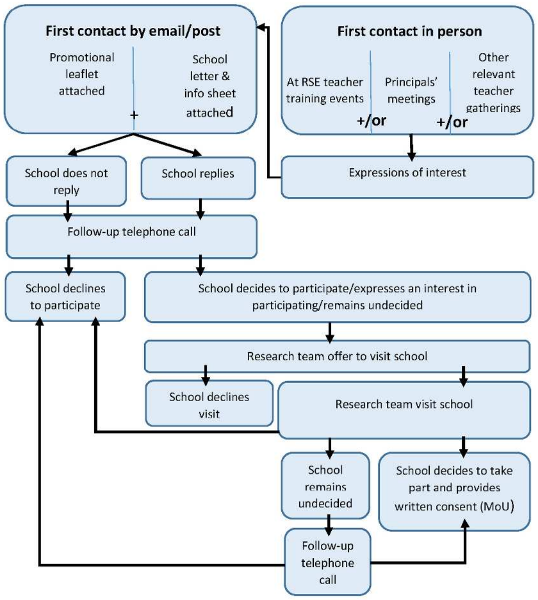

Figure 2: School recruitment strategy

210x297mm (300 x 300 DPI)

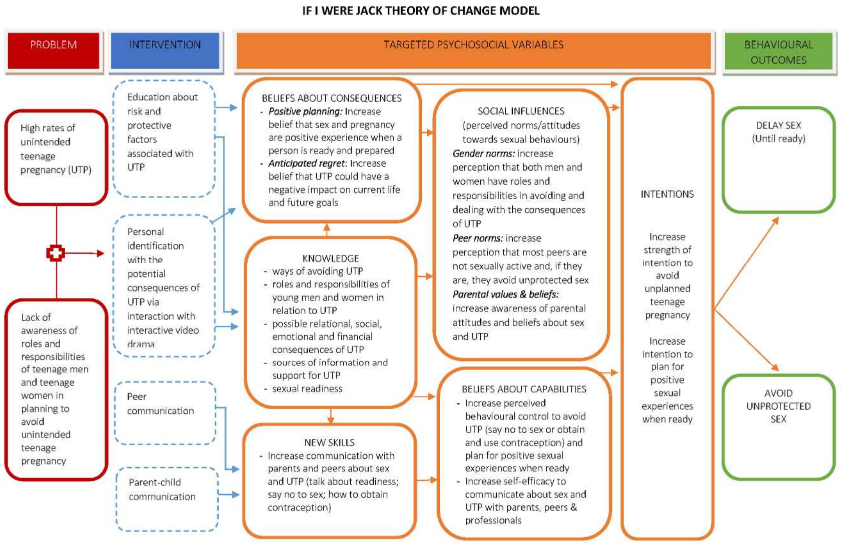

Figure 3: If I Were Jack Theory of Change Logic Model

297x210mm (300 x 300 DPI)

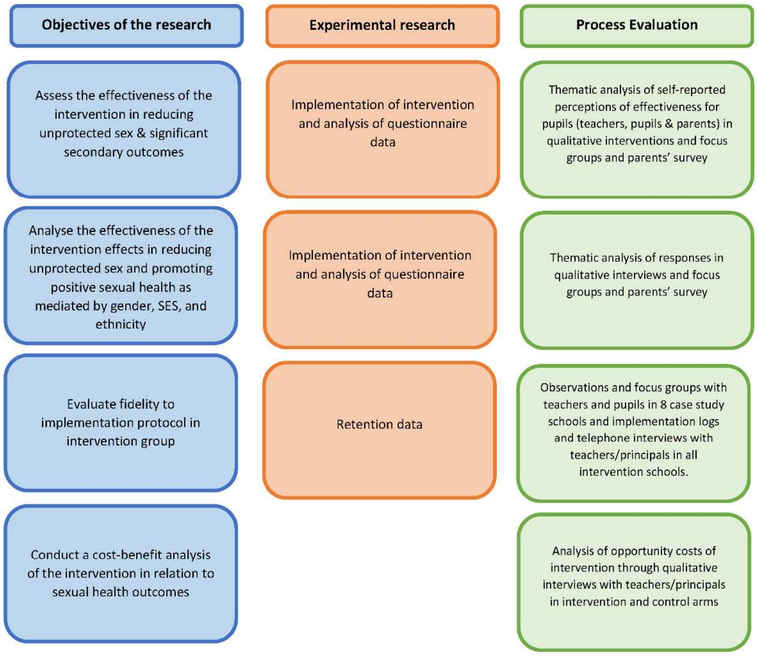

Figure 4: Integration of process evaluation with experimental design methodology to achieve research objectives

210x297mm (300 x 300 DPI)

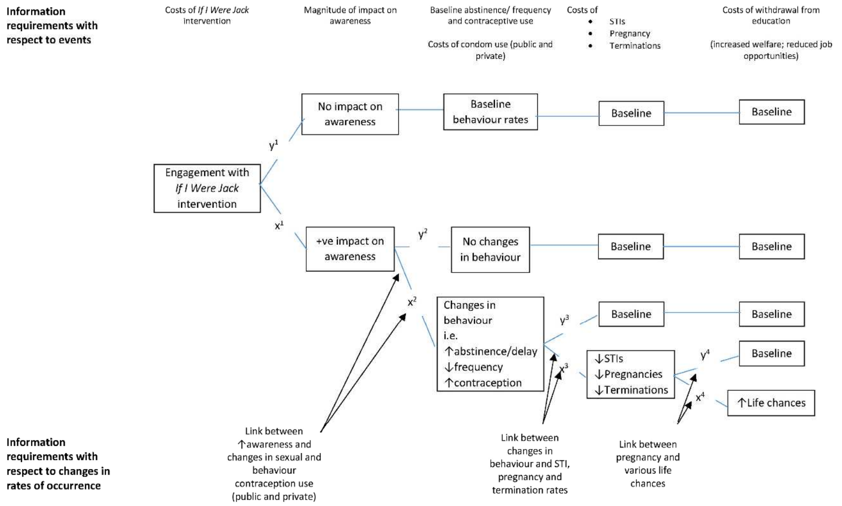

Figure 5: Current and future impacts of If I Were Jack on costs and benefits

297x210mm (300 x 300 DPI)

For peer review only - http://bmjopen.bmj.com/site/about/guidelines.xhtml

SPIRIT

STANDARD PROTOCOL ITEMS: RECOMMENDATIONS FOR INTERVENTIONAL TRIALS

SPIRIT 2013 Checklist: Recommended items to address in a clinical trial protocol and related documents*

Section/item	Item No	Description	Addressed on page number
**Administrative information**			
Title	1	Descriptive title identifying the study design, population, interventions, and, if applicable, trial acronym	______1_______
Trial registration	2a	Trial identifier and registry name. If not yet registered, name of intended registry	______2_______
	2b	All items from the World Health Organization Trial Registration Data Set	22 (sup file 3)
Protocol version	3	Date and version identifier	______________
Funding	4	Sources and types of financial, material, and other support	______24_______
Roles and responsibilities	5a	Names, affiliations, and roles of protocol contributors	______1, 24_____
	5b	Name and contact information for the trial sponsor	______________
	5c	Role of study sponsor and funders, if any, in study design; collection, management, analysis, and interpretation of data; writing of the report; and the decision to submit the report for publication, including whether they will have ultimate authority over any of these activities	_____24________
	5d	Composition, roles, and responsibilities of the coordinating centre, steering committee, endpoint adjudication committee, data management team, and other individuals or groups overseeing the trial, if applicable (see Item 21a for data monitoring committee)	______________

For peer review only - http://bmjopen.bmj.com/site/about/guidelines.xhtml

For peer review only

Introduction

Background and rationale	6a	Description of research question and justification for undertaking the trial, including summary of relevant studies (published and unpublished) examining benefits and harms for each intervention	_____4,5______
	6b	Explanation for choice of comparators	_____4,5______
Objectives	7	Specific objectives or hypotheses	_____5,6_____
Trial design	8	Description of trial design including type of trial (eg, parallel group, crossover, factorial, single group), allocation ratio, and framework (eg, superiority, equivalence, noninferiority, exploratory)	_____6_____

Methods: Participants, interventions, and outcomes

Study setting	9	Description of study settings (eg, community clinic, academic hospital) and list of countries where data will be collected. Reference to where list of study sites can be obtained	_____6________
Eligibility criteria	10	Inclusion and exclusion criteria for participants. If applicable, eligibility criteria for study centres and individuals who will perform the interventions (eg, surgeons, psychotherapists)	_____6,7,13_____
Interventions	11a	Interventions for each group with sufficient detail to allow replication, including how and when they will be administered	_____10-13_____
	11b	Criteria for discontinuing or modifying allocated interventions for a given trial participant (eg, drug dose change in response to harms, participant request, or improving/worsening disease)	______________
	11c	Strategies to improve adherence to intervention protocols, and any procedures for monitoring adherence (eg, drug tablet return, laboratory tests)	_____16________
	11d	Relevant concomitant care and interventions that are permitted or prohibited during the trial	______________
Outcomes	12	Primary, secondary, and other outcomes, including the specific measurement variable (eg, systolic blood pressure), analysis metric (eg, change from baseline, final value, time to event), method of aggregation (eg, median, proportion), and time point for each outcome. Explanation of the clinical relevance of chosen efficacy and harm outcomes is strongly recommended	____14,15_____
Participant timeline	13	Time schedule of enrolment, interventions (including any run-ins and washouts), assessments, and visits for participants. A schematic diagram is highly recommended (see Figure)	___Figure 1_____

For peer review only - http://bmjopen.bmj.com/site/about/guidelines.xhtml

Sample size	14	Estimated number of participants needed to achieve study objectives and how it was determined, including clinical and statistical assumptions supporting any sample size calculations	____7,8______
Recruitment	15	Strategies for achieving adequate participant enrolment to reach target sample size	____8-10_______

Methods: Assignment of interventions (for controlled trials)

Allocation:			
Sequence generation	16a	Method of generating the allocation sequence (eg, computer-generated random numbers), and list of any factors for stratification. To reduce predictability of a random sequence, details of any planned restriction (eg, blocking) should be provided in a separate document that is unavailable to those who enrol participants or assign interventions	____10_______
Allocation concealment mechanism	16b	Mechanism of implementing the allocation sequence (eg, central telephone; sequentially numbered, opaque, sealed envelopes), describing any steps to conceal the sequence until interventions are assigned	____10______
Implementation	16c	Who will generate the allocation sequence, who will enrol participants, and who will assign participants to interventions	____10______
Blinding (masking)	17a	Who will be blinded after assignment to interventions (eg, trial participants, care providers, outcome assessors, data analysts), and how	_____6________
	17b	If blinded, circumstances under which unblinding is permissible, and procedure for revealing a participant's allocated intervention during the trial	_____________

Methods: Data collection, management, and analysis

Data collection methods	18a	Plans for assessment and collection of outcome, baseline, and other trial data, including any related processes to promote data quality (eg, duplicate measurements, training of assessors) and a description of study instruments (eg, questionnaires, laboratory tests) along with their reliability and validity, if known. Reference to where data collection forms can be found, if not in the protocol	____15-20_____
	18b	Plans to promote participant retention and complete follow-up, including list of any outcome data to be collected for participants who discontinue or deviate from intervention protocols	_____8-10_____

For peer review only - http://bmjopen.bmj.com/site/about/guidelines.xhtml

Data management	19	Plans for data entry, coding, security, and storage, including any related processes to promote data quality (eg, double data entry; range checks for data values). Reference to where details of data management procedures can be found, if not in the protocol	_____________
Statistical methods	20a	Statistical methods for analysing primary and secondary outcomes. Reference to where other details of the statistical analysis plan can be found, if not in the protocol	___20-21____
	20b	Methods for any additional analyses (eg, subgroup and adjusted analyses)	___20-21____
	20c	Definition of analysis population relating to protocol non-adherence (eg, as randomised analysis), and any statistical methods to handle missing data (eg, multiple imputation)	___20-21____
**Methods: Monitoring**			
Data monitoring	21a	Composition of data monitoring committee (DMC); summary of its role and reporting structure; statement of whether it is independent from the sponsor and competing interests; and reference to where further details about its charter can be found, if not in the protocol. Alternatively, an explanation of why a DMC is not needed	____23_________
	21b	Description of any interim analyses and stopping guidelines, including who will have access to these interim results and make the final decision to terminate the trial	____23______
Harms	22	Plans for collecting, assessing, reporting, and managing solicited and spontaneously reported adverse events and other unintended effects of trial interventions or trial conduct	____23_________
Auditing	23	Frequency and procedures for auditing trial conduct, if any, and whether the process will be independent from investigators and the sponsor	_____________
**Ethics and dissemination**			
Research ethics approval	24	Plans for seeking research ethics committee/institutional review board (REC/IRB) approval	____23_____
Protocol amendments	25	Plans for communicating important protocol modifications (eg, changes to eligibility criteria, outcomes, analyses) to relevant parties (eg, investigators, REC/IRBs, trial participants, trial registries, journals, regulators)	____22_______

For peer review only - http://bmjopen.bmj.com/site/about/guidelines.xhtml

Consent or assent	26a	Who will obtain informed consent or assent from potential trial participants or authorised surrogates, and how (see Item 32)	____8-10______
	26b	Additional consent provisions for collection and use of participant data and biological specimens in ancillary studies, if applicable	______________
Confidentiality	27	How personal information about potential and enrolled participants will be collected, shared, and maintained in order to protect confidentiality before, during, and after the trial	______________
Declaration of interests	28	Financial and other competing interests for principal investigators for the overall trial and each study site	____24_________
Access to data	29	Statement of who will have access to the final trial dataset, and disclosure of contractual agreements that limit such access for investigators	_____23________
Ancillary and post-trial care	30	Provisions, if any, for ancillary and post-trial care, and for compensation to those who suffer harm from trial participation	______________
Dissemination policy	31a	Plans for investigators and sponsor to communicate trial results to participants, healthcare professionals, the public, and other relevant groups (eg, via publication, reporting in results databases, or other data sharing arrangements), including any publication restrictions	_____23________
	31b	Authorship eligibility guidelines and any intended use of professional writers	_____24,25____
	31c	Plans, if any, for granting public access to the full protocol, participant-level dataset, and statistical code	______________
**Appendices**			
Informed consent materials	32	Model consent form and other related documentation given to participants and authorised surrogates	______________
Biological specimens	33	Plans for collection, laboratory evaluation, and storage of biological specimens for genetic or molecular analysis in the current trial and for future use in ancillary studies, if applicable	______________

*It is strongly recommended that this checklist be read in conjunction with the SPIRIT 2013 Explanation & Elaboration for important clarification on the items. Amendments to the protocol should be tracked and dated. The SPIRIT checklist is copyrighted by the SPIRIT Group under the Creative Commons "Attribution-NonCommercial-NoDerivs 3.0 Unported" license.

For peer review only - http://bmjopen.bmj.com/site/about/guidelines.xhtml
