# Supplementary material for: JACK trial protocol: a phase III multicentre cluster randomised controlled trial of a school-based relationship and sexuality education intervention focusing on young male perspectives
Source: BMJ Open. 2018 Jul 28;8(7):e022128. doi: 10.1136/bmjopen-2018-022128 (PMC6067362; doi:10.1136/bmjopen-2018-022128)
Supplement: Reviewer comments [file bmjopen-2018-022128.reviewer_comments.pdf]

PEER REVIEW HISTORY

BMJ Open publishes all reviews undertaken for accepted manuscripts. Reviewers are asked to complete a checklist review form (**http://bmjopen.bmj.com/site/about/resources/checklist.pdf**) and are provided with free text boxes to elaborate on their assessment. These free text comments are reproduced below.

ARTICLE DETAILS

**TITLE (PROVISIONAL)**	The JACK Trial Protocol: A Phase III multi-centre cluster randomised controlled trial of a school-based relationship and sexuality education intervention focusing on young male perspectives
**AUTHORS**	Lohan, Maria; Aventin, Áine; Clarke, Mike; Curran, Rhonda; Maguire, Lisa; Hunter, Rachael; McDowell, Clíona; McDaid, Lisa; Young, Honor; White, James; Fletcher, Adam; French, Rebecca; Bonell, Christopher; Bailey, Julia; O'Hare, Liam

This article was not externally reviewed at BMJ Open. Protocols that have been independently assessed prior to submission to BMJ Open are usually fast-tracked to publication on the grounds that further substantial changes will not be possible. This independent assessment will usually be external, independent review for both a substantial grant award from a non-commercial or government funder and ethics approval.
